# Combined [^18^F]Fluorodeoxyglucose PET and [^123^I]Iodometomidate-SPECT for diagnostic evaluation of indeterminate adrenal neoplasias—the cross-sectional diagnostic test accuracy study FAMIAN

**DOI:** 10.1016/j.ebiom.2025.105735

**Published:** 2025-05-20

**Authors:** Stefanie Hahner, Philipp Hartrampf, Felix Beuschlein, Matthias Miederer, Konstanze Miehle, Wiebke Schlötelburg, Carmina Teresa Fuß, Thomas Pfluger, Christian Fottner, Anke Tönjes, Ken Herrmann, Holger Amthauer, Martin Reincke, Mathias Schreckenberger, Osama Sabri, Johanna Werner, Miriam Reuter, Stefan Kircher, Wiebke Arlt, Martin Fassnacht, Andreas Konrad Buck, Hans-Helge Müller, Andreas Schirbel, FAMIAN investigators, Christian Furth, Knut Mai, Marcus Quinkler, Frank Weber, Henning Dralle, Benjamin Sandner, Thomas Lincke, Regine Kluge, Nada Rayes, Matthias Weber, Martina Gräsl, Wolfgang Saeger, Joachim Reibetanz

**Affiliations:** a Division of Endocrinology and Diabetes, Department of Internal Medicine I, University Hospital, University of Würzburg, Würzburg, Germany; b Department of Nuclear Medicine, University Hospital, University of Würzburg, Würzburg, Germany; c Klinik für Endokrinologie, Diabetologie und Klinische Ernährung, UniversitätsSpital Zürich (USZ) und Universität Zürich (UZH), Zurich, Switzerland; d The LOOP Zurich - Medical Research Center, Zurich, Switzerland; e Medizinische Klinik und Poliklinik IV, LMU Klinikum, Ludwig-Maximilians-Universität München, Munich, Germany; f Department of Translational Imaging in Oncology, National Center for Tumour Diseases (NCT/UCC) Dresden, Faculty of Medicine and University Hospital Carl Gustav Carus, University of Technology Dresden (TUD), Dresden, Germany; g German Cancer Research Center (DKFZ) Heidelberg, Heidelberg, Germany; h Helmholtz-Zentrum Dresden-Rossendorf (HZDR), Dresden, Germany; i Department of Nuclear Medicine, University Medical Center of the Johannes Gutenberg University Mainz, Mainz, Germany; j Medical Department III- Endocrinology, Nephrology, Rheumatology, University of Leipzig Medical Center, Leipzig, Germany; k Department of Nuclear Medicine, University Hospital, Ludwig-Maximilians-Universität Munich, Germany; l Department of Endocrinology and Metabolism, I Medical Clinic, University Medical Center of the Johannes Gutenberg University Mainz, Germany; m Department of Nuclear Medicine, West German Cancer Center (WTZ), University Hospital Essen, University of Duisburg-Essen; German Cancer Consortium (DKTK), Partner Site University Hospital Essen, Essen, Germany; n Charité - Universitätsmedizin Berlin, Freie Universität Berlin, Humboldt-Universität zu Berlin, and Berlin Institute of Health, Department of Nuclear Medicine, Berlin, Germany; o Department of Nuclear Medicine, University Hospital Leipzig, Germany; p Institute of Pathology, University of Würzburg, Würzburg, Germany; q Medical Research Council Laboratory of Medical Sciences, London, UK; r Institute of Clinical Sciences, Faculty of Medicine, Imperial College London, London, UK; s Institute of Medical Bioinformatics and Biostatistics, Philipps University of Marburg, Germany

**Keywords:** Adrenal incidentaloma, Molecular imaging, Fluorodeoxyglucose positron emission tomography, Computed tomography, Iodometomidate SPECT, Adrenocortical adenoma, Adrenocortical carcinoma, Indeterminate adrenal masses

## Abstract

**Background:**

Adrenal tumours are frequently detected by conventional imaging. However, computed tomography and magnet resonance imaging have limited specificity in classifying the most prevalent tumour type, adrenocortical adenoma (ACA), which typically does not require surgery. We proposed that combined molecular imaging with [^18^F]Fluorodeoxyglucose-positron emission tomography (FDG PET) and [^123I^]Iodometomidate-single photon emission tomography (IMTO SPECT) improves non-invasive classification of ACA.

**Methods:**

This cross-sectional, multicentre diagnostic study included patients (≥30 years) with non-functioning indeterminate adrenal masses (>3 cm or increase >1 cm, Hounsfield units [HU] ≥10 on unenhanced computed tomography [CT]) scheduled for surgery. Using histopathology as the reference, we assessed the accuracy of FDG/IMTO imaging as an ACA-test, assuming that low FDG with high IMTO uptake is indicative of ACA, with a focus on high specificity and moderate to high sensitivity. We also investigated its accuracy in detecting or excluding adrenocortical carcinoma (ACC) and evaluated FDG and unenhanced CT in assessing malignancy. Trial-registration: EudraCT 2012-003604-13; ClinicalTrials.gov-identifier NCT02010957.

**Findings:**

From July 2015 to December 2020, 85 patients were enrolled, with 77 included in the final analysis (53 benign, 30 ACA, 9 ACC). FDG/IMTO-imaging classified ACA with high specificity (95·7% [95% CI 85·2%–99·47%]), high positive predictive value (87·5% [95% CI 61·7%–98·4%]) and high positive likelihood ratio (11·1 [95% CI 3·2–122]). However, sensitivity was low (48·3% [95% CI 29·4%–67·5%]) due to moderate/high FDG uptake in 14 of 30 ACA. Malignant masses were classified with high sensitivity but low-to-moderate specificity by both unenhanced CT (cut-off HU ≥20 sensitivity 100% [95% CI 85·8%–100%], specificity 26·4% [95% CI 15·3%–40·3%]) and FDG (visual analysis sensitivity 95·8% [95% CI 78·9%–99·9%], specificity 62·3% [95% CI 47·9%–75·2%]). All four study-related AEs were grade 1, the seven serious AEs were not study-related.

**Interpretation:**

Combined FDG/IMTO-imaging classifies ACA with high specificity, potentially reducing unnecessary surgery. A sub-group of FDG-positive ACA lowers sensitivity.

**Funding:**

10.13039/501100001659German Research Foundation and EU-FP7.

**Research in context**

**Evidence before this study**

Tumours of the adrenal gland are common and often discovered incidentally. Current guidelines recommend additional diagnostic tests to determine whether surgical removal of the tumour is required on the basis of hormone excess or malignant potential. In the vast majority of cases, however, incidentally discovered adrenal masses are benign adrenocortical adenomas (ACA) that do not necessitate further intervention. Non-invasive methods for a reliable and highly specific classification of these ACA have not yet been established. Before initiation of the FAMIAN study, we searched Pubmed, the Cochrane library and Science citation index for publications from January 1st 1970 to January 1st 2015 using combinations of the search terms “adrenal incidentaloma”, “adrenal imaging”, “metomidate”, “etomidate”, “FDG PET”, “molecular imaging”. In addition, in preparation of the study, we organised an ENSAT (European Network for the Study of Adrenal Tumours) Imaging Study Group meeting at the European Conference on Adrenal Imaging, bringing together leading experts in the field of adrenal imaging from several European countries. While FDG PET/CT diagnostics revealed to be the best-established functional imaging method so far to differentiate benign from malignant processes, molecular imaging with the CYP11B1/2 ligand metomidate had been shown to discriminate between adrenocortical and non-adrenocortical lesions in several studies. Two studies, using both FDG and [^11^C]metomidate imaging included a total of 35 patients. Both revealed that [^11^C]metomidate distinguishes between adrenocortical and non-adrenocortical lesions, whereas FDG proved valuable in discriminating benign from malignant tumours. The available series investigating FDG- or metomidate-based molecular imaging in adrenal tumours included tumours for which the question of malignant potential or adrenocortical origin could already be approximated based on conventional imaging results such as unenhanced CT tumour attenuation values ≤10 Hounsfield Units (HU) or biochemical and/or clinical evidence of adrenal steroid excess. In addition, final diagnosis based on histopathology was often only available for a subgroup of investigated patients. Analysing the clinical utility of FDG and metomidate tracers for adrenal molecular imaging and particularly the significance of their combined use requires a prospective study in the most relevant patient group, i.e. patients with adrenal incidentalomas without relevant steroid hormone excess but with uncertain malignant potential based on the results of conventional cross-sectional imaging, i.e. CT or MRI. We hypothesised that adrenal lesions with low FDG uptake and high metomidate uptake would have a high probability of representing an ACA and, thus, not require adrenalectomy.

**Added value of this study**

In this multicentre, investigator-initiated diagnostic study, we assessed the diagnostic accuracy of combined [^18^F]Fluorodexoyglucose-positron emission tomography (FDG PET) and [^123^I]Iodometomidate-single photon emission tomography (IMTO SPECT) imaging in patients with indeterminate adrenal masses. [^123^I]IMTO was chosen because of its broader applicability compared to [^11^C]metomidate, which is limited by the need for local availability of a cyclotron due to half-life of only about 20 min of carbon-11. No previous study has investigated this and there is also no other study on any tool for non-invasive classification of ACA in indeterminate adrenal masses. We demonstrate that combined FDG-/IMTO-imaging is of clinical value. However, sensitivity of the combined molecular imaging test was limited by a sub-group of ACA with high FDG uptake, which warrants further investigation. Furthermore, we provide information on the distribution of underlying histopathological diagnoses in this specific group of indeterminate non-functioning adrenal lesions and additional information on the diagnostic accuracy of unenhanced CT and FDG in assessing malignancy in adrenal masses, observing high sensitivity but only low specificity for both FDG and particularly for unenhanced CT.

**Implications of all the available evidence**

This cross-sectional diagnostic study systematically evaluated the use of molecular imaging to differentiate indeterminate adrenal masses, which have traditionally been treated with surgical removal despite most being benign adrenocortical adenomas (ACA). Implementing this innovative diagnostic method may help to reduce unnecessary surgeries, thereby improving health outcomes and lowering healthcare costs.

## Introduction

Adrenal tumours are detected with increasing frequency in the context of an ageing population and broadening use of cross-sectional imaging, posing a growing public health challenge. The prevalence of adrenal incidentalomas in imaging studies is around 3% in adults over 50 years and up to 10% in patients over 80 years of age.^1^ Adrenocortical adenoma (ACA) is the most common underlying entity, followed by metastases, adrenocortical carcinoma (ACC), phaeochromocytomas and rarer entities.^2^, ^3^, ^4^ Phaeochromocytomas and adrenocortical masses causing hormone excess can be identified biochemically. However, most adrenal masses do not cause signs and symptoms of hormone excess. The clinical challenge lies in determining the optimal therapeutic approach: adrenalectomy is performed for tumours causing overt hormone excess. It is also considered in non-functioning tumours depending on the perceived risk for malignancy based on imaging findings. Estimating the malignant potential of an adrenal tumour can be challenging, leading to unnecessary surgery, as a significant number of tumours are ultimately found to be benign on histopathology.^5^^,^^6^ Assessment of tissue density by determination of the tumour attenuation value in Hounsfield Units (HU) in unenhanced computed tomography (CT) is well established, whereby a cut-off value ≤10 is highly indicative of a benign tumour.^3^^,^^6^^,^^7^ However, more than 40% of benign tumours exceed this threshold.^3^^,^^6^^,^^8^^,^^9^ Raising the threshold from 10 to 20 HUs significantly improves specificity for detection of malignancy with minimal loss of sensitivity.^6^^,^^8^^,^^9^ Nevertheless, even in tumours with an attenuation value >20 HU, ACA is still the most common entity.^3^^,^^10^

As complementary imaging options to estimate the malignant potential of adrenal lesions, magnetic resonance imaging (MRI) with chemical shift, analysis of contrast washout in serial CT and [^18^F]FDG PET imaging have been utilised.^1^ However, data regarding the diagnostic utility of these imaging methods in differentiating benign from malignant adrenal lesions is generally scarce.^7^ Furthermore, the additional benefit for wash-out CT was found to be limited.^10^, ^11^, ^12^ Current evidence supports FDG PET imaging,^13^, ^14^, ^15^, ^16^, ^17^, ^18^, ^19^ showing high sensitivity for classification of malignant tumours while reaching moderate to high specificity.

However, most studies included patients who were either already surgical candidates due to hormone hypersecretion or in whom surgery was not required due to unenhanced tumour attenuation <10 HU. Consequently, the number of individuals reported in whom FDG imaging was relevant for therapeutic guidance has been limited. Considering the high proportion of patients with ACA even after preselection by HU cutoffs, a non-invasive method to classify ACA with high validity would be of major clinical value and avoid unnecessary adrenalectomy. As management strategies for ACC differ from those for non-adrenocortical (non-AC) malignant adrenal masses, non-invasive characterisation of these two tumour categories would be of additional value.

Metomidate and its analogous tracer compounds specifically bind to the cytochrome P450 enzymes 11β-hydroxylase (CYP11B1) and aldosterone synthase (CYP11B2) in adrenocortical cells.^20^, ^21^, ^22^, ^23^, ^24^, ^25^, ^26^, ^27^, ^28^

We aimed at evaluation of combined use of [^18^F]FDG PET and [^123^I]Iodometomidate-(IMTO) SPECT imaging for non-invasive classification of such adrenal masses in a multicentre cross-sectional diagnostic study. The primary objective was to determine the accuracy of a combined imaging test for classification of ACA, aiming to achieve high specificity (above 90%) and a high likelihood ratio (above 9). We proposed to reach a positive predictive value >90%, indicating high clinical utility.

## Methods

### Study design and participants

The Combined 18F-Fluorodeoxyglucose (FDG) Positron Emission Tomography (PET) And Metomidate Imaging for Adrenal Neoplasia (FAMIAN) study was designed as a prolective (in the sense that the data acquisition takes place after the study planning), cross-sectional, multicentre diagnostic test study assessing the accuracy of combined FDG-/IMTO-imaging in classifying an indeterminate adrenal mass as ACA or non-ACA (primary diagnostic test). Low FDG uptake (FDG-negative) with high IMTO uptake (IMTO-positive) was considered diagnostic for a benign adrenocortical adenoma (ACA). We also assessed the accuracy of FDG and unenhanced CT tumour attenuation regarding the diagnosis of malignancy.

Participants presenting at participating study centres were eligible for recruitment if they fulfilled the following inclusion criteria: diagnosis of an adrenal mass of more than 3 cm diameter (or increase in tumour diameter by more than 1 cm upon follow-up imaging) and indeterminate imaging findings defined by a tumour attenuation ≥10 HU on unenhanced CT. Recruitment was restricted to adults ≥30 years both for reasons of radiation protection and as large and/or growing adrenal masses in younger patients are invariably surgically removed.

Exclusion criteria were biochemical confirmation of phaeochromocytoma, primary aldosteronism or adrenal Cushing's syndrome, the latter defined by fulfilment of all three of the following criteria: 1) morning serum cortisol after 1 mg dexamethasone >5 μg/dl (140 nmol/L); 2) Plasma ACTH <5 ng/L; 3) urinary free cortisol levels twice the upper limit of normal AND/OR bedtime salivary cortisol thrice the upper limit of normal. Details in Appendix p 3.

### Study procedures

Study recruitment took place between July 20th 2015, and December 14th 2020 at six tertiary care endocrine specialist centres in Germany (Berlin, Essen, Leipzig, Mainz, Munich, Würzburg). All participants provided written informed consent before inclusion. The study was approved by the ethics committee of the University of Würzburg (No. 282/13_ff) and additionally at the local ethics committees at each trial site as well as by the Federal Institute for Drugs and Medical Devices, BfArM, No. 61-3910-4040442 and the Federal Institute for Radiation Protection, BfS, No. Z5-22463/2-2014-016). The study was registered at the European Union Drug Regulating Authorities Clinical Trials Database (EudraCT No. 2012-003604-13) and at ClinicalTrials.gov (identifier NCT02010957).

Four study visits were scheduled: screening visit (visit 1), diagnostic evaluation by FDG and IMTO imaging (visits 2 and 3), and follow-up visit for safety and adverse event assessment 2–4 weeks after visit 3 or preoperativley (visit 4). An optional visit 5 was foreseen 6–12 months after visit 3 in case no surgical intervention was performed. Adverse events (AE) were reported until 30 days after second imaging procedure or surgery—whichever came first. Details in Appendix p 3 and 4.

### Molecular imaging procedures

[^18F^]FDG PET was performed using state-of-the-art hybrid PET/CT devices. Static emission imaging was performed 1 h after i.v.-injection of FDG according to PET/CT scanners and guidelines.

[^123I^]Iodometomidate was prepared as previously described.^28^ IMTO SPECT was performed with state-of-the-art gamma cameras and acquisition settings adequate for I-123. After receiving 185 MBq [^123I^]IMTO i.v., planar scans of the whole body were acquired 4 h post injection using a standard technique. All patients also underwent SPECT/CT imaging between 4 and 6 h post injection. Transmission was measured by low-dose CT. Details in Appendix p 5.

### Centralised imaging review

The central imaging board was located at the University Hospital Würzburg. Review of imaging was undertaken by a multidisciplinary panel consisting of two endocrine specialists and two nuclear medicine physicians for all FDG PET scans, all IMTO whole-body planar scans, and all IMTO SPECT scans. All panel members were blinded to clinical information including histopathology. Decisions on visual image interpretation were made by consensus using a semi-quantitative scoring system ranging from 1 to 5: Visual scores of 1 and 2 indicate no or low tracer uptake (classified negative regarding tracer uptake), whereas scores 4 and 5 indicate moderate or high tracer uptake (positive). Tumours with intermediate tracer uptake were rated 3 (indeterminate). Regarding the management of lesions classified as indeterminate in the visual image analysis, the following criteria were established for subsequent statistical evaluation: FDG PET (aiming at high sensitivity for malignancy): FDG indeterminate was classified as malignant. IMTO SPECT (aiming at high specificity for adrenocortical origin): IMTO indeterminate was classified as non-adrenocortical. Quantitative analyses for tracer accumulation were performed by placing a volume-of-interest (VOI) around the adrenal mass and the reference (background) regions (contralateral adrenal, prevertebral region and liver). SUVmax, SUVmean and SUVpeak and respective tumour-to-background ratios (TBR) were determined. Details in Appendix p 5.

### Reference standard

The reference standard for ACA, non-adrenocortical benign, ACC, and non-adrenocortical malignant tumours was based on tumour histopathology. Paraffin blocks of the resected tumour tissue were re-analysed by one of two central reference pathologists without knowledge of the functional imaging results (Wolfgang Saeger, Hamburg, and Stefan Kircher, Würzburg). Details in Appendix p 5.

### Statistical analysis

We assumed that in at least half of patients with indeterminate adrenal masses, these masses are ACA, where surgery is unnecessary. We evaluated an ACA index test based on combined FDG PET and IMTO SPECT imaging for non-invasive classification of indeterminate adrenal masses. We conducted a cross-sectional evaluation to determine the test's accuracy. The primary objective was to assess the accuracy of the combined imaging test in classifying ACA (ACA+ and ACA-) and to demonstrate high specificity (>90%) and a high positive likelihood ratio (>9). We proposed to reach a positive predictive value >90%.

The confirmatory statistical analysis tested four null-hypotheses hierarchically, strongly maintaining a type I error level of α = 5% (2·5% for each side, the positive and the negative direction of the alternative hypotheses). The first two tests consider the accuracy measures of the diagnostic ACA-test, its specificity and likelihood ratio of a positive ACA-test (ACA+), respectively: The positive directions of the alternative hypotheses are specificity P (ACA−|non-ACA) > 90% (establishing a specificity of more than 90%) and positive likelihood ratio P (ACA+|ACA)/P (ACA+|non-ACA) >9, respectively. The third and fourth test focused on the ACA-test's ability to distinguish between ACC and non-ACC tumours, detecting ACC clearly as ACA− and detecting ACC considerably less likely than non-ACC as ACA+. The positive directions of the alternative hypotheses are P (ACA−|ACC) >80% and likelihood ratio P (ACA+|ACC)/P (ACA+|non-ACC) <7/19.

Rejection of the null-hypotheses in the positive directions indicates, after significant first test, a specificity over 90%, after significant second test–assuming a pre-test probability for ACA of at least 50%–a positive predictive value P (ACA|ACA+) over 90%, after significant third test a probability P (ACA−|ACC) of more than 80% that ACC is detected as ACA−, and after significant fourth test–assuming a pre-test probability for ACC of at most 12·5%–a predictive value P (ACC|ACA+) for ACC after positive ACA-test below 5%.

Statistical methods included exact binomial tests and Clopper-Pearson confidence intervals (CIs) for specificity, sensitivity, and probabilities. Likelihood ratios were tested and estimated with exact tests and CIs. ROC analyses determined the area under the curve (AUC) with 95% CIs. All two-sided 95% CIs are the intersections of two one-sided 97·5% CIs.

Analyses were performed with SAS, version 9·4 (SAS Institute, Cary, NC), StatXact of Cytel Studio, version 6·3·0 (Cytel Studio; Cytel Corporation, Cambridge, MA), GraphPad PRISM 6, IBM SPSS28, and the R Project for Statistical Computing.

Sample size calculation for the first two primary hierarchically structured two-sided level 5% tests on the accuracy of the diagnostic ACA-test assumed a 60% a priori probability for ACA. Detection of the first positive alternative hypothesis for the ACA-test with 80% power at 98% specificity, required about 195 participants, including at least 70 with confirmed non-ACA neoplasia for the exact statistical test. For the second positive alternative hypothesis for the ACA-test with 80% power, at 98% specificity, and 92% sensitivity, about 187 assessed participants were required.

Accounting for a 10% incomplete assessment rate, 220 participants were planned for enrolment, with a screening target of 550 patients assuming a 40% recruitment rate.

For the third and fourth hypotheses, power calculations based on 195 assessed participants, a 60% probability for ACA, and a 10% probability for ACC, indicated about 70% power for the third positive alternative hypothesis if P (ACA−|ACC) = 98% and nearly 80% power for the fourth positive alternative hypothesis if P (ACA−|ACC) = 98% and P (ACA+|non-ACC) = 61·5%.

### Role of the funding source

The funders of the study had no role in study design, data collection, data analysis, data interpretation, or writing of the report.

## Results

### Patient cohort

**Fig. 1:**
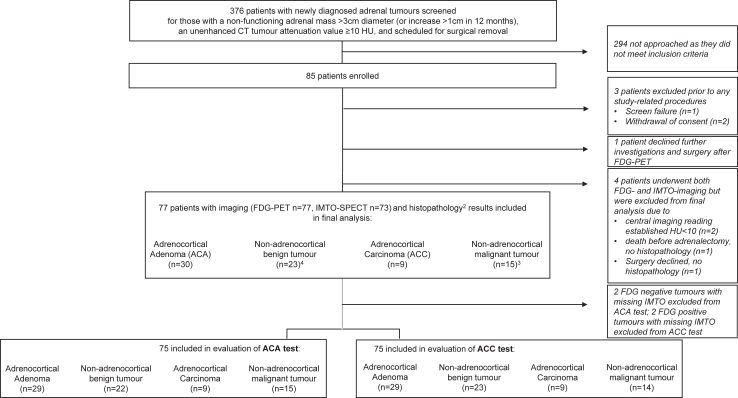
Flow of participants and main histopathological categories. Patients were included at six German study centres and completed 4 study visits: Visit 1: baseline evaluation; Visit 2 and 3: Diagnostic imaging with FDG and IMTO. The order of imaging was left to the decision of the recruiting centre. Visit 4: Follow up including routine laboratory parameters and adverse event assessment, 2–3 weeks after visit 3 or preoperative, whichever came first. In four cases, only FDG PET was available (1 ACA FDG+, 1 ACA FDG−, 1 non-AC benign, FDG−, 1 non-AC malignant, FDG+). For the evaluation of the ACA test, all 73 tumours with combined imaging information and both tumours with missing IMTO but FDG+ (classified as non-ACA) were included. For the evaluation of the ACC test, all 73 tumours with combined imaging information and both tumours with missing IMTO but available FDG- (classified as non-ACC) were included. 1: No IMTO SPECT due to delivery problems or failure of batch in final quality control in 2 ACA, 1 non-AC benign and 1 non-AC malignant tumour. 2: histopathology based on 72 surgical specimens and 5 biopsies. 3: non-AC malignant tumours comprised 8 metastases (2 malignant melanomas, 1 chorion carcinoma, 1 renal cell cancer, 1 breast cancer, 1 hepatocellular cancer, 1 neuroendocrine carcinoma of the lung, 1 rectum carcinoma); 3 sarcomas, 2 cancers of unknown primary (CUP), 1 gastrointestinal stroma tumour (GIST), 1 non Hodgkin lymphoma (NHL). 4: non-AC benign tumours comprised 7 neurogenic tumours, 3 bronchogenic cysts, 3 pheochromocytomas, 3 vascular malformations, 2 haemangiomas, 2 haematomas, 1 myelolipoma, 1 desmoidfibroma, 1 mesenchymal solitary fibrous tumour. Eight patients were excluded from the final analysis: One patient consented but revealed as screen failure as he fulfilled the exclusion criterion of HU <10 and did not receive study-related investigations. Two patients withdrew consent before any study-related investigations were performed; one patient declined further study-related procedures and surgery after the FDG PET result was available; two cases were excluded after completing the study because unenhanced CT tumour attenuation was less than 10 HU upon central imaging analysis; one patient died from heart failure not related to the adrenal tumour before the scheduled adrenalectomy.

A total of 85 patients were recruited (Fig. 1). Due to slow recruitment and the discontinuation of GMP-compliant IMTO deliveries during the COVID19-pandemic, the study ended early. Eight patients were excluded from the final analysis: one screen failure, two withdrew consent before study procedures, one declined further investigations after FDG PET, two had HU <10 at central imaging reading, one died before adrenalectomy, and one declined adrenalectomy as functional imaging indicated a benign non-adrenocortical tumour (follow up imaging showed decreased tumour diameter but he was excluded from final analysis due to missing histopathology) (Fig. 1).

The final analysis cohort comprised 77 patients with available histopathology (Fig. 1, Table 1). All underwent FDG PET imaging, whereas IMTO SPECT imaging was performed in 73 patients. Four patients did not undergo IMTO SPECT due to delivery problems during the pandemic or radiotracer quality control failures.

**Table 1:** Patient characteristics and imaging results from 77 patients with available histopathology.

	All benign (n = 53)	ACA (n = 30)	Non-AC benign (n = 23)	All malignant (n = 24)	ACC (n = 9)	Non-AC malignant (n = 15)
Age (years)	57 0 (33–79) [52·5; 65·0]	57·5 (33–79) [53·0; 65·25]	56 0 (36–79) [49·0; 64·0]	57·5 (30–78) [51·25; 65·0]	53 0 (32–76) [42·0; 72·5]	60·0 (30–78) [52·0; 65·0]
Sex	F 25, M 28	F 17, M 13	F 8, M 15	F 13, M 11	F 7, M 2	F 6, M 9
History of previous malignancies, n^a^	5	4	1	5	1	4
**Adrenal tumour characteristics**						
Max. diameter (cm)^b^	4·5 (2·0–18·1) [3·5; 6·9]	4·4 (2·7–10) [3·7; 5·2]	6·6 (2·0–18·1) [3·4; 9·3]	7·2 (3·4–22) [4·5; 9·9]	7·4 (3·4–14·9) [4·1; 11·2]	6·9 (3·7–22) [4·5; 9·7]
Unenhanced CT attenuation (HU)	25·3 (12–53) [19·0; 32·5]	25·6 (12–42) [19·8; 31·3]	25·0 (13–53) [19·0; 34·0]	33 (20–52) [28·3; 37·5]	36·0 (26–42) [31·0; 37·0]	30·0 (20–52) [28·0; 38·0]
HU > 20, n (%)	36 (68%)	20 (67%)	16 (70%)	23 (96%)	9 (100%)	14 (93%)
Heterogeneous appearance, n (%)	26 (49%)	14 (47%)	12 (52%)	15 (63%)	6 (67%)	9 (60%)
Diameter > 4 cm + (HU > 20 or heterogeneous appearance) n (%)	31 (59%)	16 (53%)	15 (65%)	22 (92%)	8 (89%)	14 (93%)

Data are presented as median (minimum–maximum) [IQR], as indicated. In four patients, tumour size was below 3 cm (2 phaeochromocytomas, 1 ACA and 1 desmoidfibromatosis) but they were operated due to increase in size by at least 1 cm).

F, female; M, male.

a Patients with history of malignancy: ACA category: history of basalioma (n = 1), breast cancer (n = 2), oesophageal cancer (n = 1); non-AC benign tumour category: history for renal cell carcinoma (n = 1); ACC category: history of adenocarcinoma of the lung (n = 1); non-AC malignant tumour category: history of rectal cancer (n = 1), breast cancer (n = 1), hepatocellular carcinoma (n = 1), renal cell carcinoma (n = 1).

b 26 tumours were ≤4 cm in size, of these, four were malignant (2 ACC, 1 metastasis of rectal cancer, 1 GIST).

Histopathology revealed a wide spectrum of differential diagnoses: 53 (69%) were benign and 24 (31%) were malignant, including 30 ACA (39·0%), 9 ACC (11·7%), 23 non-AC benign tumours (29·9%), and 15 non-AC malignant tumours (19·5%) (Fig. 1, Tables 1 and 2; details in Appendix p 7).

**Table 2:** Diagnostic test accuracy of visual FDG-PET or CT imaging as malignancy test, of visual IMTO-SPECT as adrenocortical test, and combined for diagnosing adrenal incidentalomas.

Imaging test			Histopathology (Reference Standard) n (%)				
**FDG-PET classification**							
**IMTO-SPECT classification**			**All**	**ACA**	**Benign non-AC**	**ACC**	**Malignant non-AC**
**Combined classification and tests**			**77 (100)**	**30 (39·0)**	**23 (29·9)**	**9 (11·7)**	**15 (19·5)**
**FDG-PET malignancy**	**n (%)**		77 (100)	30 (100)	23 (100)	9 (100)	15 (100)
+ Positive (5 or 4)	malignant+, benign−		40 (51·9)	13 (43·3)	5 (21·7)	9 (100)	13 (86·7)
○ Indeterminate (3)	indeterminate		3 (3·9)	1 (3·3)	1 (4·3)	0 (0·0)	1 (6·7)
− Negative (2 or 1)	malignant−, benign+		34 (44·2)	16 (53·3)	17 (73·9)	0 (0·0)	1 (6·7)
**IMTO-SPECT AC**	**n (%)**		77 (100)	30 (100)	23 (100)	9 (100)	15 (100)
+ Positive (5 or 4)	AC+		37 (48·1)	27 (90·0)	4 (17·4)	5 (55·6)	1 (6·7)
○ Indeterminate (3)	indeterminate		3 (3·9)	0 (0·0)	3 (13·0)	0 (0·0)	0 (0·0)
− Negative (2 or 1)	AC−		33 (42·9)	1 (3·3)	15 (65·2)	4 (44·4)	13 (86·7)
Missing			4 (5·2)	2 (6·7)	1 (4·3)	0 (0·0)	1 (6·7)
**FDG**	**IMTO**	**Combined Classification n (%)**	77 (100)	30 (100)	23 (100)	9 (100)	15 (100)
+	+	ACC+	19 (24·7)	12 (40·0)	2 (8·7)	5 (55·6)	0 (0·0)
+	○	malignant+, AC indeterminate	0 (0·0)	0 (0·0)	0 (0·0)	0 (0·0)	0 (0·0)
+	–	malignant+, AC−	19 (24·7)	0 (0·0)	3 (13·0)	4 (44·4)	12 (80·0)
+	.	malignant+	2 (2·6)	1 (3·3)	0 (0·0)	0 (0·0)	1 (6·7)
○	+	AC+, malignancy indeterminate	2 (2·6)	1 (3·3)	0 (0·0)	0 (0·0)	1 (6·7)
○	○	both indeterminate	0 (0·0)	0 (0·0)	0 (0·0)	0 (0·0)	0 (0·0)
○	–	AC−, malignancy indeterminate	1 (1·3)	0 (0·0)	1 (4·3)	0 (0·0)	0 (0·0)
○	.	malignancy indeterminate	0 (0·0)	0 (0·0)	0 (0·0)	0 (0·0)	0 (0·0)
−	+	ACA+	16 (20·8)	14 (46·7)	2 (8·7)	0 (0·0)	0 (0·0)
−	○	benign+, AC indeterminate	3 (3·9)	0 (0·0)	3 (13·0)	0 (0·0)	0 (0·0)
−	−	benign+, AC−	13 (16·9)	1 (3·3)	11 (47·8)	0 (0·0)	1 (6·7)
−	.	benign+	2 (2·6)	1 (3·3)	1 (4·3)	0 (0·0)	0 (0·0)
**ACA-test**^a^	**n (%)**		**75 (100)**	**29 (38·7)**	**22 (29·3)**	**9 (12·0)**	**15 (20·0)**
ACA+ (ACA-Test positive)			16 (21·3)	14 (48·3)	2 (9·1)	0 (0·0)	0 (0·0)
PPV (95% CI)	87·5% (61·7%–98·45%)		*16 (100)*	*14 (87·5)*	2 (12·5)	0 (0·0)	0 (0·0)
ACA− (ACA-Test negative)			59 (78·7)	15 (51·7)	20 (90·9)	9 (100)	15 (100)
NPV (95% CI)	74·6% (61·6%–85·0%)		*59 (100)*	15 (25·4)	*20 (33·9)*	*9 (15·3)*	*15 (25·4)*
**Diagnostic test-accuracy**	**(95% CI)**						
Sensitivity	48·3% (29·4%–67·5%)						
Specificity	95·7% (85·2%–99·469%)						
LR (ACA+)	11·1 (3·19–122)						
LR (ACA−)	0·541 (0·350–0·736)						
**ACC-test**^b^	**n (%)**		**75 (100)**	**29 (38·7)**	**23 (30·7)**	**9 (12·0)**	**14 (18·7)**
ACC+ (ACC-Test positive)			21 (28·0)	13 (44·8)	2 (8·7)	5 (55·6)	1 (7·1)
PPV (95% CI)	23·8% (8·2%–47·2%)		*21 (100)*	13 (61·9)	2 (9·5)	*5 (23·8)*	1 (4·8)
ACC− (ACC-Test negative)			54 (72·0)	16 (55·2)	21 (91·3)	4 (44·4)	13 (92·9)
NPV (95% CI)	92·6% (82·1%–97·94%)		*54 (100)*	*16 (29·6)*	*21 (38·9)*	4 (7·4)	*13 (24·1)*
**Diagnostic test-accuracy**	**(95% CI)**						
Sensitivity	55·6% (21·2%–86·3%)						
Specificity	75·8% (63·6%–85·5%)						
LR (ACC+)	2·29 (0·755–4·50)						
LR (ACC−)	0·587 (0·197–1·06)						
**FDG-PET malignancy-test**^c^	**n (%)**		**77 (100)**	**30 (39·0)**	**23 (29·9)**	**9 (11·7)**	**15 (19·5)**
Malignancy+ (FDG Malignancy-Test positive)			43 (55·8)	14 (46·7)	6 (26·1)	9 (100)	14 (93·3)
PPV (95% CI)	53·5% (37·7%–68·8%)		*43 (100)*	14 (32·6)	6 (14·0)	*9 (20·9)*	*14 (32·6)*
Malignancy− (FDG Malignancy-Test negative)			34 (44·2)	16 (53·3)	17 (73·9)	0 (0·0)	1 (6·7)
NPV (95% CI)	97·1% (84·7%–99·926%)		*34 (100)*	*16 (47·1)*	*17 (50·0)*	0 (0·0)	1 (2·9)
**Diagnostic test-accuracy**	**(95% CI)**						
Sensitivity	95·8% (78·9%–99·895%)						
Specificity	62·3% (47·9%–75·2%)						
LR (Malignancy+)	2·54 (1·79–3·82)						
LR (Malignancy−)	0·0669 (0·00213–0·313)						
**IMTO-SPECT AC-test**^d^	**n (%)**		**73 (100)**	**28 (38·4)**	**22 (30·1)**	**9 (12·3)**	**14 (19·2)**
AC+ (IMTO AC-Test positive)			37 (50·7)	27 (96·4)	4 (18·2)	5 (55·6)	1 (7·1)
PPV (95% CI)	86·5% (71·2%–95·46%)		*37 (100)*	*27 (73·0)*	4 (10·8)	*5 (13·5)*	1 (2·7)
AC− (IMTO AC-Test negative)			36 (49·3)	1 (3·6)	18 (81·8)	4 (44·4)	13 (92·9)
NPV (95% CI)	86·1% (70·5%–95·3%)		*36 (100)*	1 (2·8)	*18 (50·0)*	4 (11·1)	*13 (36·1)*
**Diagnostic test-accuracy**	**(95% CI)**						
Sensitivity	86·5% (71·2%–95·46%)						
Specificity	86·1% (70·5%–95·33%)						
LR (AC+)	6·23 (3·01–17·0)						
LR (AC−)	0·157 (0·0575–0·325)						
**Unenhanced CT TA malignancy-test**^e^	**n (%)**		**77 (100)**	**30 (39·0)**	**23 (29·9)**	**9 (11·7)**	**15 (19·5)**
Malignancy+ (CT TA Malignancy-Test positive)			63 (49·4)	23 (76·7)	16 (69·6)	9 (100)	15 (100)
PPV (95% CI)	38·1% (26·1%–51·2%)		*63 (100)*	23 (36·5)	16 (25·4)	*9 (14·3)*	*15 (23·8)*
Malignancy− (CT TA Malignancy-Test negative)			14 (50·6)	7 (23·3)	7 (30·4)	0 (0·0)	0 (0·0)
NPV (95% CI)	100% (76·8%–100%)		*14 (100)*	*7 (50·0)*	*7 (50·0)*	0 (0·0)	0 (0·0)
**Diagnostic test-accuracy**	**(95% CI)**						
Sensitivity	100% (85·8%–100%)						
Specificity	26·4% (15·3%–40·3%)						
LR (Malignancy+)	1·36 (1·15–1·68)						
LR (Malignancy−)	0·0000 (0·0000–0·508)						

n, number of participants; FDG, fluorodesoxyglucose; PET, positron emission tomography; IMTO, iodometomidate; SPECT, single photon emission computed tomography; CT, computed tomography; TA, tumour attenuation; AC, adrenocortical (tissue); ACC, adrenocortical carcinoma; ACA, adrenocortical adenoma; HU, Hounsfield units; CI, confidence interval; CP, Clopper-Pearson CI (1934); BSC, Blyth-Still-Casella CI (1983, 1986); PPV, positive predictive value; NPV, negative predictive value; LR(X), likelihood ratio of test result X.

Based on the histopathology of 77 participants, the estimated pre-test probabilities with two-sided 95% CIs of the four histopathological groups of incidentalomas were for ACA 39·0% (CP 28·0%–50·8%, BSC 28·0%–50·8%), for benign non-AC incidentalomas 29·9% (CP 20·0%–41·4%, BSC 20·5%–40·8%), for ACC 11·7% (CP 5·5%–21·0%, BSC 6·0%–20·5%), and for malignant non-AC incidentalomas 19·5% (CP 11·3%–30·1%, BSC 11·8%–29·2%).

For evaluation of functional imaging (FDG-PET and IMTO-SPECT), results of visual image analysis were used. The visual interpretation criteria included a semi-quantitative scoring system ranging from 1 to 5: Visual scores 1 and 2 indicated no or low tracer uptake, whereas scores 4 and 5 indicate moderate or high tracer uptake. Tumours with intermediate tracer uptake were rated 3 (indeterminate).

In the case that FDG-PET alone already excluded imaging diagnosis of an ACA (n = 2 because of high FDG uptake) or an ACC (n = 2 because of low FDG uptake), results were included in the analysis of the ACA- or ACC-test, respectively, even if no IMTO-SPECT imaging was available.

The n (%) for calculation of the presented predictive values (post-test probabilities) are presented in *italic*.

The results of the cross-sectional analysis for the diagnostic test accuracy (primary and secondary outcome) are presented for the test positive (+) and the test negative (−) outcome by classification groups from histopathology as n (%) and as sensitivity and specificity with exact two-sided 95% CP by classification into the histopathological positive and negative group, respectively. Alternatively, for calculation of predictive values based on assumed pre-test probabilities of the histopathological positive group, the likelihood ratios LR(test+) and LR(test–) are provided, each with exact two-sided 95% CI.

Each two-sided 95% CI is provided as the intersection of two one-sided 97·5% CIs.

a The ACA-Test was defined focussing on high specificity: FDG ○ was also classified as Malignancy+, and IMTO ○ also as AC−.

b The ACC-Test was defined focussing on high sensitivity: FDG ○ was also classified as Malignancy+, and IMTO ○ also as AC+.

c The FDG-PET Malignancy-Test was defined focussing on high sensitivity for malignancy: FDG ○ was also classified as Malignancy+.

d The IMTO-SPECT AC-Test was defined focussing on high specificity for AC origin: IMTO ○ was also classified as AC−.

e The Unenhanced CT TA Malignancy-Test based on the TA measured in HU was defined focussing on very high sensitivity using a cut-off of 20 HU: ≥20 HU was classified as Malignancy+ and <20 HU as Malignancy−.

### Imaging findings

#### Combined FDG/IMTO imaging

**Fig. 2:**
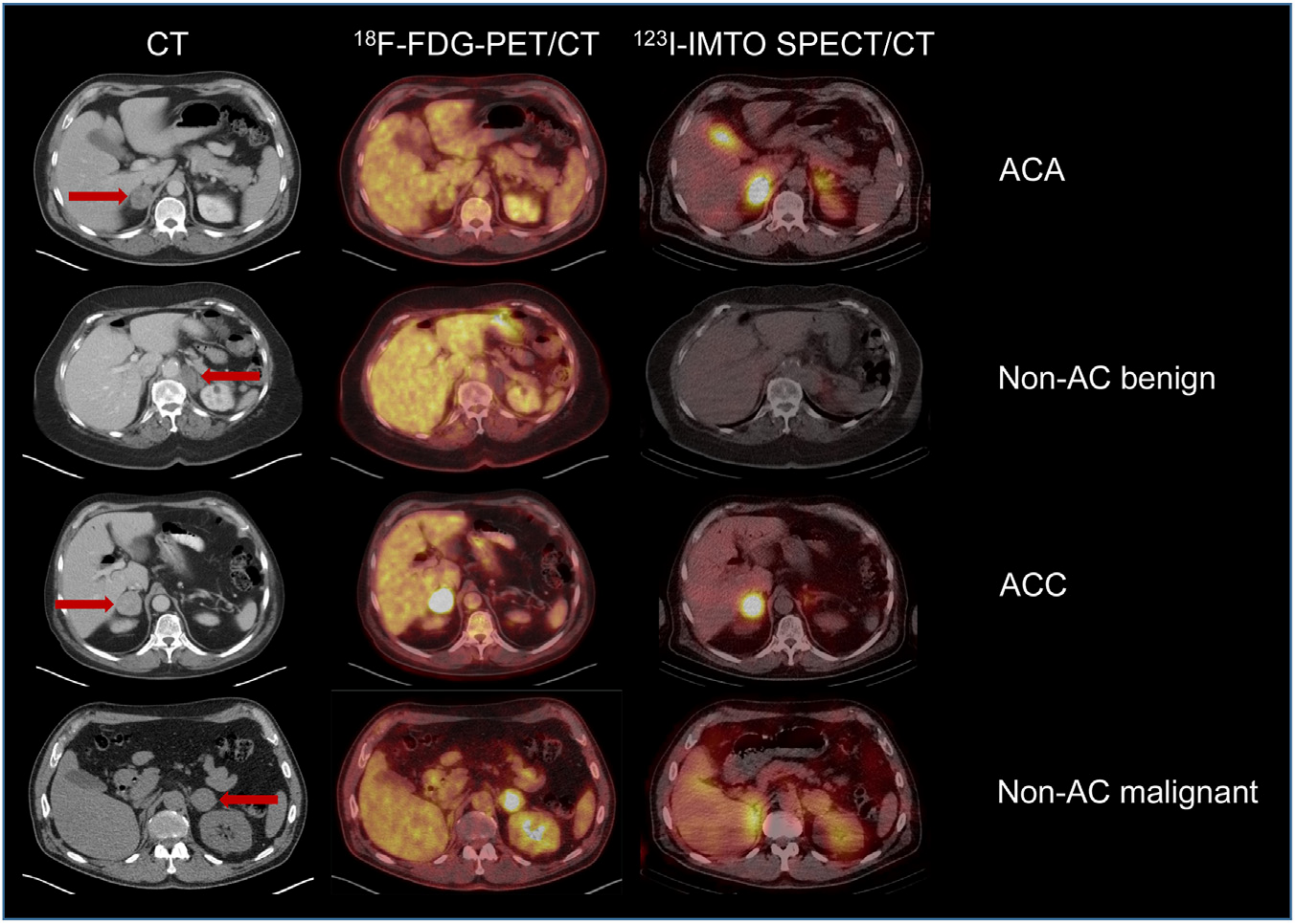
**Illustrative imaging examples for the different tumour categories**. Examples of the categorization into the four tumour subgroups based on the results of functional imaging using FDG PET and IMTO SPECT: ACA: FDG-negative and IMTO-positive; non-AC benign: FDG-negative and IMTO-negative; ACC: FDG-positive and IMTO-positive; non-AC malignant: FDG-positive and IMTO-negative. Left column: unenhanced computed tomography imaging; tumour is indicated by the red arrow.

The primary objective was to classify ACA versus non-ACA by combined FDG/IMTO imaging (FDG-negative/IMTO-positive for ACA) (Fig. 2). If FDG PET alone excluded ACA (due to high FDG uptake, n = 2) or ACC (due to low FDG uptake, n = 2), those results were included in the analysis of the ACA- or ACC-test, even without IMTO imaging.

**Fig. 3:**
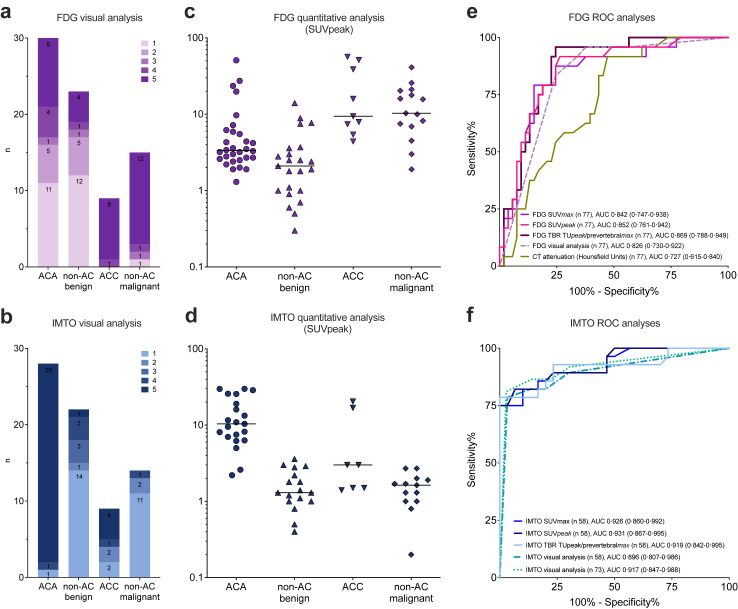
**Results of molecular imaging based on visual and quantitative imaging analysis for the histopathological subcategories**. **a** Visual interpretation of FDG PET in histopathological subcategories with scoring system ranging from 1 to 5. 1 = FDG negative (definitely benign), 2 = mildly FDG avid (more likely benign), 3 = indeterminate (rated as FDG positive), 4 = moderately FDG-avid (more likely malignant), 5 = malignant. **b** Visual interpretation of IMTO SPECT 1 = negative, no adrenocortical origin, 2 = mildly positive, unlikely adrenocortical origin, 3 = indeterminate (rated as IMTO negative), 4 = moderately positive, indicative of adrenocortical origin, 5 = strongly positive, adrenocortical origin. **c** Quantitative analysis of the FDG uptake in the tumour subcategories based on the measurement of SUVpeak (n = 77). Horizontal line = median. **d** Quantitative analysis of the IMTO uptake in the tumour subcategories based on the measurement of SUVpeak (n = 58). Horizontal line = median. **e** ROC analyses for quantitative measures of FDG uptake (SUVmax, SUVpeak and tumour to background ratio TBR adrenal tumour SUVpeak/prevertebral region SUVmax), for visual analysis of FDG uptake using five categories and for attenuation values in unenhanced CT measured in Hounsfield units. Discrimination malignant versus benign tumours. AUCs are reported with 95% CI. **f** ROC analyses for quantitative measures of IMTO uptake in 58 lesions (SUVmax, SUVpeak and tumour to background ratio TBR adrenal tumour SUVpeak/prevertebral region SUVmax), for visual analysis of the 58 lesions with available quantitative measures and for the all lesions with IMTO SPECT (n = 77). Discrimination adrenocortical versus non-adrenocortical tumours. AUCs are reported with 95% CI.

Of 46 non-ACA included in the ACA test analysis, two were incorrectly characterised as ACA based on visual interpretation, resulting in a specificity of 95·7% (95% CI 85·2%–99·47%), although this did not reach statistical significance for >90% specificity (p = 0·15). The positive likelihood ratio was 11·1 (95% CI 3·19–122·2). Among 29 ACA included in the analysis of the ACA-test (28 with combined imaging result), 14 were correctly characterised as ACA based on visual interpretation of combined imaging. Twelve ACA were both IMTO- and FDG-positive and one ACA was IMTO-positive and FDG-indeterminate and misclassified as ACC (Fig. 3a). One ACA with only 30% vital adenoma fractions was both IMTO- and FDG-negative and one ACA with missing IMTO imaging was FDG positive and misclassified as non-ACA. Thus, sensitivity for classification of ACA was only 48·3% (95% CI 29·4%–67·47%).

The secondary objective was classification of ACC versus non-ACC. Of nine ACC, five were correctly characterised as ACC (FDG-positive and IMTO-positive), whereas four were FDG-positive but IMTO-negative, resulting in a sensitivity of 55·6% (95% CI 21·2%–86·3%). Among 66 non-ACC, 13 ACA and 3 non-AC tumours (one malignant, two benign) were misclassified as ACC, resulting in a specificity of 75·8% (95% CI 63·6%–85·5%). Using quantitative image analyses, the test performance for the ACA test, the ACC test, as well as for the malignancy test and the classification as adrenocortical versus non-adrenocortical could be improved. Results and test performance of combined imaging are shown in Table 2, Fig. 3 and in the Appendix p 7, Supplementary Figure S4.

### [^18^F]FDG PET

In visual analysis of all 77 patients, FDG PET revealed high sensitivity (95·8%; 95% CI 78·9%–99·89%) and a high negative predictive value (97·1%; 95% CI 84·7%–99·93%) for classifying malignant versus benign lesions. However, specificity was only 62·3% (95% CI 47·9%–75·2%) (Table 2, Fig. 3a). One low-grade gastrointestinal stroma tumour was misclassified as benign. The low specificity was due to 14 of 30 ACA being categorised as malignant (13 FDG-positive, 1 FDG indeterminate) (Table 2, Fig. 3a). Additionally, one phaeochromocytoma, one haematoma, one neurofibroma, and two schwannomas were FDG-positive, and a benign myelolipoma was indeterminate (examples in Supplementary Figure S5).

Quantitative FDG PET analysis results are presented in Fig. 3c and e, Supplementary Table S3 and Supplementary Figure S7A. Analysis of the 13 FDG-positive and the FDG indeterminate ACA (categorised as malignant) revealed that five FDG-positive ACA were oncocytic tumours. Two FDG-positive ACA were initially diagnosed as ACC by the local pathologist. Comparison of FDG-positive/indeterminate with FDG-negative ACA regarding imaging, demographic and histopathological parameters is shown in Supplementary Table S4.

### [^123^I]Iodometomidate SPECT

In visual analysis of the 73 patients with IMTO SPECT, the sensitivity for classifying adrenocortical versus non-adrenocortical tumours was 86·5% (95% CI 71·2%–95·5%) and specificity was 86·1% (95% CI 70·5%–95·3%) (Table 2). Twenty-seven of 28 ACA and five out of nine ACC were correctly classified as adrenocortical (Fig. 3a, Table 2). False positives included two phaeochromocytomas, one haematoma and one desmoidfibroma among benign non-adrenocortical tumours, and one rectal cancer metastasis among malignant non-adrenocortical tumours (Supplementary Figure S5).

Quantitative imaging for IMTO was available for 58 patients due to equipment and imaging hard-/software differences (Fig. 3d and Supplementary Figure S7B).

### Computed tomography

The maximum tumour diameter was >4 cm in 51 tumours (20 malignant, 31 benign) and ≤4 cm in 26 tumours (4 malignant, 22 benign). Four tumours were <3 cm but included due to a >1 cm growth on follow-up imaging. Malignant lesions were larger with higher HU values compared to benign lesions, although there was a clear overlap (Table 1, Supplementary Figure S6).

All malignant lesions had attenuation values ≥20 HU (range 20–54). Using a cut-off ≥ 20 HU for malignancy, the sensitivity was 100% (95% CI 85·8%–100%). However, 39 of 53 benign lesions also had attenuation values ≥20 HU (20–29 HU (n = 19); ≥30 HU (n = 20)), resulting in a specificity of only 26·4% (95% CI 15·3%–40·3%) (Table 2, Fig. 3f). Compared to FDG-PET, unenhanced CT appears to be of less diagnostic value. ROC curve analysis reveals an AUC of 0·826 (95% CI 0·730–0·922) for visual FDG analysis, compared to 0·727 (95% CI 0·615–0·840) for unenhanced CT, with a difference of 0·099 (95% CI −0·046 to 0·243, p = 0·1811). Quantitative FDG-PET analysis showed a ROC-AUC of 0·842 (95% CI 0·747–0·938) for SUVmax, the most common quantitative measure, and up to 0·869 (95% CI 0·788–0·949) for the target to background ratio (tumour SUVpeak/prevertebral region SUVmax), increasing the difference to 0·142 compared to unenhanced CT (95% CI (0·003–0·280), p = 0·0459) (Fig. 3f), Appendix p 6, Supplementary Figure S7.

### Adverse events

Twentyfour AEs occured in a total of 19 patients, including four grade 3 events in three patients and 20 grade 1 or 2 events in 17 patients. Four AEs in four patients were judged as study-related: pain at the injection site after IMTO injection (n = 3), metallic taste (n = 1), all of them were grade 1 and self-limiting. Seven serious AEs occurred in three patients, including one patient who died from heart failure and sepsis before scheduled adrenalectomy. All SAEs were unrelated to study treatment. Details on AEs are provided in the Appendix p 9.

## Discussion

The FAMIAN study evaluated the diagnostic accuracy of combined FDG/IMTO imaging in classifying ACA. The aim was to develop an ACA-index test based on visual assessments of FDG and IMTO, aiming for high specificity while maintaining adequate sensitivity. This functional imaging approach reached high specificity (95·7%). However, the sensitivity was only 48·3% due to a high number of FDG-positive ACA, leading to their misclassification as ACC. Despite this, utilizing FDG-/IMTO-imaging to guide treatment decisions could have avoided unnecessary surgery in nearly half of the patients with indeterminate lesions that turned out to be ACA on histopathology. This proportion could be increased by using quantitative measurements.

The study group had a lower proportion of ACA (39%) than expected, reducing the pre-test probability boundary from the assumed 50%. However, ACA remained the largest subgroup.^2^^,^^4^^,^^9^^,^^29^ Thus, while excluding malignancy is a primary goal, a method that additionally allows non-invasive entity assignment as ACA can be beneficial. In the FDG-negative tumours, additional IMTO imaging classified ACA with 93% sensitivity and 88% specificity. Importantly, none of the tumours classified as ACA by combined FDG/IMTO imaging were malignant.

The secondary objective of our study was non-invasive classification of ACC. All nine ACC were correctly classified as malignant by FDG PET, but four ACC were IMTO-negative, likely due to tumour dedifferentiation.^28^ Accordingly, combined FDG/IMTO imaging showed limited sensitivity and, due to the large number of ACA falsely classified as ACC, also limited specificity for detection of ACC, making this approach less valuable for preoperative ACC classification.

In terms of differentiating malignant from benign tumours, recent studies suggest raising the cut-off HU value for malignancy suspicion from 10 HU to 20 HU, keeping high sensitivity.^3^^,^^6^^,^^9^ This is affirmed by our study, with all malignant tumours showing HUs ≥20. However, 23 out of 30 ACA (76·7%) had HU ≥20, resulting in a specificity of only 26·4% in our group. It is, however, important to note that tumours with HU <10 were excluded.

In our study, both FDG PET imaging and unenhanced CT imaging were found to have high to very high sensitivity for detection of malignancy, but specificity was low. FDG-PET provided higher specificity compared to unenhanced CT, at a similar sensitivity level. However, improving specificity while maintaining high sensitivity remains a challenge.

Current guidelines suggest stratifying adrenal tumours based on size and the degree of heterogeneity or HU in unenhanced CT imaging. Four categories with different malignancy suspicion levels were defined with corresponding recommendations for further management.^1^ In our study, 53 tumours (69%) fall into the category with the highest suspected malignancy and the recommendation for prompt surgical removal. However, these 53 tumours still included 31 benign masses, including 16 ACA. Of these 31 benign tumours, FDG PET would have correctly classified 18 lesions (58%) as benign. Thus, functional imaging was useful in differentiating between benign and malignant lesions in this specific group, without, however, capturing all benign lesions as such.

Remarkably, about half of ACA in our study showed high FDG uptake, raising questions about their malignant potential. Further analysis revealed that five FDG-positive ACA were oncocytic tumours, known for increased FDG uptake.^30^ Weiss scores were higher in FDG-positive adenomas, and steroid analysis revealed increased androgen production in three FDG-positive ACA, whereas age, sex, tumour size, Ki67 index or unenhanced HU were comparable between FDG-positive and negative ACA. These findings warrant further investigation into the nature of FDG-positive ACA and their need for surgical removal. Clinical follow-up and molecular analyses, such as metabolomics, transcriptomics, and genetic characterisation, comparing FDG-positive tumours with FDG-negative adenomas and ACCs would be of interest.

Our study was limited by the inability to reach the intended number of cases. This clearly reduces statistical power. Nonetheless, to our knowledge the study remains the largest prolective study using innovative molecular imaging approaches in indeterminate adrenal lesions. A major strength is that histopathology served as reference standard in all cases. Our results align well with previous analyses of the individual tracers.^13^, ^14^, ^15^, ^16^, ^17^^,^^19^^,^^23^^,^^24^^,^^27^ Patients with a history of malignancy were not excluded but represented a minority. Of note, 50% of the patients with history of malignancy were diagnosed with a benign adrenal lesion in our study and in cases of malignant adrenal tumours identified, these were not always related to previous extra-adrenal malignancies. Most patients were from the lead centre, Würzburg, a reference centre for adrenal tumours, potentially introducing selection bias. However, the prevalence of adrenocortical lesions was comparable to other prospective studies.^14^^,^^15^^,^^17^

In summary, combined FDG and IMTO imaging non-invasively characterises ACA, the most common tumour entity, with high specificity. Sensitivity is limited due to a notably high number of FDG-positive ACA, an intriguing subgroup deserving further investigation. Current preoperative diagnostic methods, FDG-PET and especially CT, have limitations in distinguishing benign from malignant lesions due to reduced specificity. A significant proportion of tumours can be managed by applying unenhanced HU cut-off values of 10 or 20 HU. Functional imaging with FDG and IMTO helps to further reduce unnecessary surgeries by more effectively classifying adrenal lesions compared to conventional imaging tools. It may be assumed that other CYP11B1-targeting radiotracers would demonstrate comparable applicability to IMTO for the question addressed in this study.^20^, ^21^, ^22^, ^23^

## Contributors

SH, AKB and AS prepared the study protocol and all other study relevant documents. HHM was in charge of the biometric concept and the statistical analysis planning for the study protocol. Statistical analyses were performed by HHM and SH. FB, MM, KM, TP, CTF, CF, AT, KH, HA, MR, MS, and OS were responsible for implementation of the study at the respective centres, for patient recruitment and execution of the study procedures. MF, WS, PH, JW and MR contributed to central data evaluation and visualisation. PH, AKB, MR and SH organised central image evaluation. SK performed additional histopathological evaluation beneath reference pathological examination. MR and SH accessed and verified the underlying data. SH wrote the manuscript together with PH, AS, CTF, WS and WA and all authors edited the report. All authors read and approved the final version of the manuscript.

## Data sharing statement

We will consider sharing deidentified, individual participant-level data that underlie the results reported in this article on receipt of a request detailing the study hypothesis and statistical analysis plan. All requests should be sent to the corresponding author. The corresponding author and lead investigators of this study will discuss all requests and make decisions about whether data sharing is appropriate based on the scientific rigour of the proposal. All applicants will be asked to sign a data access agreement.

## Declaration of interests

SH and AS are inventors on patents for radiotracers targeting CYP11B enzymes for diagnostic evaluation of primary aldosteronism or for theranostics of adrenocortical tumours/carcinoma EP2575899A1, WO/2014/048568 and WO/2018/141541. MM reports grants from AstraZeneca, consulting fees from Novartis, Veraxa and Roche, payments from Telix and GWT-TUD; OS reports funding by the DFG (223465017), grants from Siemens, Life molecular imaging, ABX, honorarium paid to institution from Siemens, advisory board Positrigo (no payments); MF reports grants from Enterome, DSMB Bayer Pharma (payments to institution) and ExCo membership European Network for the Study of Adrenal Tumours and European Society of Endocrinology (unpaid positions), CF reports honoraria and travel support from IPSEN Pharma GmbH, KH reports grants from Novartis, Sofie Biosciences, consulting fees from advanced accelerator applications, Amgen, AstraZeneca, Brain Capital, Bayer, Boston Scientific, Convergent, Curium, Debiopharm, EcoR1, Fusion, GE Healthcare, Immedica, Isotopen Technologien München, Janssen, Merck, Molecular Partners, NVision, POINT Biopharma, Pfizer, Radiopharm Theranostics, Rhine Pharma, Siemens Healthineers, Sofie Biosciences, Telix, and Theragnostics, ymabs, Honorarium from PeerVoice, travel support from Janssen, Data Safety Monitoring Board/advisory board participation for Fusion, GE Healthcare, relationships with Sofie Biosciences, Pharma 15, NVision, Convergent, Aktis Oncology, AdvanCell, JW reports travel support by IPSEN Pharma GmbH, WA reports incoming presidency of European Society of Endocrinology. All other authors declare no competing interests related to the manuscript.

## Appendix A Supplementary data

Appendix

Transfer authorisation

FAM1.7_final_clean

Study group collaborators
