# Supplementary material for: Combined [18F]Fluorodeoxyglucose PET and [123I]Iodometomidate-SPECT for diagnostic evaluation of indeterminate adrenal neoplasias—the cross-sectional diagnostic test accuracy study FAMIAN
Source: eBioMedicine. 2025 May 20;116:105735. doi: 10.1016/j.ebiom.2025.105735 (PMC12148602; doi:10.1016/j.ebiom.2025.105735)
Supplement: Appendix [file mmc1.pdf]

Supplementary Material

"Combined [18F]Fluorodeoxyglucose PET and [123I]Iodometomidate-SPECT for diagnostic evaluation of indeterminate adrenal neoplasias in the FAMIAN study – a prospective diagnostic test accuracy study"

Authors: Stefanie Hahner, Philipp Hartrampf, Felix Beuschlein, Matthias Miederer, Konstanze Miehle, Wiebke Schlötelburg, Carmina Teresa Fuß, Thomas Pfluger, Christian Fottner, Anke Tönjes, Ken Herrmann, Holger Amthauer, Martin Reincke, Mathias Schreckenberger, Osama Sabri, Johanna Werner, Miriam Reuter, Stefan Kircher, Wiebke Arlt, Martin Fassnacht, Andreas Konrad Buck, Hans-Helge Müller, Andreas Schirbel

Further contributors: Christian Furth, Knut Mai, Marcus Quinkler, Frank Weber, Henning Dralle, Benjamin Sandner, Thomas Lincke, Regine Kluge, Nada Rayes, Matthias Weber, Martina Gräsl, Wolfgang Saeger, Joachim Reibetanz

Table of content:

List of contributors **p 2**
Supplementary Material and Methods **p 3**
Detailed Eligibility criteria **p 3**
Study Procedures **p 4**
Radiosynthesis of [123I]Iodometomidate and imaging procedures **p 5**
Imaging with [18F]-FDG and [123I]-IMTO **p 5**
Centralised imaging review **p 5**
Reference Histopathology **p 5**
Outcome measures **p 6**
Data analysis **p 6**
Comparison of ROC curves - DeLong's test for two correlated ROC curves **p 6**
Diagnostic test accuracy of FDG-PET and IMTO-SPECT – comparison of visual and quantitative image analysis **p 7**
Final diagnosis in histopathology **p 7**
Adverse Events/Serious Adverse Events **p 9**
Abbreviations **p 10**
Literature **p 11**
Supplementary Figure 4 Sankey chart-diagnosis based on combined imaging results compared with histopathological findings **p 12**
Supplementary Figure 5 Examples of discordant imaging findings **p 13**
Supplementary Figure 6 Results of conventional imaging (distribution of size and attenuation value) for benign and malignant adrenal masses and FDG-PET imaging results across different malignancy risk categories **p 14**
Supplementary Figure 7 ROC curves for quantitative analysis of FDG PET and IMTO SPECT **p 15**
Supplementary Figure 8 Decision tree **p 17**
Supplementary Table 3 Results of quantitative analysis of functional imaging with FDG or IMTO **p 18**
Supplementary Table 4 Characteristics of FDG-positive versus FDG-negative ACA regarding demographic data, imaging findings and histopathology findings **p 19**

List of Contributors

Germany:
Berlin

- Charité - Universitätsmedizin Berlin, Freie Universität Berlin, Humboldt-Universität zu Berlin, and Berlin Institute of Health, Department of Nuclear Medicine (Holger Amthauer, Christian Furth)
- Charité-Universitätsmedizin Berlin, Corporate member of Freie Universität Berlin, Humboldt-Universität zu Berlin, and Berlin Institute of Health, Department of Endocrinology and Metabolism (Knut Mai)
- Endocrinology in Charlottenburg, Berlin (Marcus Quinkler)

Essen

- Department of Nuclear Medicine, West German Cancer Center (WTZ), University Hospital Essen, University of Duisburg-Essen; German Cancer Consortium (DKTK), Partner Site University Hospital Essen (Ken Herrmann)
- Department of Endocrinology, Diabetology and Metabolism, University of Duisburg-Essen (Dagmar Führer-Sakel)
- Department of General, Visceral and Transplantation Surgery, Division of Endocrine Surgery, University of Duisburg-Essen (Frank Weber, Henning Dralle).

Leipzig

- Medizinische Klinik und Poliklinik III – Endokrinologie, Nephrologie, Rheumatologie, Universitätsklinikum Leipzig (Anke Tönjes, Benjamin Sandner, Konstanze Miehle)
- Klinik und Poliklinik für Nuklearmedizin, Universitätsklinikum Leipzig (Osama Sabri, Thomas Lincke, Regine Kluge)
- Klinik und Poliklinik für Viszeral-, Transplantations-, Thorax- und Gefäßchirurgie, Universitätsklinikum Leipzig (Nada Rayes)

Mainz

- Department of Endocrinology and Metabolism, I Medical Clinic, University Medical Center of the Johannes Gutenberg University Mainz (Matthias Weber, Christian Fottner)
- Department of Nuclear Medicine, University Medical Center of the Johannes Gutenberg University Mainz (M Miederer, M Schreckenberger)

Munich

- Department of Nuclear Medicine, University Hospital, Ludwig-Maximilians-Universität Munich (Thomas Pfluger)
- Medizinische Klinik und Poliklinik IV, Klinikum der Universität, Ludwig-Maximilians-Universität Munich (Martin Reincke, Felix Beuschlein)

Würzburg

- Department of Nuclear Medicine, University Hospital, University of Würzburg. (Philipp Hartrampf, Wiebke Schlötelburg, Andreas K Buck, Andreas Schirbel)
- Department of Internal Medicine I, Division of Endocrinology and Diabetes, University Hospital, University of Würzburg. (Martin Fassnacht, Stefanie Hahner, Johanna Werner, Carmina Teresa Fuss, Miriam Reuter, Martina Gräsl)
- Institute of Pathology, University of Würzburg, Josef-Schneider-Str. 2, 97080 Würzburg, Germany (Stefan Kircher)
- Department of General-, Visceral-, Transplant-, Vascular- and Pediatric Surgery, University Hospital of Wuerzburg, Wuerzburg, Germany (Joachim Reibetanz)
-

Marburg

- Institute of Bioinformatics and Biostatistics, Philipps University of Marburg. (Hans Helge Müller)

Hamburg

- Institute of Pathology, University Medical Center Hamburg-Eppendorf, Hamburg. (Wolfgang Saeger)

Switzerland, Zürich

- Klinik für Endokrinologie, Diabetologie und Klinische Ernährung, UniversitätsSpital Zürich (USZ) und Universität Zürich (UZH)
- The LOOP Zurich - Medical Research Center, Zurich (Felix Beuschlein)

UK, London

- Medical Research Council Laboratory of Medical Sciences, London, UK
- Institute of Clinical Sciences, Faculty of Medicine, Imperial College London, London, UK (Wiebke Arlt)

Supplementary Material and Methods

Detailed Eligibility criteria

**Inclusion criteria**	**Exclusion criteria**
Patients with a solid indeterminate adrenal mass scheduled for surgery (within 3 months) and a diameter > 3 cm or an increase in tumour diameter of > 1 cm in follow-up imaging (imaging data should be no older than 3 months) In unenhanced computerized tomography (CT) the attenuation value of the adrenal tumour is ≥ 10 Hounsfield units (HU).	Diagnosis of pheochromocytoma (diagnosis of pheochromocytoma is based on the clinical decision of an experienced endocrinologist taking into account the results of measurement of plasma metanephrines and normetanephrines OR urinary metanephrines and normetanephrines in combination with further clinical information).
Patients with a solid indeterminate adrenal mass scheduled for surgery (within 3 months) and a diameter > 3 cm or an increase in tumour diameter of > 1 cm in follow-up imaging (imaging data should be no older than 3 months) In unenhanced computerized tomography (CT) the attenuation value of the adrenal tumour is ≥ 10 Hounsfield units (HU).	Diagnosis of primary hyperaldosteronism (diagnosis of hyperaldosteronism is based on the clinical decision taking into account the ratio of plasma or serum aldosterone concentration to plasma renin concentration. In case of elevated aldosterone to renin ratio in combination with arterial hypertension a saline infusion test OR captopril test should be performed to exclude primary aldosteronism).
Age ≥ 30 years	Patient unfit or unwilling to undergo surgery
Written informed consent	Pregnancy or breast feeding
ECOG performance status 0-2	Adrenal Cushing's syndrome confirmed by morning serum cortisol after dexamethasone (1 mg at 23.00 h) > 5 µg/dl (140 nmol/l) AND Plasma ACTH < 5 ng/l AND either urinary free cortisol levels twice the upper limit of normal or bed time (23.00 h) salivary cortisol thrice the upper limit of normal.
Effective contraception in female patients of childbearing potential (defined as all women physiologically capable of becoming pregnant) for minimum of 30 days after IMTO or the next menstrual bleeding – whichever comes first	
Negative pregnancy test in women of childbearing potential (defined as all women physiologically capable of becoming pregnant)	
Ability to comply with the protocol procedures	

Study Procedures
Patients completed four study visits (see Table below). Adverse events (AE) were reported until 30 days after second imaging procedure or surgery – whichever came first.

Visit plan and schedule of assessments for the FAMIAN Study

**Events**	**Screening Period**	**Diagnostic Period**		**Follow-up Period**	
	**Visit 1**	**Visit 2**	**Visit 3**	**Visit 4**	**(Visit 5[e])**
Timepoint	*Day 0*	*≤4 weeks after Visit1*	*≤ 8 weeks after Visit2*	*2-4 weeks after Visit3 or preoperative surgical routine – whichever comes first*	*6-12 Month after Visit3*<br>*Only in case that surgery is not performed*
Informed Consent	X				
Inclusion/Exclusion Criteria	X				
Medical History	X				
Current Medication	X				
Recovery of imaging slides	X				
Routine Blood Tests [a]	X			X	
Review of hormonal assessment according to ENSAT criteria [b]	X				
Pregnancy Test in women of childbearing potential [c]	X	(X)	(X)		
Physical Examination, Height, Weight/BSA	X				
Adverse Event / Serious Adverse Event [d]	X	X	X	X	
24 hour urine for steroid metabolomics	X				
Follow up imaging CT or MRI of adrenal region [e]					(X)
[^{18}F]Flurodeoxyglucose PET/CT		X (if not at visit 3)	X (if not at visit 2)		
[123I]Iodometomidate SPECT/CT		X (if not at visit 3)	X (if not at visit 2)		

a Routine blood tests: Serum-creatinine, urea, glomerular filtration rate (MDRD), blood count (haemoglobin, leukocytes, platelets), aspartate aminotranferase, alanin aminotranferase, alkaline phophatase, and gamma glutamyl transferase.
b Hormonal assessment: Morning serum cortisol after 1 mg dexamethasone 23:00, (urinary free cortisol), (midnight salivary cortisol), baseline plasma ACTH, plasma renin concentration or plasma renin activity, serum aldosterone, (DHEAS), (17-OH-progesterone) , plasma metanephrines/plasma normetanephrines or urinary metanephrine/normetanephrine excretion
c At the discretion of investigator following usual care at the respective clinic of nuclear medicine.
d Adverse events (AEs) collected from inclusion of study to visit 4.
e Only in case of refusal of surgery, follow up imaging (Computed tomography, CT or magnetic resonance imaging, MRI) 6-12 months after inclusion into the study and at the discretion of the attending physician

Radiosynthesis of [^{123}I]Iodometomidate and imaging procedures
Labelling of [^{123}I]Iodometomidate for in-house-production was performed by the study centre Würzburg holding a manufacturing authorization for this tracer as previously described [1]. The remaining study centres were provided with [^{123}I]Iodometomidate by the company MAP Medical Technologies Oy (Elementtitie 27, FI-41160 Tikkakoski, Finnland).

Imaging with [^{18}F]-FDG and [^{123}I]-IMTO
All participating centres used state-of-the-art hybrid PET/CT devices and gamma cameras with hybrid SPECT/CT. A median activity of 290 MBq (230-507 MBq) FDG were injected i.v.. Whole-body PET/CT scans were performed after one hour, covering from vertex to upper thighs. Within the protocol only low dose CT was performed if not otherwise needed. CT data were also used for attenuation correction and anatomic localization of PET lesions. Thirty minutes before injection of the radiotracer, radioiodine uptake into the thyroid gland was blocked by oral administration of 600-1200 mg sodium perchlorate. Thyroid blocking was continued for 1-3 days.
Thirty minutes before injection of a median acitivity of 170 MBq (76-203 MBq) ^{123}I-IMTO, radioiodine uptake into the thyroid gland was blocked by oral administration of sodium perchlorate. Planar scans of the whole body were acquired 4 h post injection using a standard technique. All patients also received SPECT/CT imaging between 4 and 6 hours post injection.

Centralised imaging review
A positive lesion was defined as focal or diffuse uptake in the adrenal tumour higher than surrounding background activity. The visual interpretation criteria included a semi-quantitative scoring system ranging from 1 to 5:

Visual score	FDG	IMTO
**1**	FDG negative, definitely benign	IMTO negative, no adrenocortical origin
**2**	mildly FDG-avid/ more likely benign	mildly positive, unlikely adrenocortical origin
**3**	Intermediate FDG uptake, indeterminate with regard to dignity	Intermediate IMTO uptake, indeterminate with regard to entity
**4**	moderately FDG-avid/more likely malignant as indeterminate	moderately IMTO positive, indicative for adrenocortical origin
**5**	High FDG uptake, malignant	Strongly positive, adrenocortical origin

Quantitative analyses for tracer accumulation (FDG and IMTO) was performed by placing a volume-of-interest (VOI) around the adrenal mass and the contralateral unaffected gland and included SUVmax and SUVpeak. HU of the adrenal tumour and the contralateral unaffected gland were determined by placing a region-of-interest (ROI). For the reference regions, a 15 mm VOI in the descending aorta in the prevertebral region and a 40 mm VOI in the unaffected liver were placed and SUVmax, SUVmean and SUVpeak were determined. Tumour-to background-ratios (TBR) were then calculated for SUVmax and SUVpeak in the adrenal tumour compared to SUVmax, SUVpeak and SUVmean of the liver and the aorta in the prevertebral region. Quantification for IMTO SPECT was only available in 58 patients due to technical reasons.
The classification as homogeneous or heterogeneous in CT imaging was based on the visual image impression.

Reference Histopathology
Analysis by the reference pathology followed a strict algorithm: The best suited tissue block was used for further specialized stainings: PAS, MG and immunohistologial analysis. Pancreatin KL1, synaptophysin, chromogranin A, steroidogenic factor 1, melan A, and Ki67 immunohistochemistry was performed. In case of an assumed pheochromocytoma in addition S100-protein and p53 immunohistochemistry was be performed. In case of suspected metastasis the following cytokeratin markers were used: CK 5, CK 7, CK 20 and further tissue markers as needed (e. g. TTF-1, CDX-2 or RCC, in case of suspected sarcoma smooth muscle actin, desmin and vimentin, in case of suspected melanoma metastasis Hmb-45). After immunohistological differentiation in cortical, medullary and metastatic lesions the adrenal tumour was classified. Differentiation of benign and malignant lesions: in case of an adrenocortical tumour the Weiss score was used[2]. In case of ongoing uncertainty, the additional scoring systems of van Slooten et al. and Hough et al. were applied[3,4]. In case of a non-secretory

phaeochromocytoma, assessment of malignancy was based on the scoring systems of Kimura et al. and Thomson et al.[5,6]

Outcome measures
Primary efficacy endpoint: Classification ACA/non-ACA and diagnostic test result ACA+/ACA- for specificity (rate estimation) of the diagnostic ACA test and for the likelihood ratio of a positive diagnostic test (using rate estimation of the sensitivity).
Key secondary endpoints were: Sensitivity of the diagnostic ACA test and likelihood ratio of a negative diagnostic ACA test, classification of indeterminate adrenal neoplasias for a priori rates of ACA, ACC and other benign as well as malignant adrenal tumours. Detection rates similar to sensitivity and specificity for identifying ACC and tumours other than ACA and ACC using combined FDG- and IMTO- imaging. Assessment of safety.

Data analysis
Tumours classified as "indeterminate" (score 3) on visual analysis of FDG-PET were assigned to the category of FDG-positive (malignant) tumours, while tumours classified as "indeterminate" on visual analysis of IMTO-SPECT were assigned to the category of IMTO-negative (non-adrenocortical) tumours. Accordingly, based on the pathohistological results, the tumours were assigned to one of the four groups (ACA, ACC, non-AC benign, non-AC malignant).
The diagnostic measures of primary interest were sensitivity, specificity, and likelihood ratio of a positive diagnostic ACA test (IMTO positive and FDG negative). The primary clinical outcome aimed to achieve a correct non-invasive diagnosis using the combined ACA test of histologically confirmed adrenocortical adenoma and neoplasia distinct from adrenocortical adenoma (for the estimation of the sensitivity and the specificity of the combined ACA test).
Tumour to background ratios (TBR) of standardized uptake values (SUV) between the adrenal tumours and the liver or the aorta in the prevertebral region were calculated. Receiver operating characteristic (ROC) curves and AUC-ROCs were calculated for SUVs.
Receiver operating characteristic (ROC) curves and AUC-ROCs were calculated for quantitative data of both visual analysis and quantitative parameters. These ROC curves were then compared, with the AUC serving as a measure of the discriminatory ability of quantitative or ordinal scores. We specifically investigated whether quantitative analyses outperform visual analysis within the functional imaging methods, and we compared FDG-PET diagnostics with CT using the DeLong's test for two correlated ROC curves. Comparisons between groups were performed by using Mann-Whitney U test for continuous variables.
Quantitative data are presented as mean±SD or median and min/max. Categorial variables are presented as numbers (percentages). Test performance metrics (sensitivity, specificity, PPV, NPV, accuracy and positive as well as negative likelihood ratio) are expressed as percentages and provided with their 95% confidence intervals. Specificity and sensitivity of the ACA test were estimated using the method of Clopper and Pearson.

Supplementary Results

DeLong's test for two correlated ROC curves
To answer the question of whether quantitative analysis has an advantage over visual image analysis and also to what extent FDG-PET diagnostics compares with the determination of Hounsfield units in CT with regard to malignancy assessment, comparisons of the ROC analyses were carried out using the DeLong method. The AUCs in the quantitative analysis were slightly higher than in the visual analysis and with regard to the FDG-PET diagnostics, there appears to be an advantage of the quantitative analysis over the visual analysis, more so than in the IMTO diagnostics. With regard to malignancy assessment by FDG or CT, quantitative FDG image analysis showed superiority over the determination of unenhanced HU in CT.

FDG (malignant versus benign), n=77, AUC differences with 95% CI:

		AUC differences with 95% CI	
	**AUC**	**Visual analysis**	**CT (HU)**
**SUVmax**	0·842	0·0165 (-0·0355 to 0·0686), p=0·53	0·1152 (-0·0422 to 0·2726), p=0·15
**SUVpeak**	0·852	0·0259 (-0·0190 to 0·0709), p=0·26	0·1246 (-0·0258 to 0·2750), p=0·10
**TBRpeak/livermax**	0·861	0·0354 (-0·0113to 0·0821), p=0·14	0·1340 (-0·0079 to 0·2760), p=0·064
**TBRpeak/prev·max**	0·869	0·0428 (-0·0048to 0·0905),p=0·078	0·1415 (0·0026 to 0·2804), p=0·046
**Visual analysis**	0·826	-	-0·0987 (-0·2433 to 0·0459), p=0·181
**CT (HU)**	0·727	0·0987 (-0·0459-0·2433), p=0·18	-

IMTO (adrenocortical versus non-adrenocortical), n=58, AUC differences with 95% CI:

		AUC differences with 95% CI
	**AUC**	**Visual analysis**
**SUVmax**	0·9262	0·01651 (-0·02002 to 0·07954), p=0·24
**SUVpeak**	0·9310	0·02594 (-0·01834 to 0·08739), p=0·20
**Visual analysis**	0·8964	-

Diagnostic test accuracy of FDG-PET and IMTO-SPECT – comparison of visual and quantitative image analysis

Quantitative image interpretation showed improved results compared to visual interpretation when applied to the 58 tumours in the study cohort (21 ACA, 7 ACC, 17 non-AC benign, and 13 non-AC malignant).
For visual interpretation of IMTO SPECT (n=58), sensitivity was 82 1% (95%CI 63 1 to 93 9) and specificity 86 7% (69 3 to 96 2). For IMTO SUVpeak, at the same level of sensitivity of 82 1%, specificity was increased to 93 3% (77 9 to 99 2) at a cut-off of 2 95 for IMTO SUVpeak.
Similarly, for FDG PET (n=77), when using a cut off at 2 for visual interpretation, sensitivity was 95 8% (78 9 to 99 9) with specificity at 62 3% (47 9% to 75 2%). For FDG TBR adrenal tumorSUVpeak/prevertebral regionSUVmax at the same level of sensitivity of 95 8%, specificity was increased to 75 5% (61 7 to 86 2) at a cut-off of 1 65.

When using the above determined cut-offs for IMTO SUVpeak (2 95) and for FDG TBR adrenal tumorSUVpeak/prevertebral regionSUVmax (1 65), 41 of 58 tumours were correctly characterized according to the four categories (ACA, ACC, non-AC benign and non-AC malignant) in comparison to 34 tumours correctly characterized by visual interpretation.

With regard to the ACA test and the ACC test in the 58 patients with complete quantitative image analyses, the following test accuracy values were determined, indicating that quantitative assessment improves test accuracy:

		Sensitivity (95% CI)	Specificity (95% CI)	PPV (95% CI)	NPV (95% CI)
ACA test	quantitative	61 9% (38 4 to 81 9)	97 3% (85 8 to 99 9)	92 9% (64 6 to 98 9)	81 8% (72 2 to 88 6)
	visual	47 6% (25 7 to 70 2)	97 3% (85 8 to 99 9)	90 9% (57 9 to 98 6)	76 6% (68 5 to 83 2)
ACC test	quantitative	57 1% (18 4 to 90 1)	86 3% (73 7 to 94 3)	36 4% (18 2 to 59 4)	93 6% (86 1 to 97 2)
	visual	42 9% (9 9 to 81 5)	74 5 (60 4 to 85 7)	18 8% (8 0 to 38 0)	90 5% (83 1 to 94 9)

Final diagnosis in histopathology
Histopathology was available from 77 patients (surgical specimens in 72 patients, tumour biopsies from five patients).
Underlying diagnoses of the tumours undergoing biopsy was: cancer of unknown primary (CUP), chorion carcinoma, Non-Hodgkin Lymphoma, sarcoma, large-cell neuroendocrine carcinoma of the lung.
Three patients presented with bilateral lesions: bilateral ACA (n=1), CUP (n=1) and metastasis of chorion carcinoma (n=1).

There were 30 **adrenocortical adenomas (ACA)** representative of 39% of the overall cohort. Four ACA were mixed tumours, with an ACA and a myelolipoma component.

The diagnostic spectrum in the 23 **non-adrenocortical (non-AC) benign tumours** (30% of the overall cohort) comprised the following:

- phaeochromocytoma (n=3)
- myelolipoma (n=1)
- ganglioneuroma (n=2)
- ganglioneuroma-pheochromocytoma composite tumour (n=1)
- schwannoma (n=3)

- neurofibroma (n=1)
- desmoid fibroma (n=1)
- mesenchymal solitary fibrous tumour (n=1)
- vascular malformation (n=3)
- haemangiomas (n=2)
- haematoma (n=2)
- bronchogenic cyst (n=3)

Desmoid fibromas are not cancers but often behave aggressively and can grow extensive and invasively; for the purposes of the FAMIAN study we classified this tumour as benign.

Nine tumours (11.5% of the overall cohort) were diagnosed as **adrenocortical carcinoma (ACC)**.

The **non-adrenocortical (Non-AC) malignant tumours** (n=15; 19.5% of the overall cohort) comprised:

- metastases of an extra-adrenal primary cancer (n=8; 2 melanomas, 1 chorion carcinoma, 1 renal cell carcinoma, 1 breast cancer, 1 hepatocellular carcinoma, 1 neuroendocrine carcinoma of the lung, 1 rectal cancer)
- sarcomas (n=3)
- cancers of unknown primary (CUP; n=2)
- gastrointestinal stroma tumour (GIST; n=1)
- Non-Hodgkin Lymphoma (NHL; n=1)

A **history of malignancy** was documented in five patients with benign adrenal lesions and in five patients with malignant adrenal lesions; of the latter, four were diagnosed with an adrenal metastasis of their known extra-adrenal primary cancer and one female patient with breast cancer was newly diagnosed with ACC. Individuals in whom an adrenal lesion was detected during routine follow-up of a previously known malignant disease were not explicitly excluded from the FAMIAN study if surgical removal of the tumour was considered appropriate. Of the 10 included patients with a history of malignancy, the exact reason for imaging was not documented in two patients. In three participants the lesion was detected during routine oncological follow-up imaging, in one patient imaging was performed 23 years after diagnosis of breast cancer for suspicion of relapse, in four patients imaging was performed for non-oncological reasons.

Adverse Events

Pat No	Description	CTC	Outcome	Causality	Action taken for AE
1	Hypokalemia	1	recovered	not related	none
2	Arterial Hypertension	3	ongoing	not related	concomitant medication
2	Conduction disorder (RBBB - ECG finding)	1	ongoing	not related	none
3	Fever, increasing signs of infection	3	recovered/resolved	not related	concomitant medication
3	Seizures (3x postoperative)	3	recovered/resolved	not related	concomitant medication
3	Anemia	2	recovered/resolved	not related	none
3	Hypokalemia	2	recovered/resolved	not related	concomitant medication
4	Rash, maculo-papular	1	recovered/resolved	not related	none
5	Headache	1	recovered/resolved	not related	concomitant medication
5	Dysesthesia	1	not available	not related	none
6	Conjunctivitis	1	recovered	not related	concomitant medication
7	Hypertension	2	ongoing	not related	concomitant medication
7	Fever	1	recovered	not related	concomitant medication
8	Diarrhea	1	recovered	not related	none
9	Inflammation at injection site	1	recovered	not related	none
10	Infection, unclear origin	3	recovered	not related	Concomitant medication
11	Rhinitis infectiva	2	recovered	not related	Concomitant medication
12	Flatulence	1	not recovered	not related	medication
13	Diarrhea	1	ongoing at EOS	not related	concomitant medication
14	Diarrhea	1	recovered	not related	none
15	Salivary duct inflammation (metallic taste)	1	recovered	possibly related	none
16	Pain at injection site	1	recovered/resolved	related	non-drug therapy
17	Pain at injection site	1	recovered	related	none
18	Pain at injection site	1	recovered	related	none

Serious Adverse Events

Pat No	Description	CTC	Outcome	Causality	Action taken for AE
3	Ileus (paralytic)	4	recovered with sequelae	not related	re-laparotomy, anus praeter
3	Re-Laparotomy, anastomotic leak after hemicolectomy	4	recovered with sequelae	not related	re-laparotomy, anus praeter
10	Seizures	3	recovered	not related	Concomitant medication
10	Hypertensive Crisis	3	recovered	not related	Concomitant medication
19	Heart failure	5	fatal	not related	hospitalisation
19	Haematothorax	4	recovered	not related	surgery
19	Sepsis	3	recovered	not related	antibiotics

The SAE report included three patients:

1. Patient 1: A 70-year-old man scheduled for elective adrenalectomy due to a 3 cm adrenal neoplasia and hemicolectomy for suspected colorectal carcinoma. He was enrolled in the FAMIAN study for diagnostic evaluation of his adrenal lesion. Due to comorbidities and the anticipated complexity of the surgery, the adrenalectomy was postponed, and only the hemicolectomy was performed. Six days after the hemicolectomy, the patient exhibited ileus symptoms and developed a fever, leading to a re-laparotomy 10 days after the initial surgery. An anastomotic leak was detected, and a colostomy was created. The patient, known to have symptomatic epilepsy

due to neurosarcoidosis, experienced three generalised seizures after the second surgery. Both the investigator and the sponsor assessed these events as unrelated to the study drug and procedures.

2. Patient 2: A 70-year-old man with bilateral adrenal masses scheduled for adrenalectomy. He experienced seizures and a hypertensive crisis requiring ICU transfer hours after receiving 123I-Iodometomidate. A neurological expert attributed the seizures to paraneoplastic phenomena, supported by a histological diagnosis of cancer of unknown primary. He received permanent anticonvulsive treatment, and the event was classified as unrelated to the study drug.

3. Patient 3: A 71-year-old man with multiple conditions, including coronary heart disease, peripheral artery disease, orthostatic syncope, and COPD. He was part of the FAMIAN trial for an unclear adrenal tumour. During the follow-up period, before the scheduled adrenalectomy and two weeks after receiving the investigational drug, he experienced worsening heart failure and was hospitalised. He was diagnosed with pleural effusion, treated with thoracocentesis, which led to a haemothorax requiring thoracic surgery. Subsequently, he developed sepsis and died from heart failure. This event was also classified as unrelated to the study drug.

Abbreviations

ACA	adrenocortical adenoma
ACC	adrenocortical carcinoma
ACTH	adrenocorticotropic hormone
AE	adverse event
AUC	area under the curve
CI	confidence interval
CT	computed tomography
CUP	cancers of unknown primary
CYP	cytochrome P450 enzyme
ENSAT	European Network for the Study of Adrenal Tumours (www.ensat.org)
FAMIAN	Combined 18F-Fluorodeoxyglucose (FDG) Positron Emission Tomography (PET) and Metomidate Imaging for Adrenal Neoplasia (FAMIAN-Study) - a Diagnostic Study
FDG	fluorodeoxyglucose
GIST	gastrointestinal stroma tumour
HU	hounsfield units
IMTO	iodometomidate
LR	likelihood ratio
NHL	non Hodgkin lymphoma
non-AC	non-adrenocortical
NPV	negative predictive value
PET	positron emission tomography
PPV	positive predictive value
ROC	receiver operating characteristic
SPECT	single photon emission computed tomography
SUV	standardized uptake value
TA	tumour attenuation
VOI	volume of interest

Literature

1. Kreissl MC, Schirbel A, Fassnacht M, et al. [(1)(2)(3)I]Iodometomidate imaging in adrenocortical carcinoma. *J Clin Endocrinol Metab* 2013; **98**(7): 2755-64.
2. Weiss LM, Medeiros LJ, Vickery AL, Jr. Pathologic features of prognostic significance in adrenocortical carcinoma. *Am J Surg Pathol* 1989; **13**(3): 202-6.
3. van Slooten H, Schaberg A, Smeenk D, Moolenaar AJ. Morphologic characteristics of benign and malignant adrenocortical tumors. *Cancer* 1985; **55**(4): 766-73.
4. Hough AJ, Hollifield JW, Page DL, Hartmann WH. Prognostic factors in adrenal cortical tumors. A mathematical analysis of clinical and morphologic data. *Am J Clin Pathol* 1979; **72**(3): 390-9.
5. Kimura N, Watanabe T, Noshiro T, Shizawa S, Miura Y. Histological grading of adrenal and extra-adrenal pheochromocytomas and relationship to prognosis: a clinicopathological analysis of 116 adrenal pheochromocytomas and 30 extra-adrenal sympathetic paragangliomas including 38 malignant tumors. *Endocr Pathol* 2005; **16**(1): 23-32.
6. Thompson LD. Pheochromocytoma of the Adrenal gland Scaled Score (PASS) to separate benign from malignant neoplasms: a clinicopathologic and immunophenotypic study of 100 cases. *Am J Surg Pathol* 2002; **26**(5): 551-66.
7. DeLong, Elizabeth R., David M. DeLong, and Daniel L. Clarke-Pearson. "Comparing the areas under two or more correlated receiver operating characteristic curves: a nonparametric approach." Biometrics (1988): 837-845.

Supplementary Figures

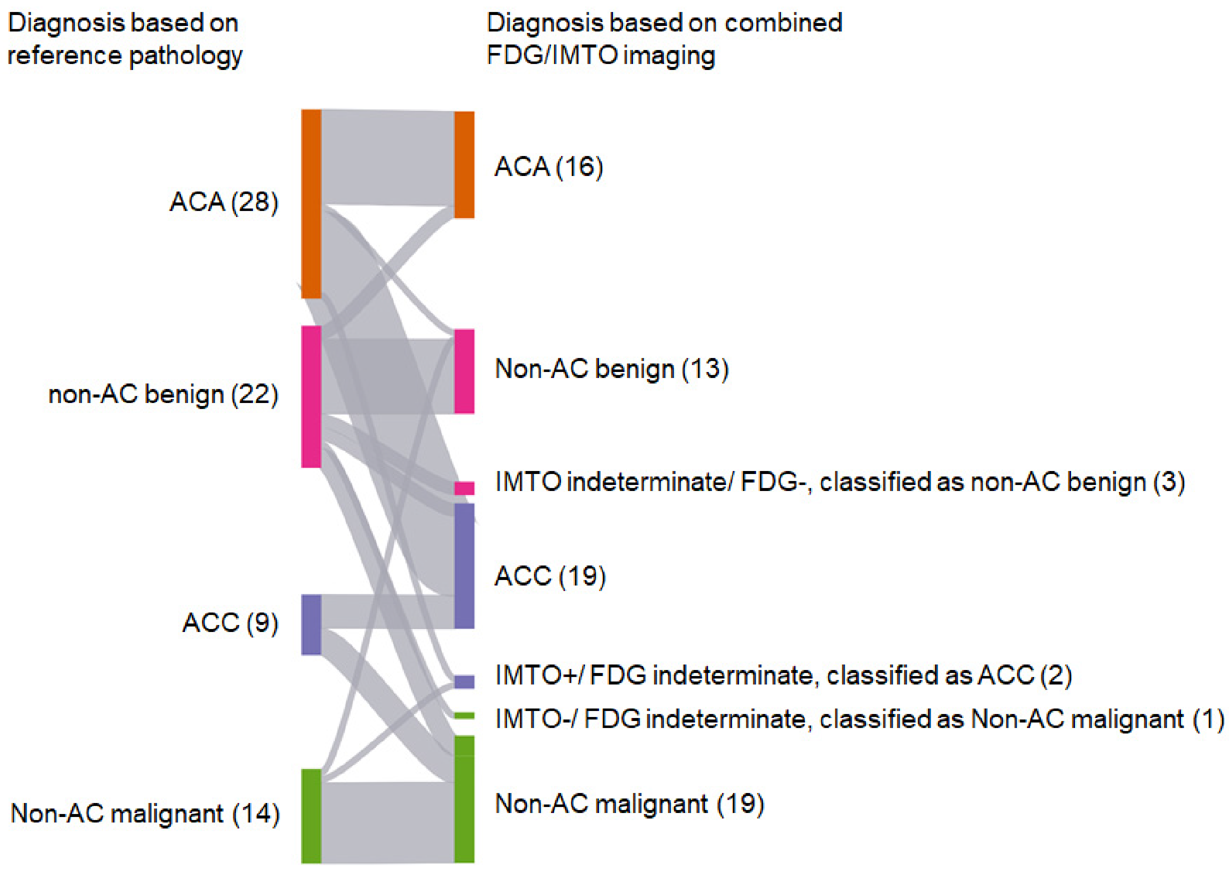

Supplementary Figure 4 Comparison of the diagnosis based on combined imaging results with histopathological findings.

Sankey diagram, comparison of tumour categorization by combined imaging and by histopathology in 73 study participants in whom both IMTO- and FDG-imaging and histopathology was available. All imaging results were based on visual image interpretation of functional imaging. The visual interpretation criteria included a semi-quantitative scoring system ranging from 1 to 5:

Visual scores 1 and 2 indicated no or low tracer uptake, whereas scores 4 and 5 indicate moderate or high tracer uptake. Tumours with intermediate tracer uptake were rated 3 (indeterminate) (see Appendix page 4). If tumours were classified as 3 (indeterminate) on IMTO imaging, they were determined to belong to the group of non-adrenocortical tumours. Tumours classified as 3 on FDG PET were assigned to malignant tumours.

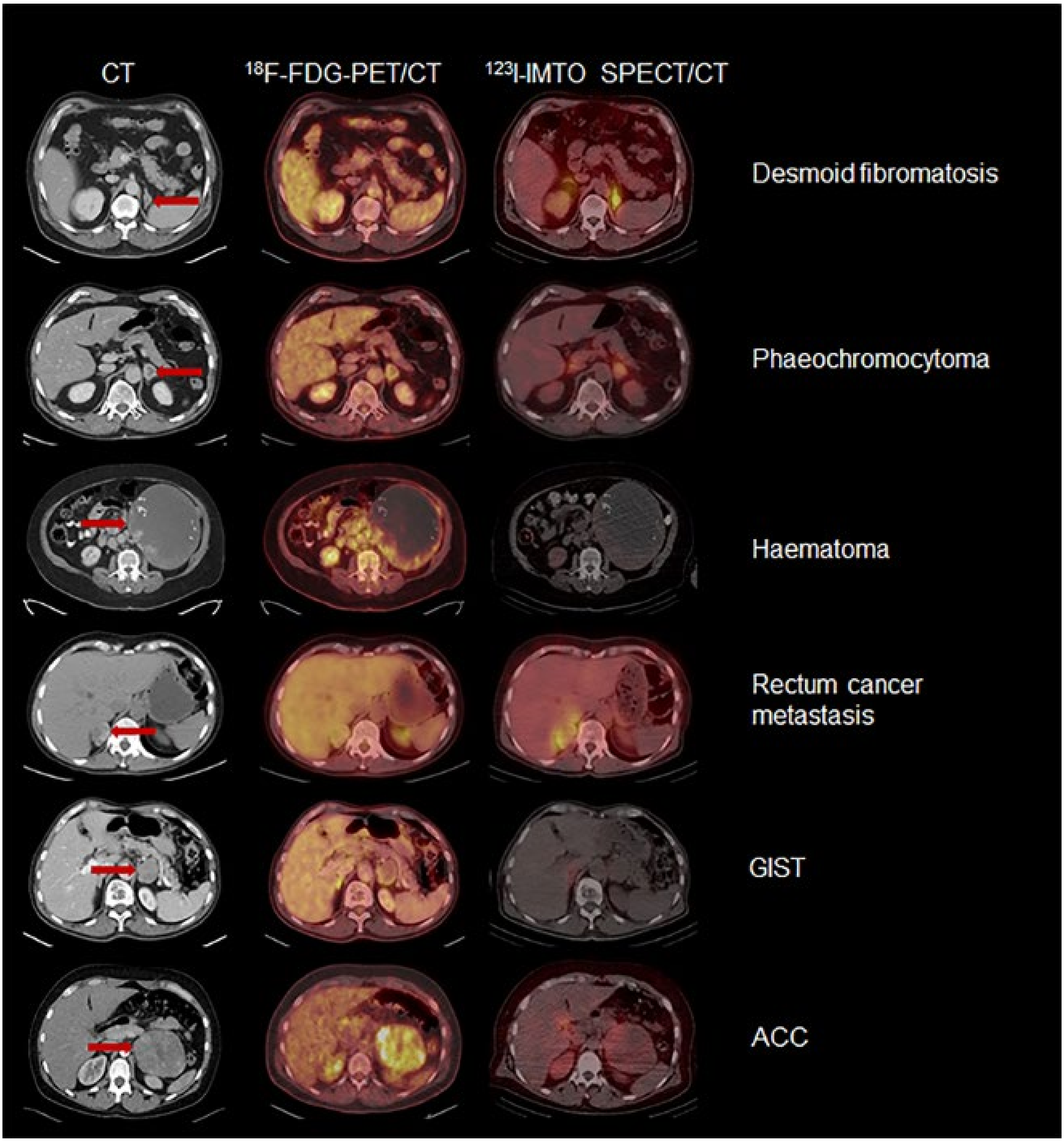

Supplementary Figure 5 Imaging findings (computed tomography, FDG PET and IMTO SPECT) of study participants with functional imaging results that were discordant with the histopathological findings

The adrenal tumour is indicated by a red arrow.

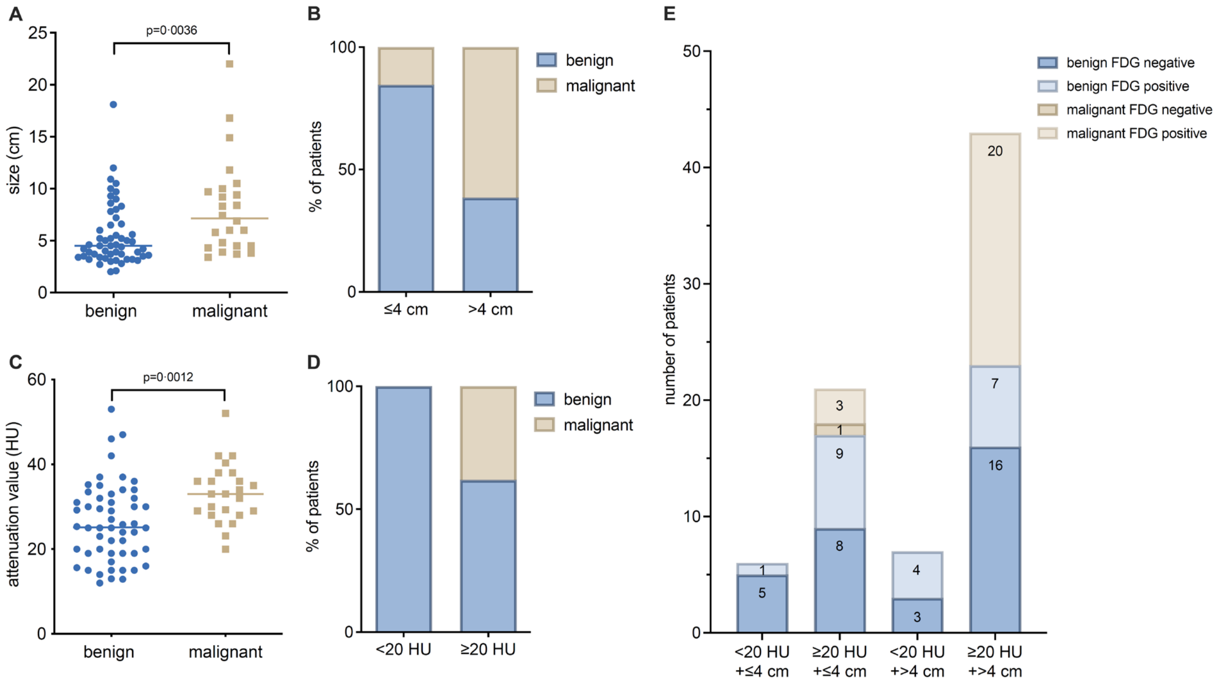

Supplementary Figure 6 Results of conventional imaging (distribution of size and attenuation value) for benign and malignant adrenal masses and FDG-PET imaging results across different malignancy risk categories

A Distribution of tumour diameter in benign and malignant lesions. Horizontal line: median. B Proportion of benign and malignant tumours for tumours ≤4 cm or > 4cm, C Distribution of attenuation value in unenhanced CT in benign and malignant lesions, D Proportion of benign and malignant tumours for tumours <20 HU or ≥20 HU. E FDG-PET imaging results (visual analysis) and histopathological classification as benign or malignant across different malignancy risk categories.

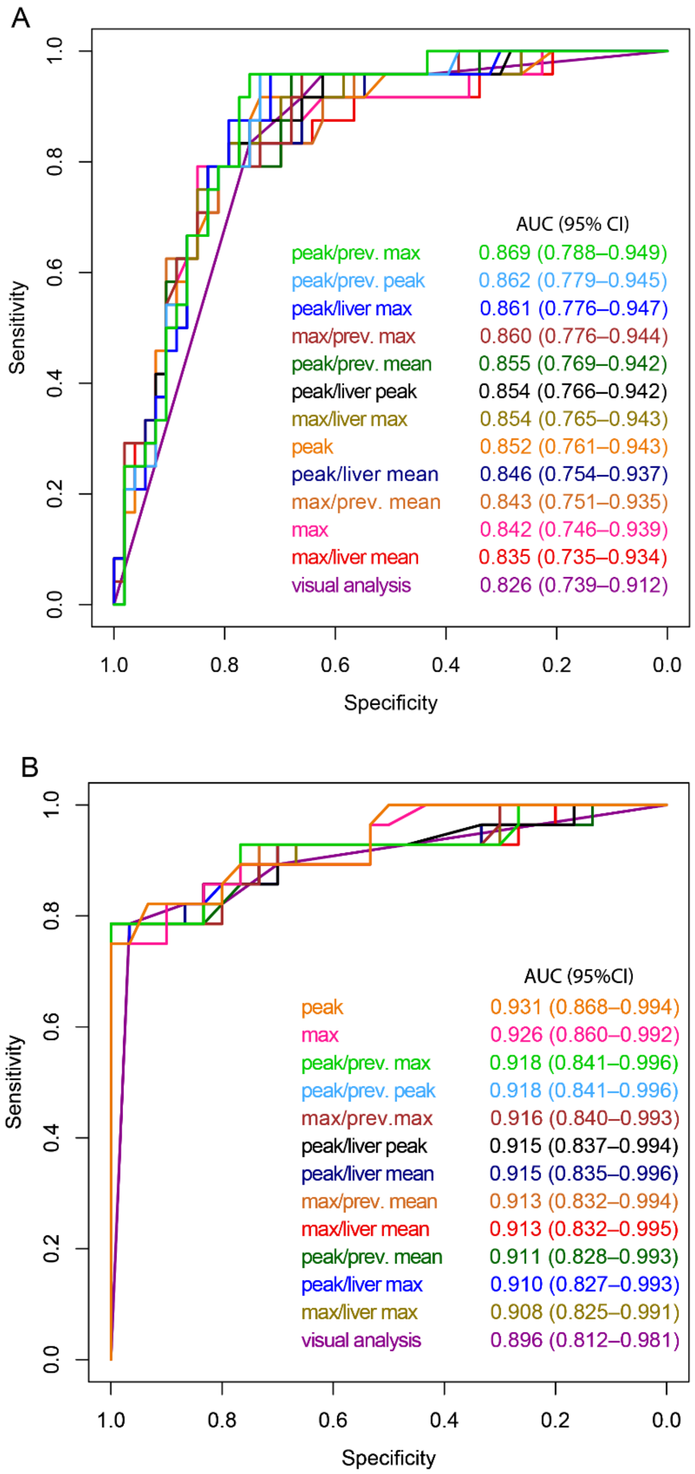

Supplementary Figure 7 ROC curves for quantitative analysis and visual analysis of FDG PET (n=77) and of IMTO SPECT (n=58)

A ROC analyses for FDG PET, **B** ROC analyses for IMTO SPECT. ROC curves and AUC values with 95% confidence intervals are shown for the visual analysis and for determination of SUV*max*, and SUV*peak* of adrenal tumours. Additionally, ROC curves and AUC values are provided for the adrenal tumor-to-background ratios, using SUV*max*, SUV*peak* and SUV*mean* from the liver and the prevertebral region as reference backgrounds. Comparison of ROC curves according to DeLong's test for correlated ROC curves, see p 7 of this Appendix.

For FDG, the highest AUC was observed for TBR TUpeak/prevertebralmax. At a cut-off ratio of >1.64, a sensitivity of 95.8% (95% CI: 78.9 to 99.9) and a specificity of 75.5% (95% CI: 61.7 to 86.2) were achieved. When sensitivity was set to 100% (95% CI: 85.8 to 100), specificity was 43.4% (95% CI: 29.8 to 57.7) at a cut-off >1.05.

For IMTO, the highest AUC was observed for SUVpeak. At a cut-off value of >3.3, sensitivity was 75% (95% CI: 55.1 to 89.3) and specificity was 96.7% (95% CI: 82.8 to 99.9). When specificity was set to 100% (95% CI: 88.4 to 100), sensitivity for adrenocortical lesions was 75% (95% CI: 55.1 to 89.3) at a cut-off of >4.3.

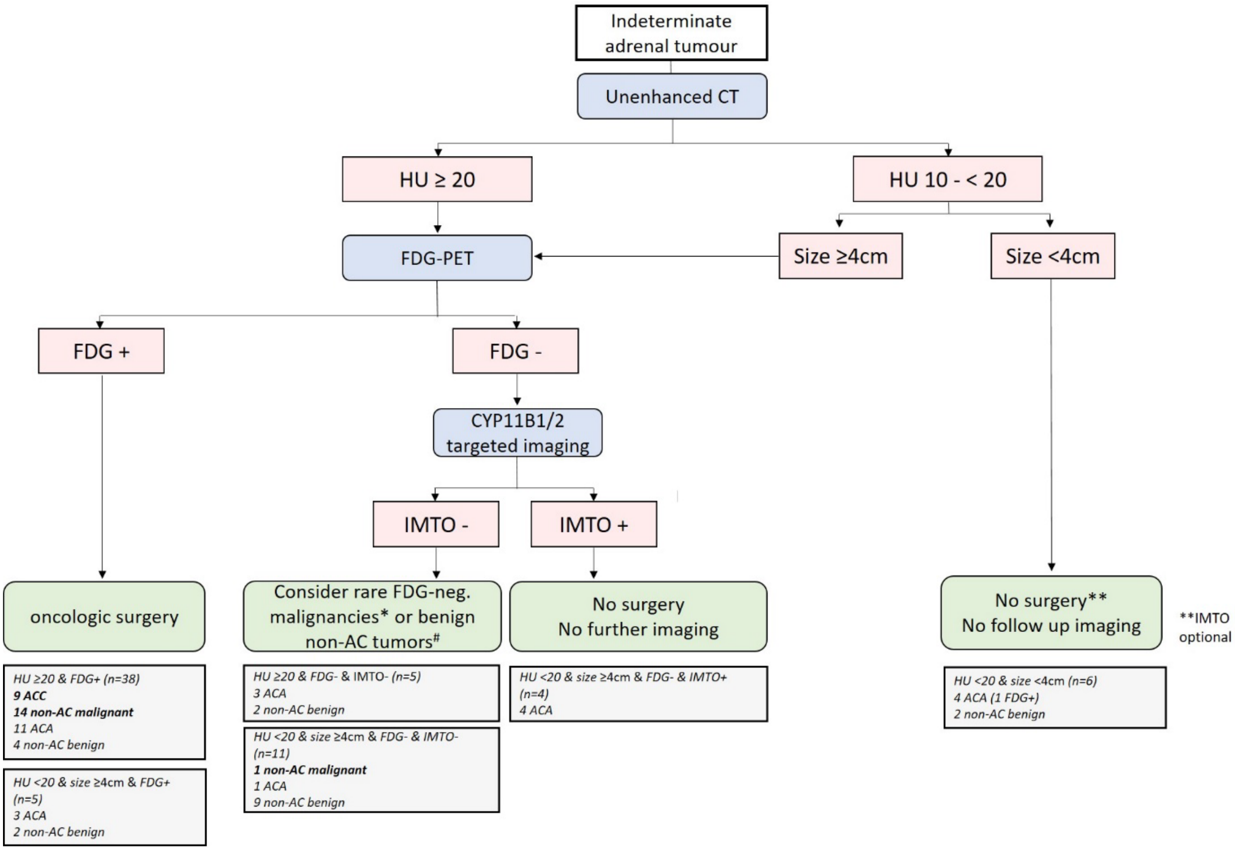

Supplementary Figure 8 Decision tree based on present study and current recommendations.

In the FAMIAN study, 63 tumours had HUs ≥20 (9 ACC, 15 non-AC malignant, 23 ACA and 16 non-AC benign) and 14 tumours had Hus <20 (7 ACA, 7 non-AC benign). The FAMIAN study found all lesions with <20 HU to be benign, supporting the use of <20 HU as a safe cut-off, despite current European guidelines. However, other studies have reported individual malignancies in the 10–20 HU range. While lesion size played a subordinate role in our analysis, we adopted the guideline-recommended cut-off of 4 cm. Imaging findings must always be evaluated in the overall clinical context, particularly in young patients where the risk of malignancy is higher.
For HU <20, additional CYP11B1-targeting imaging is optional to further clarify the diagnosis of ACA but no FDG-PET is regarded necessary.
For HU ≥20 and FDG-PET negative lesions our analysis identified a broad spectrum of mostly non-ACA benign diagnoses (# two bronchogenic cysts, 1 pheochromocytoma, 1 SFT tumour, 1 haemangioma, 1 ganglioneuroma, 1 lymphangioma, 1 haematoma and one malignant gastrointestinal stroma tumour) but also one ACA/myelolipoma mixed tumour and one low grade malignant gastrointestinal stroma tumour were found.
*FDG-PET-negative malignancies have also been observed elsewhere, particularly metastases of renal cell carcinoma and hepatocellular carcinoma. CYP11B1 imaging may be of value in these tumours with HUs ≥20 but are negative in FDG-PET. Surgical removal may be considered in the tumours with HUs ≥20 being both FDG and IMTO negative.
For FDG positive lesions ≥20 HU or large lesions we would recommend surgical removal without additional IMTO imaging.

Supplementary Table 3 Results of quantitative analysis of functional imaging with FDG or IMTO

Functional imaging findings

	**All benign (n=53)**	**ACA (n=30)**	**Non-AC benign (n=23)**	**All malignant (n=24)**	**ACC (n=9)**	**Non-AC malignant (n=15)**
**FDG-PET**						
SUV*max*	3·7 [2·6; 6·6]	4·5 [3·3; 8·5]	3·1 [1·2; 5·5]	13·0 [9·3; 29·9]	11·5 [7·9; 58·2]	13·6 [9·3; 28·2]
SUV*peak*	2·8 [1·9; 5·0]	3·4 [2·6; 6·0]	2·1 [1·0; 3·6]	10·2 [7·0; 20·9]	9·4 [6·4; 45·0]	10·3 [6·9; 20·3]
TBR_{max}/$liver_{max}$	1.0 [0·6; 1·6]	1·2 [0·8; 1·7]	0·9 [0·3; 1·1]	3·9 [2·2; 8·6]	4·0 [1·9; 16·2]	3·9 [2·3; 7·0]
TBR_{max}/$liver_{mean}$	1·5 [1·1; 2·6]	1·9 [1·4; 2·9]	1·3 [0·4; 1·8]	5·9 [3·3; 15·6]	6·2 [2·6; 27·0]	5·7 [3·5; 11·8]
**IMTO-SPECT** $_2$	**All benign (n=38)**	**ACA (n=21)**	**Non-AC benign (n=17)**	**All malignant (n=20)**	**ACC (n=7)**	**Non-AC malignant (n=13)**
SUV*max*	4·7 [1·3; 11·8]	11·3 [7·6; 24·4]	1·3 [0·9; 2·7]	1·7 [1·2; 3·0]	3·1 [1·5; 18·5]	1·7 [1·0; 2·0]
SUV*peak*	4·3 [1·4; 11·1]	10·4 [6·7; 22·3]	1·3 [1·0; 2·5]	1·7 [1·3; 2·7]	3·0 [1·5; 16·8]	1·6 [1·0; 2·0]
TBR_{max}/$liver_{max}$	5.3 [1·4; 11·1]	10.6 [6·1; 10·1]	1.0 [0·7; 2·7]	1.2 [0·9; 1·9]	1·7 [0·9; 10·9]	1.2 [0·8; 1·3]
TBR_{max}/$liver_{mean}$	6.3 [1·7; 14·3]	12.4 [7·4; 24·6]	1.2 [0·8; 3·3]	1.5 [1·1; 2·5]	2.6 [1·1; 13·2]	1.5 [1·1; 1·9]

The most commonly used quantitative measures SUVmax, SUVpeak and tumour to liver ratios are shown. Data provided as median [IQR].

TBR_{max}/$liver_{max}$ = tumour to background ratio of adrenal tumour SUVmax and liver SUVmax;

TBR_{max}/$liver_{mean}$= tumour to background ratio of adrenal tumour SUVmax and liver SUVmean

Supplementary Table 4 Characteristics of FDG-positive versus FDG-negative ACA regarding demographic data, imaging findings and histopathology findings

	FDG- (n=16)	FDG+ (n=14)
Age (years)	58·2±10·2<br>55·5 [51·5 -65·0]	59·6±11·3<br>60 0[53·8-67·5]
Sex	female 8 (50%), male 8 (50%)	female 9 (64%), male 5 (36%)
Size (cm)	4·6±1·4<br>4·4 [3·4-5·4]	4·7±1·8<br>4·3 [3·7-5·2]
Ki67 index	1·85±1·2<br>1·0 [1·0-2·5]	1·79±0·8<br>2·0 [1·0-2·25]
Weiss score	0·3±0·6<br>0·0 [0·0-0·75]	1·2±1·0<br>1·0 [1·0-2·0]
Unenhanced HU	25·1±8·2<br>24·0 [19·3-30·6]	26·6±7·3<br>28·3 [22·3-32·3]
FDG PET SUVmax	3·3±1·0<br>3·5 [2·6-4·2]	15·3±14·8<br>8·6 [5·5-28·7]
Heterogeneous in CT	6 (37·5%)	8 (57·1%)
ESE-ENSAT guideline category based on conventional imaging (categories 1-4)	Cat 2 3 (19%)<br>Cat 3 6 (38%)<br>Cat 4 7 (44%)	Cat 2 0<br>Cat 3 5 (35·7%)<br>Cat 4 9 (64·3%)

Data provided as mean±SD or n (%) or median [IQR]

ESE-ENSAT practice guideline categories for adrenal tumour morphology are defined as follows: 1) Any size, homogeneous and HU ≤10, 2) Homogenous and HU 11-20 and tumor < 4cm; 3) Homogenous HU 11-20 and tumor ≥ 4cm or Homogenous HU > 20 and tumor <4cm or Heterogeneous tumors < 4cm; 4) Homogenous HU > 20 or heterogeneous and Tumor ≥ 4cm (Fassnacht et al. Eur J Endocrinol 2023)
