# Supplementary material for: Combined [18F]Fluorodeoxyglucose PET and [123I]Iodometomidate-SPECT for diagnostic evaluation of indeterminate adrenal neoplasias—the cross-sectional diagnostic test accuracy study FAMIAN
Source: eBioMedicine. 2025 May 20;116:105735. doi: 10.1016/j.ebiom.2025.105735 (PMC12148602; doi:10.1016/j.ebiom.2025.105735)
Supplement: Transfer authorisation [file mmc2.pdf]

THE LANCET

Authorisation to transfer author statement and ICMJE forms

Please insert the relevant text under the subheadings below. A completed form must be signed by the corresponding author. Please note that we will accept hand-signed and electronic (typewritten signatures). Please complete the form, scan and email to the handling Editor.

Article originally submitted to: The Lancet Diabetes & Endocrinology

Article now submitted to: eBioMedicine

Manuscript number at new journal: EBIOM-D-24-05133 - FAMIAN

Manuscript title: Combined [18F]Fluorodeoxyglucose PET and [123I]Iodometomidate-SPECT for diagnostic evaluation of indeterminate adrenal neoplasias – the cross-sectional diagnostic test accuracy study FAMIAN

Corresponding author: Stefanie Hahner

Article type: original article

I Stefanie Hahner, the corresponding author of this manuscript confirm that all authors listed are aware that the manuscript is now being considered at eBioMedicine and have agreed to transfer the original author signature and ICMJE forms from The Lancet journal to which it was previously submitted. I (on behalf of all authors) agree that these signatures and statements are still accurate and applicable.
