# Supplementary material for: Combined [18F]Fluorodeoxyglucose PET and [123I]Iodometomidate-SPECT for diagnostic evaluation of indeterminate adrenal neoplasias—the cross-sectional diagnostic test accuracy study FAMIAN
Source: eBioMedicine. 2025 May 20;116:105735. doi: 10.1016/j.ebiom.2025.105735 (PMC12148602; doi:10.1016/j.ebiom.2025.105735)
Supplement: FAM1.7_final_clean [file mmc3.pdf]

Sponsor Protocol: FAMIAN-1.7

EudraCT: 2012-003604-13

Combined 18F-Fluorodeoxyglucose (FDG) Positron Emission Tomography (PET) and Metomidate Imaging for Adrenal Neoplasia (FAMIAN-Study) - a Diagnostic Study

Abbreviated Title of Protocol: FAMIAN

Version 1.7– Date: 26 MAY 2020

**COORDINATING INVESTIGATOR**	**Prof. Dr. med. S. Hahner**<br>University Hospital Würzburg<br>Department of Medicine I, Endocrinology and Diabetology,<br>Oberdürrbacher Str. 6, 97080 Würzburg<br>Germany<br>e-mail: hahner_s@ukw.de

**SPONSOR**	**University Hospital Würzburg**<br>**Josef-Schneider-Str. 2**<br>**97080 Würzburg**	Date and Signature

TABLE OF CONTENTS

I. STUDY TEAM **6**

II. STUDY SYNOPSIS **10**

III. ABBREVIATIONS **13**

1 INTRODUCTION AND RATIONALE OF THE STUDY **15**

1.1 BACKGROUND: DIAGNOSTIC PROBLEMS IN ADRENAL NEOPLASIA 15

1.2 THE NEED FOR A TRIAL 17

1.3 RISK-BENEFIT CONSIDERATIONS 17

1.3.1 Risk and safety aspects of FDG-PET and [^{123}I]Iodometomidate imaging *17*

1.3.2 Benefits *18*

2 STUDY OBJECTIVES **18**

2.1 PRIMARY OBJECTIVE 18

2.2 SECONDARY OBJECTIVES 18

2.3 EXPLORATORY OBJECTIVES 19

3 METHODOLOGY **19**

3.1 STUDY DESIGN RATIONALE 19

3.2 STUDY PLAN 19

3.3 PREMATURE TERMINATION 20

3.3.1 Premature closure of a trial site *20*

3.3.2 Premature termination of the trial for individual patients *20*

3.3.3 Premature termination of the trial *21*

4 SELECTION OF PATIENTS **21**

4.1 INCLUSION CRITERIA 21

4.2 EXCLUSION CRITERIA 22

5 INVESTIGATIONAL PRODUCT **23**

5.1 [^{123}I]IODOMETOMIDATE 23

5.2 RADIOLABELLING OF THE TRACER AND BIODISTRIBUTION 24

5.3 FDG 24

6 DIAGNOSTIC STUDIES **26**

6.1. FDG-PET, FDG-PET/CT 26

6.2 [^{123}I]IODOMETOMIDATE-SPECT 26

6.3 CENTRALIZED IMAGING REVIEW 27

7 TRIAL PROCEDURE 28
7.1 SCREENING 28
7.1.1 Informed Consent 28
7.1.2 Enrolment and screening visit 28
7.2 DIAGNOSTIC PHASE 30
7.3 FOLLOW-UP PHASE 31
7.4 STUDY DURATION 31
7.5 SUBJECT DISCONTINUATION 32
7.6 TABLE OF EVENTS OR VISIT SCHEDULE AND ASSESSMENTS 32
7.6.1 Screening failure 32
7.6.2 Safety Assessements 34
7.7 VIOLATION OF ELIGIBILITY CRITERIA 34
8 BIOMETRICAL ASPECTS 35
8.1 OUTCOME VARIABLES AND MEASURES 35
8.1.1 Primary variables and measures 35
8.1.2 Secondary variables and measures 35
8.1.3 Exploratory endpoints 36
8.2 STATISTICAL HYPOTHESIS AND CONFIRMATORY STATISTICAL ANALYSIS 36
8.3 PROPOSED SAMPLE SIZE / POWER CALCULATIONS 37
8.4 COST EFFECTIVENESS ANALYSIS / STEROIDOBOLOMICS 37
8.5 BACKGROUND AND DEMOGRAPHIC CHARACTERISTICS 38
8.6 SAFETY ANALYSIS 38
9 ADVERSE EVENTS AND SERIOUS ADVERSE EVENTS 39
9.1 DEFINITION 39
9.1.1 Adverse Event (AE) 39
9.1.2 Serious Adverse Event (SAE) 39
9.2 EXPOSURE TO IMAGING DURING PREGNANCY/LACTATION 40
9.3 RECORDING OF ADVERSE EVENTS 41
9.3.1 When to collect AEs 41
9.3.2 Adverse Event (AE) and Serious Adverse Event (SAE) Documentation and Reporting 41
9.3.3 Severity assessment 42
9.3.4 Causality assessment 43
9.3.5 Duration 43
9.3.6 Action Taken 43
9.3.7 Outcome 43

9.4 EXPEDITED REPORTING OF ADVERSE EVENTS 44
9.5 RESPONSIBILITIES OF THE COORDINATING INVESTIGATOR 45
9.5.1 Definition of SUSARs 45
9.5.2 Documentation and Reporting 45
9.5.3 Annual safety report 45
10 END OF STUDY 46
11 COMMITTEES AND PANEL REVIEW 46
11.1 STEERING COMMITTEE 46
11.2 INDEPENDENT DATA MONITORING COMMITTEE 46
12 ETHICAL AND REGULATORY ASPECTS 47
12.1 SPONSOR OBLIGATIONS 48
12.2 INFORMATION AND CONSENT OF PARTICIPANTS 48
12.3 INVESTIGATOR RESPONSIBILITIES 49
13 DATA COLLECTION 49
14 QUALITY ASSURANCE - MONITORING 49
15 DATA OWNERSHIP / PUBLICATION POLICY 50
16 REFERENCES 51
17 APPENDIX 53
17.1 GRADE ECOG (EASTERN COOPERATIVE ONCOLOGY GROUP) 53
17.2 STANDARD OPERATING PROCEDURES FOR COLLECTION AND ANALYSIS OF PATHOLOGY DATA 54
17.3 STANDARD OPERATING PROCEDURES FOR [123I]-IODOMETOMIDATE IMAGING AND FDG-PET/CT WITHIN THE FAMIAN TRIAL 55
17.3.1 [^{123}I]Iodometomidate scintigraphy (planar imaging and SPECT/CT) 55
17.3.2 18F-FDG-PET/CT 58
17.4 STANDARD OPERATING PROCEDURES FOR COLLECTION, STORAGE, AND SHIPPING OF BIOLOGICAL SPECIMENS 62
17.5 NATIONAL CANCER INSTITUTE - COMMON TERMINOLOGY CRITERIA FOR ADVERSE EVENTS 64
17.6 SAE (SERIOUS ADVERSE EVENT) FORM 65
17.7 SIGNATURE PAGE 67

I. STUDY TEAM

**Sponsor**<br>*According to German law*	**Universitätsklinikum Würzburg**	Josef-Schneider-Str. 2<br>97080 Würzburg, Germany<br>Holder of the legal duties and tasks of the sponsor (as a professional task): Prof. Dr. S. Hahner
**Coordinating Investigator/**<br>***Leiterin der Klinischen Prüfung***	**Stefanie Hahner**	Universitätsklinikum Würzburg<br>Medizinische Klinik und Poliklinik I<br>Oberdürrbacher Str. 6<br>97080 Würzburg, Germany<br>Phone: +49 931 201-39020 and -39200<br>Fax +49 931 201-639200<br>E-mail: hahner_s@ukw.de
**Biometry**	**Hans-Helge Müller**	Philipps-Universität Marburg<br>Institut für Biometrie und Epidemiologie<br>Bunsenstr. 3<br>35032 Marburg, Germany<br>Phone: +49 6421 28-66209<br>Fax: +49 6421 28-68921<br>E-mail: hans-helge.mueller@uni-marburg.de
**Safety Office**		Universitätsklinikum Würzburg<br>Medizinische Klinik und Poliklinik I/Endokrinologie<br>Oberdürrbacher Str. 6<br>97080 Würzburg, Germany<br>Phone: +49 931 201-39716/-39020/-39200<br>Fax +49 931 201-6039716<br>E-Mail: famian@ukw.de

**Data Monitoring and Safety Board (DMSB)**	**Ulrich Mansmann**	IBE - Institut für Medizinische Informations-verarbeitung, Biometrie und Epidemiologie<br>Ludwig-Maximilians-Universität München<br>Marchioninistr. 15<br>81377 München, Germany<br>Phone: +49 89 7095-4490<br>Fax: +49 89 7095-7491<br>E-mail: mansmann@ibe.med.uni-muenchen.de
	**Markus Schwaiger**	Nuklearmedizinische Klinik und Poliklinik<br>Technische Universität München<br>Klinikum rechts der Isar<br>Ismaninger Straße 22<br>81675 München, Germany<br>Phone: +49 89 4140-2970<br>E-mail: markus.schwaiger@tum.de
	**Bernhard Böhm**	LKC Medicine<br>NTU and Imperial College London<br>11 Mandalay Road<br>Singapore 308232<br>and<br>Tan Tock Seng Hospital<br>11 Jalan Tan Tock Seng<br>Singapore 308433<br>Phone: +65 65923637<br>E-mail 1: Bernhard.boehm@ntu.edu.sg<br>E-mail 2: Bernhard.o.boehm@mailbox.org

**Tracer Supply**		Universitätsklinikum Würzburg<br>Klinik für Nuklearmedizin, Radiochemisches Labor<br>Oberdürrbacher Str. 6<br>97080 Würzburg, Germany<br>Phone: +49 931 201-35310
		MAP Medical Technologies Oy<br>Elementtitie 27<br>41160 Tikkakoski, Finnland<br>Phone: +358 14 334-5211
**Steering Committee**	**Felix Beuschlein**	UniversitätsSpital Zürich<br>Klinik für Endokrinologie, Diabetologie und Klinische Ernährung<br>Raemistr. 100<br>CH-8091 Zürich<br>Phone +41 44 255 36 25<br>Fax +41 44 255 44 47<br>E-mail:Felix.Beuschlein@usz.ch
	**Andreas Buck**	Universitätsklinikum Würzburg<br>Klinik für Nuklearmedizin<br>Oberdürrbacher Str. 6<br>97080 Würzburg, Germany<br>Phone: +49 931 201-35000<br>Fax +49 931 201-635000<br>E-mail: buck_a@ukw.de
	**Marc Gurnell**	University of Cambridge<br>Department of Medicine<br>Addenbrooke´s Hospital<br>Hills Road<br>Cambridge CB2 0QQ, UK<br>Phone: +44 1223 586908<br>Fax: +44 1223 217080<br>E-mail: mg299@mole.bio.cam.ac.uk

**Steering Committee cont.**	**Stefanie Hahner**	Universitätsklinikum Würzburg<br>Medizinische Klinik und Poliklinik I<br>Oberdürrbacher Str. 6<br>97080 Würzburg, Germany<br>Phone: +49 931 201-39200<br>Fax +49 931 201-639200<br>E-mail: hahner_s@ukw.de
	**Massimo Mannelli**	University of Florence<br>Dept of Clinical Pathophysiology,<br>Endocrine Unit<br>Viale Pieraccini 6<br>50139 Florence, Italy<br>Phone: +39 055 4271428<br>Fax: +39 055 4221931<br>E-mail: m.mannelli@dfc.unifi.it
	**Florence Tenenbaum**	Assistance Publique Hôpitaux de Paris<br>Hôpital Cochin<br>Service de Médecine Nucléaire<br>27, Rue du Fbg St Jacques<br>75014 Paris, France<br>Phone: +33 1 58 41 21 79<br>Fax: + 33 1 58 41 21 85.<br>E-mail: florence.tenenbaum@cch.aphp.fr
	**Henri Timmers**	Radboud University Nijmegen Medical Centre<br>Departments of Endocrinology,<br>6500 HB Nijmegen, Netherlands<br>Phone: +31 24 3614599<br>Fax: +31 24 3618809<br>E-mail: h.timmers@endo.umcn.nl

II. STUDY SYNOPSIS

A) IDENTIFICATION OF CLINICAL TRIAL		
**EUDRACT NUMBER: 2012-003604-13**<br>**VERSION AND DATE: VERSION 1.7, 26 MAY 2020**		
**STUDY TITLE:**<br>**Combined 18F-Fluorodeoxyglucose (FDG) Positron Emission Tomography (PET) and Metomidate Imaging for Adrenal Neoplasia (FAMIAN-Study)**		
**ABBREVIATED TITLE: FAMIAN**		
**COORDINATING INVESTIGATOR: Stefanie Hahner**		
Total	**NUMBER OF CENTRES:**<br>**~ 15**	**NUMBER OF PATIENTS:**<br>**220**

B) SPONSOR
University Hospital Würzburg<br>Josef-Schneider-Str.2, 97080 Würzburg<br>Holder of the legal duties and tasks of the sponsor (as a professional task): **Prof. Dr. med. Stefanie Hahner**

C) GENERAL INFORMATION ON STUDY
**INDICATION:** Adrenal Neoplasia
**METHODOLOGY:** Diagnostic phase III, international, multicentre study
**PRIMARY OBJECTIVE:**<br>Evaluation of the utility of combined FDG-PET and [^{123}I]Iodometomidate imaging for non-invasive characterization of indeterminate adrenal neoplasias:<br>Demonstration of the effectiveness of a non-invasive test for benign adrenocortical adenomas (AA).
**OUTCOME MEASURES:**<br>Primary efficacy endpoint: Classification AA/non-AA and diagnostic test result AA+/AA- for specificity (rate estimation) of the diagnostic AA test and for the likelihood ratio of a positive diagnostic test (using rate estimation of the sensitivity).<br>Key secondary endpoint(s): Sensitivity of the diagnostic AA test and likelihood ratio of a negative diagnostic AA test, classification of indeterminate adrenal neoplasias for a priori rates of AA, adrenocortical cancer (ACC) and other benign

C) GENERAL INFORMATION ON STUDY cont.

as well as malignant indeterminate adrenal neoplasias. Detection rates similar to sensitivity and specificity for identifying ACC and indeterminate adrenal neoplasias other than AA and ACC using combined 18F-FDG-PET imaging and [^{123}I]Iodometomidate imaging. Predictive values will be determined and economic analysis of cost effectiveness of diagnostic evaluation versus surgery for all indeterminate lesions will be performed.

Assessment of safety: Adverse events, standard routine parameter changes from before imaging to Visit 4 will be assessed.

INCLUSION CRITERIA:

- Patients with a solid indeterminate adrenal mass scheduled for surgery or biopsy (within 3 months) and a diameter > 3 cm or an increase in tumor diameter of > 1 cm in follow-up imaging. In unenhanced computerized tomography (CT) the attenuation value of the adrenal tumor is ≥ 10 Hounsfield units (HU)
- Age ≥ 30 years
- Written informed consent
- ECOG performance status 0-2
- Effective contraception in female patients of childbearing potential
- Negative pregnancy test in women of childbearing potential
- Ability to comply with the protocol procedures

EXCLUSION CRITERIA:

- Pregnancy or breast feeding
- Patient unfit or unwilling to undergo surgery / biopsy
- Diagnosis of pheochromocytoma
- Diagnosis of primary hyperaldosteronism
- Diagnosis of adrenal Cushing's syndrome

D) DESCRIPTION OF STUDY TREATMENTS

Patients with a clinically indeterminate adrenal mass will be investigated by 18F-FDG-PET and [^{123}I]Iodometomidate SPECT prior to surgery. For Non-German sites only the use of [^{11}C]Metomidate PET instead of [^{123}I]Iodometomidate SPECT will be permitted.

TREATMENT DURATION: Single combined diagnostic evaluation

E) SAMPLE SIZE DETERMINATION

Sample size calculation is based on the two primary hierarchically structured two-sided level 5% tests. For the first test, assuming an a priori probability of 60% for AA and a specificity (P(AA- | non-AA)) of 98% to be detected with a power of 80%, approximately 195 assessed patients are necessary. For the exact test, at least 70 patients with a confirmed non AA neoplasia are necessary. For the second test, assuming an a priori probability of 60% for AA and a specificity of 98% together with a sensitivity (P(AA+|AA)) of 92% to be detected with a power of 80%, approximately 187 assessed patients are necessary. Thus 195 patients to be assessed are necessary. Adjusting for incomplete assessments in up to 10% of patients and a recruitment rate of 40% within screened patients, 220 patients have to be recruited and 550 to be screened. For the third test, assuming an a priori probability of 10% for ACC (~20 ACC patients), the power to detect P(AA-|ACC)=98% is ~70%. For the fourth test, assuming a priori probabilities of 60% for AA and 10% for ACC, the power to detect P(AA-|ACC)=98% together with P(AA+|non-ACC)=61.5% is almost 80%. The assumptions on a priori probabilities and detectable probabilities to be powered were set up in consensus at the ENSAT Cancer Imaging Study Group meeting at the 1st European Conference on Adrenal Imaging 2011.

F) DURATION OF STUDY

INCLUSION PERIOD:

First patient in to last patient out: July 2015 to January 2023

Duration of the entire trial (months): 90 months

TREATMENT PERIOD: single diagnostic evaluation

FOLLOW-UP PERIOD: 2 - 4 weeks

OVERALL DURATION OF STUDY: 7,5 years

III. ABBREVIATIONS

AA	Adrenocortical adenoma
ACC	Adrenocortical carcinoma
AE	Adverse event
ARR	Aldosterone-to-Renin-Ratio
ARQ	Aldosterone-to-Renin-Quotient
ASR	Annual safety report
BfArM	Bundesinstitut für Arzneimittel und Medizinprodukte
CE	Contrast enhanced
CgA	Chromogranin A
CRA	Clinical Research Associate
CT	Computerized tomography
CYP11B	Cytochrome P450 B11
DRC	Direct Renin Concentration
EudraCT	European database of clinical trials
FDG	Fluorodeoxyglucose
GCMS	Gas Chromatograph Mass Spectrometer
HPLC	High pressure liquid chromatography
HU	Hounsfield unit(s)
IB	Investigator brochure
IMP	Investigational medicinal product
IMTO	Iodometomidate
ISF	Investigator site file
MRI	Magnetic resonance imaging
MedDRA	Medical Dictionary for Regulatory Activities
PET	Positron emission tomography
PAC	Plasma Aldosterone Concentration
PI	Principal investigator

PRA	Plasma Renin Activity
PRC	Plasma Renin Concentration
SADR	Serious adverse drug reaction
SAE	Serious adverse event
SPECT	Single photon emission computed tomography
SUSAR	Suspected unexpected serious adverse reaction
TMF	Trial master file

1 INTRODUCTION AND RATIONALE OF THE STUDY

The prevalence of incidentally discovered adrenal masses on CT examinations has been reported to be 0.35–5.0% (1). Biochemical testing is necessary to distinguish between functional and non-functional adrenal masses. In hormonally inactive tumors it is a major diagnostic challenge to differentiate between benign and malignant lesions, as the perceived risk of malignancy defines the necessity of surgical intervention. While CT and MRI imaging protocols often allow ruling out malignancy due to a high fat content of the adrenal lesion, a significant subgroup of patients remains with indeterminate tumors after standard imaging. FDG-PET holds significant potential to separate benign from malignant lesions (2). However, its specificity concerning the origin of the adrenal mass (e.g. metastases or adrenocortical carcinoma) is rather limited. Fine needle biopsy is of limited help and may even be contraindicated in case of adrenocortical carcinoma. As therapeutic strategies differ between the various entities (e.g. between metastatic disease and primary adrenocortical cancer) non-invasive characterization of adrenal neoplasia prior to surgery would be of great value. Iodometomidate binds with high specificity and affinity to CYP11B enzymes of the adrenal cortex. As these enzymes are exclusively expressed in adrenocortical cells, uptake of labelled metomidate tracers has been shown to be highly specific for adrenocortical neoplasia (3-7). We, therefore, hypothesize that combined use of 18F-FDG-PET and [^{123}I]Iodometomidate imaging will allow to non-invasively characterize adrenal neoplasia as adrenocortical adenoma and will significantly affect therapeutic strategies, as hormonally inactive adenomas do not require surgical removal.

1.1 Background: Diagnostic problems in adrenal neoplasia

The management of adrenal tumors is a public health challenge of growing importance, as these tumors are detected with increasing frequency in a population that has widespread access to advanced imaging technology. The key question is, whether or not such a tumor needs to be removed by surgery. Basically this question can be divided into two separate questions:

- Is the adrenal lesion hormonally active?
- Is the tumor benign or malignant?

In non-oncological patients, adrenocortical carcinoma (ACC) is the most frequently observed adrenal malignancy with a prevalence of 4-6%. Currently, tumor size and a low lipid content of an adrenal mass and the rapidity of the wash-out of contrast medium are probably the best criteria for diagnosing ACC. Benign adrenocortical adenomas usually have a high content of lipids, resulting in a low attenuation coefficient on unenhanced CT (< 10 Hounsfield units (HU)). Furthermore, on delayed contrast-enhanced CT adenomas typically exhibit rapid wash-out of contrast medium, whereas non-adenomatous tumors display delayed wash-out of contrast material (8). However, despite these highly useful criteria, a significant number of adrenal incidentalomas (around 30% of tumors > 3 cm) are not reliably characterized

by these standard imaging procedures and for these tumors a diameter of 4 cm is considered an indication for surgery, although based on histopathology the majority of these cases are benign adenomas. Of note, fine-needle biopsy is not indicated in adrenal incidentalomas, as it is often non-informative and potentially hazardous. Furthermore, several tumors, including carcinomas of the lung, breast, kidney and melanoma frequently metastasize into the adrenal glands and adrenal metastasis may be the first indication of a yet unknown malignancy. Thus, improved tools for characterizing adrenal masses prior to surgery are of great clinical interest. In adrenal tumors, 18F-FDG-PET was first evaluated in patients with known extra adrenal malignancies. Qualitative visual evaluation was considered as equally accurate as quantitative evaluation using standardized uptake values (SUV) for identification of adrenal metastases with a specificity of 100% but only a sensitivity of 59.5%. However, *Schulkin et al.* demonstrated that also pheochromocytomas frequently show an increased FDG uptake on PET (9). Furthermore, malignant adrenal lesions that may produce false negative PET results have also been reported in patients with adrenal metastases, e.g. secondary to pulmonary carcinoid, renal and alveolar lung carcinoma. In a series of 105 patients with incidental adrenal masses, adrenocortical carcinomas demonstrated a high FDG uptake, as did the majority of pheochromocytomas (10). Furthermore, adrenal metastases also demonstrated increased FDG activity (n = 9). These findings indicate that FDG-PET has good accuracy to assess the malignant potential of an adrenal lesion without providing robust information on the origin of the adrenal mass.

More recently, metomidate derivatives which bind with high specificity and avidity to CYP11B enzymes of adrenocortical cells have been developed as tracers for adrenal imaging. These tracers have the advantage of a highly specific binding to molecules that are uniquely expressed in adrenocortical cells. The following tracers were introduced into clinical practice:

- $[^{11}C]$Metomidate (MTO) for PET imaging,
- $[^{18}F]$Fluoro-etomidate (FETO) and
- $[^{123}I]$Iodometomidate.

For example, *Juhlin et al.* found high uptake of MTO in all tumors originating from the adrenal cortex, whereas all other processes where negative (11). Similarly, *Minn et al.* found the highest uptake in ACC followed by active adenomas and non-secretory adenomas and very low uptake in non-cortical tumors (12). In a retrospective evaluation of 212 MTO-PET examinations in 173 patients *Hennings et al.* reported that non-adrenocortical lesions were negative in MTO-PET (13). *Zettinig et al.* investigated 16 patients with 18F-FDG-PET and $[^{11}C]$Metomidate and demonstrated that metomidate could distinguish adrenocortical from non-adrenocortical tissue, whereas 18F-FDG PET differentiated malignant from benign disease (14). Similar results have been obtained with $[^{123}I]$Iodometomidate with the clear advantage that this SPECT tracer does not require the local availability of a cyclotron, facilitating its widespread use and also demonstrating high specificity (7). Taken together, these studies indicate that this new imaging modality might confer significant specificity to the characterization of adrenal neoplasia. However, previous series using metomidate imaging included a wide variety of tumors, frequently independent of the indication for surgery. Thus, histopathological confirmation was sometimes lacking and often the

differential diagnosis was already solved prior to these imaging studies precluding a clear analysis of the clinical utility of this new technology. In fact, the most relevant patient group consists of patients with a larger adrenal incidentaloma (> 3 cm) without significant hormonal activity and uncertain malignant potential based on standard imaging (CT/MRI).

We hypothesize that such adrenal lesions have a very high probability of being a benign adrenocortical adenoma not requiring surgery, if they show low uptake values in FDG-PET and high uptake in metomidate imaging. In contrast, tumors with significant metomidate uptake and high FDG uptake represent with high probability adrenocortical carcinomas requiring surgical removal by an expert surgeon.

1.2 The need for a trial

No previous study has systematically assessed the utility of the combination of FDG-PET imaging and metomidate imaging in patients with indeterminate adrenal neoplasia. However, such an evaluation is of great clinical value, as evidence for a benign adrenocortical lesion would obviate the need for adrenal surgery, whereas evidence for the presence of an adrenocortical carcinoma would require a demanding surgical approach by a highly experienced adrenal surgeon. In contrast, in patients with high FDG-PET uptake and no uptake in metomidate imaging a high likelihood for metastatic disease or a non-adrenocortical primary malignancy could probably be concluded. Thus, the systematic and prospective evaluation of combined FDG-PET and [^{123}I]Iodometomidate imaging in a patient group in which the decision for surgery remains problematic is of great diagnostic interest.

1.3 Risk-benefit considerations

1.3.1 Risk and safety aspects of FDG-PET and [^{123}I]Iodometomidate imaging

FDG-PET and CT are established imaging modalities that have already been extensively validated in routine clinical practice. Integrated PET/CT combines PET and CT and allows morphological and functional imaging to be carried out in a single imaging procedure allowing accurate lesion localisation and characterisation. The safety aspects of FDG-PET are related exclusively to radiation exposure. The radiation dose delivered to the patient by i.v.-injection of 350 MBq FDG is approximately 6.7 mSv. The radiation exposure related to a CT scan performed as part of a PET/CT examination depends on the use of a low-dose CT protocol or a diagnostic CT including i.v. contrast enhancement. Accordingly, a range from 1-20 mSv has been reported as effective dose related to CT-scanning (15). Within the FAMIAN trial, a low-dose CT will in general be sufficient. Hence, contrast-enhanced (CE) CT will serve as mainstay diagnostic test. If a recent CE-CT - performed up to 4 weeks prior to inclusion - is available, low-dose CT can be performed instead. However, the imaging protocol performed in individual patients will strongly

depend on the clinical situation and may differ significantly among patients (e.g., with a history of malignancy).

Similarly, for [^{123}I]Iodometomidate, the key safety issue is the radiation exposure. Administration of [^{123}I]Iodometomidate was associated with a self-limiting flush in a small number of patients, an adverse event likely related to the solvent containing Tween 80. After omitting Tween 80, no further adverse events related to the administration of [^{123}I]Iodometomidate were noted in a previous trial (16). During long-term follow-up (> 2 years) no side effects attributable to [^{123}I]Iodometomidate imaging were observed (4). The mean effective radiation dose per administered activity was calculated to be 0.015 mSv/MBq (range: 0.011 – 0.020 mSv/MBq) corresponding to 2.7 mSv for a typical investigation with 185 MBq [^{123}I]IMTO.

Long-term risks from radiation exposure are age-dependent and highest in children. To minimize long-term radiation effects, only patients aged 30 years or older are included.

1.3.2 Benefits

No immediate general benefits for the participating patients are anticipated in the FAMIAN trial, as surgical removal of the adrenal lesion is intended in all patients. However, high standardized uptake values (SUV) in FDG-PET suggest a malignant lesion and the combination of positive FDG-PET and [^{123}I]Iodometomidate uptake by the adrenal mass indicates a high probability of an adrenocortical carcinoma requiring a specific surgical approach by a particularly experienced surgeon. Furthermore, in FDG-PET, and also in [^{123}I]Iodometomidate imaging, additional foci of uptake may point to the presence of further lesions which may alter the surgical strategy or may even change the entire treatment concept. Thus, individual patients may benefit from the information gained using the two additional imaging procedures.

2 STUDY OBJECTIVES

2.1 Primary objective

Evaluation of the utility of combined FDG-PET and [^{123}I]Iodometomidate imaging for non-invasive characterization of indeterminate adrenal neoplasias: Demonstration of the effectiveness of a non-invasive test to diagnose benign adrenocortical adenomas (AA).

2.2 Secondary objectives

Evaluation of the utility of combined FDG-PET and [^{123}I]Iodometomidate imaging for diagnosis of ACC versus non-ACC, evaluation of FDG-PET as a predictor of malignancy in adrenal neoplasia. In addition, a cost effectiveness analysis will be performed comparing imaging costs with potentially avoidable costs for surgery.

2.3 Exploratory objectives

Analysis of the prevalence of the respective diagnoses (Adrenal adenoma, ACC, metastases and others) in this patient cohort, analysis of urinary steroid secretion by GC-MS as a non-invasive tool for the diagnosis of ACC.

3 METHODOLOGY

3.1 Study Design Rationale

This is a diagnostic study using combined FDG-PET and [^{123}I]Iodometomidate imaging in indeterminate adrenal neoplasia prior to surgery.

All tumors will be characterized as either positive or negative concerning FDG-/Iodometomidate uptake leading to 4 different categories. The diagnostic measures of primary interest are sensitivity and specificity of the combined use of FDG-PET and Iodometomidate imaging using histopathology of the surgically removed tumor as gold standard for the differential diagnosis. The primary clinical outcome is the correct non-invasive diagnosis (using the combined AA test) of histologically verified adrenocortical adenoma and verified neoplasia different from adrenocortical adenoma (for the estimation of the sensitivity and the specificity of the combined AA test). However, all removed neoplasias will be classified according to histopathological criteria. Key secondary outcome measures are a priori probabilities of AA, ACC and other benign as well as malignant adrenal incidentalomas and positive and negative predictive values. In addition, a cost effectiveness analysis will be performed comparing imaging costs with potentially avoidable costs for surgery. The histopathological diagnosis will be performed by a reference pathologist based on established criteria.

3.2 Study Plan

The FAMIAN trial is a phase III diagnostic trial, using an investigational tracer, [^{123}I]Iodometomidate, for imaging of adrenocortical CYP11B enzymes together with FDG-PET in indeterminate adrenal neoplasia. It is hypothesized that tumors positive for [^{123}I]Iodometomidate and negative for FDG-PET are benign adenomas not requiring surgery based on histopathological diagnosis. The study flow chart is shown in Fig. 1.

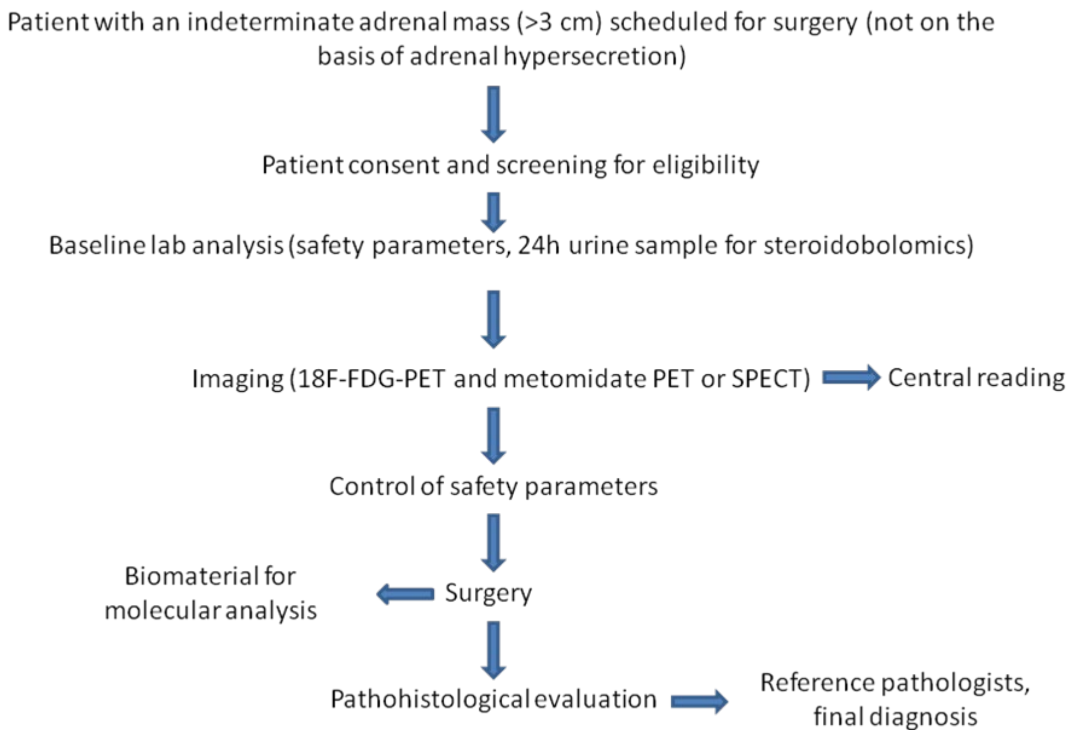

Fig.1 Study Flow Chart

3.3 Premature termination

3.3.1 Premature closure of a trial site

Premature closure of a trial site is to be considered if:

- Conduct of the study is not in compliance with the trial protocol
- Quality of data delivered by the study site is not sufficient to warrant trial performance according to the German AMG and international GCP regulations.

The premature closure of a study site will be decided by the principal and coordinating investigator after consultation with the responsible biometrician and the Steering Committee. Individual investigators and / or whole trial sites deciding not to participate in the trial any longer have to inform the coordinating investigator immediately in writing.

3.3.2 Premature termination of the trial for individual patients

The study might be terminated early in individual patients for the following reasons:

- Pregnancy
- The patient’s withdrawal of consent
- Safety concerns or suspected incompliance with imaging procedures

3.3.3 Premature termination of the trial

In case of the following situations, a premature termination of the trial has to be considered:

- Serious adverse reactions to [^{123}I]Iodometomidate / unexpected not justifiable toxicity
- Substantial changes in risk-benefit considerations
- New insights from other trials
- Insufficient recruitment rate of all study sites (see Stopping Rules)
- Unsustainable trial organization

The Data Monitoring and Safety Board (DMSB) will – based on interim reports - monitor the study conduct and all safety aspects of the trial and will give recommendations to the Steering Committee whether to stop the trial or to change the trial protocol. Decisions regarding premature termination of the trial will jointly be taken by the Steering Committee and the Data Monitoring and Safety Committee. According to the German drug law, the trial may be also suspended or prematurely terminated by decision of the competent federal authority (Bundesinstitut für Arzneimittel und Medizinprodukte – BfArM).

4 SELECTION OF PATIENTS

4.1 Inclusion criteria

1. Patients with a solid indeterminate adrenal mass
   - scheduled for surgery or biopsy (within 3 months) **AND**
   - a diameter > 3 cm or an increase in tumor diameter of > 1 cm in follow-up imaging *(Note: Imaging data should be no older than 3 months. However, if all criteria have been fulfilled in preceding imaging – i.e. solid tumor > 3 cm, HU > 10 – patient can be included and the necessary up-to-date imaging can be acquired in combination with the FDG-PET after study inclusion.)* **AND**
   - an attenuation value of the adrenal tumor ≥ 10 Hounsfield units (HU) in unenhanced computerized tomography (CT)
2. Age ≥ 30 years
3. Written informed consent
4. ECOG performance status 0-2
5. Effective contraception in female patients of childbearing potential (defined as all women physiologically capable of becoming pregnant) for minimum of 30 days after IMTO or the next menstrual bleeding – whichever comes first
6. Negative pregnancy test in women of childbearing potential (defined as all women physiologically capable of becoming pregnant)

7. Ability to comply with the protocol procedures

4.2 Exclusion criteria

1. Pregnancy or breast feeding
2. Patient unfit or unwilling to undergo surgery / biopsy
3. Diagnosis of pheochromocytoma (diagnosis of pheochromocytoma is based on the clinical decision of an experienced endocrinologist taking into account the results of measurement of plasma metanephrines and normetanephrines OR urine metanephrines and normetanephrines in combination with further clinical information)
4. Diagnosis of primary hyperaldosteronism (diagnosis of hyperaldosteronism is based on the clinical decision taking into account the ratio of plasma or serum aldosterone concentration to plasma renin concentration (ARR / ARQ). In case of elevated aldosterone to renin ratio in combination with arterial hypertension, a saline infusion test OR captopril test should be performed to exclude primary aldosteronism)

   (*Note: In case of doubt or for further information please refer to: The Management of Primary Aldosteronism: Case Detection, Diagnosis, and Treatment: An Endocrine Society Clinical Practice Guideline.* (17))
5. Diagnosis of adrenal Cushing's syndrome, confirmed by

   morning serum cortisol after dexamethasone (1 mg at 23.00 h) > 5 µg/dl (140 nmol/l)

   AND Plasma ACTH < 5 ng/l

   AND either urinary free cortisol levels twice the upper limit of normal

   or bed time (23.00 h) salivary cortisol thrice the upper limit of normal

   (*Note: Since all of the 3 criteria above have to be fulfilled to exclude a patient, the patient is eligible if any single one of the tests does not meet the exclusion criteria – i.e. if ACTH > 5 ng/l, the patient is eligible regardless of the other results. However, it is strongly recommended to perform the dexamethasone suppression test as part of the standard work-up.)*

5 INVESTIGATIONAL PRODUCT

5.1 [^{123}I]Iodometomidate

[^{123}I]Iodometomidate was developed as a highly specific tracer for the characterization of adrenal masses. This tracer binds with high affinity to 11ß-hydroxylase (CYP11B1) and aldosterone synthase (CYP11B2), two enzymes exclusively expressed in adrenocortical tissue. In an initial phase I/II study, 36 patients with an adrenal lesion and 14 patients with known metastatic adrenocortical carcinoma were investigated using 185 MBq [^{123}I]Iodometomidate. Both, adrenals and adrenocortical tumor tissue, were first detected within 60 min after injection of [^{123}I]IMTO, with very good delineation of the adrenals and the tumor lesions 4-6 hours after injection. Best target to background ratios were observed at 24 hours p.i. with specific uptake exclusively in the adrenocortical tissue. Both, renal elimination and hepatobiliary excretion with transient tracer accumulation in the gallbladder and the bowels, were noted. Only temporary tracer accumulation in the early phase was observed in the brain, liver and the kidneys.

According to the applied grading system, all tumors of the 16 patients with a benign adrenocortical lesion were graded as 1 (definite adrenocortical origin), 9 tumors of 14 patients with adrenocortical carcinoma were graded as 1, one tumor as 2a (probably adrenocortical), two as 2b (indeterminate, more probably non-adrenocortical) and one tumor was graded as 3. Of the non-adrenocortical lesions 3 of 20 lesions were characterized as 1 (one pheochromocytoma and two metastases), four tumors were scored as 2 (one 2a, two 2b) whereas the remaining 13 lesions were correctly graded as 3. Qualitative analysis resulted in a sensitivity of 89% and a specificity of 85%. Remarkable is the perfect sensitivity (100%) in the detection of adrenocortical adenomas. The uptake of [^{123}I]Iodometomidate in adrenocortical tissue was considerably high and specific, enabling successful radiotherapy of selected patients with advanced adrenocortical carcinoma using a related compound which has been radiolabeled with the ß-emitter 131I ([^{131}I]IMTO) (4).

Observed adverse events were either unrelated to [^{123}I]Iodometomidate or caused by the addition of a solubilizer (Tween80), which was consequently replaced by ethanol. During long-term follow-up (> 2 years) no side effects attributable to the study were observed. The whole-body effective dose was calculated to be typically less than 3 mSv. Non-German sites will be permitted to use the tracer [^{11}C]Metomidate (MTO) instead of [^{123}I]Iodometomidate (IMTO) if available at the respective site and if in compliance with local regulatory authorities. The rationale is that there is no impact on the qualitative assessment (Visual score) which is the primary endpoint of the study. Whenever quantification (Uptake) is part of a secondary endpoint, a subgroup analysis will be performed.

5.2 Radiolabelling of the tracer and biodistribution

The radiosynthesis of [^{123}I]Iodometomidate is performed by radioiododestannylation using an optimized and validated protocol. The tracer can be obtained within a very short reaction time of 3 min in >90% radiochemical yield. Purification is performed by radio-HPLC followed by formulation in PBS/20%ethanol and sterile filtration.

Labelling of [^{123}I]Iodometomidate for in-house-production will be performed by one study centre (Würzburg) which has access to laboratories meeting GMP standards and holds a manufacturing authorization for [^{123}I]Iodometomidate. The study centre Würzburg plans to start the FAMIAN-Trial as soon as possible.

The remaining study centres will be provided with [^{123}I]Iodometomidate by the company MAP Medical Technologies Oy (Elementtitie 27, FI-41160 Tikkakoski, Finnland).

Additionally, the alternative supply of [^{123}I]Iodometomidate to the study centre Würzburg by MAP Medical will be allowed to bridge temporary shortage of devices (i.e. maintenance) or personnel (holidays, illness).

Vice versa it will be possible for all centers to send their patients to Würzburg solely for the [^{123}I]Iodometomidate scan: In this case the sponsor will provide for a reasonable accommodation for the necessary overnight stay for patient - and partner if requested.

Non-German sites will be permitted to use [^{11}C]Metomidate (MTO) instead if available through in-house-production and after having obtained consent of the local authorities.

5.3 FDG

The radiopharmaceutical [^{18}F]Fluorodeoxyglucose (FDG) has been described already in the late 1970s as surrogate marker for tissue glycycolysis which was subsequently used to non-invasively detect and characterize malignant tumors. The positron emitter ^{18}F enables 3D imaging of the entire body using positron emission tomography (PET). PET, in combination with spiral CT, represents one of the most sensitive and specific imaging modalities and a mainstay diagnostic test for cancer imaging (18).

The German study centres will be provided with FDG from several distributors, which hold a marketing authorization for FDG:

Study centre	Distributor
Würzburg	ZAG Zyklotron AG<br>Hermann-von-Helmholtz-Platz 1<br>76355 Eggenstein-Leopoldshafen
Leipzig	Universitätsklinikum Leipzig AöR<br>Liebigstr. 18<br>04103 Leipzig
Mainz, München	Advanced Accelerator Applications Germany<br>Saime-Genc-Ring 18<br>53121 Bonn
Berlin	Life Radiopharma Berlin GmbH<br>Max-Planck-Str. 4<br>12489 Berlin
Essen	Universitätsklinikum Essen A.d.ö.R.<br>Hufelandstraße 55<br>45122 Essen

6 DIAGNOSTIC STUDIES

6.1. FDG-PET, FDG-PET/CT

In all participating centres, patients will have FDG-PET scans using state-of-the-art hybrid PET/CT devices. Within the FAMIAN Protocol only low dose CT will be performed, however, in case of clinical need, full dose CT can be performed for diagnostic purposes at the discretion of the investigator to avoid additional radiation exposure by a separate imaging procedure. CT data will be also used for attenuation correction and anatomic localization of lesions detected by PET. Patients will fast for at least 6 hours before the PET/CT scan, and blood glucose levels will be measured before the administration of FDG. Blood glucose levels have to be below 150 mg/dl. Static emission imaging will be performed 60 minutes (aimed window: 55 to 75 min, a longer interval due to clinical requirements is acceptable) after i.v.-injection of FDG according to PET/CT scanners and guidelines (e.g. EANM PET/CT: EANM procedure guidelines for tumor imaging: version 2.0; Bundesamt für Strahlenschutz: Bekanntmachung der aktualisierten diagnostischen Referenzwerte für nuklearmedizinische Untersuchungen, 25.Sept.2012). Emission data will be corrected for random, dead time, scatter and attenuation.

6.2 [^{123}I]Iodometomidate-SPECT

[^{123}I]IMTO will be prepared as described above (5.2). Imaging will be performed with state of the art gamma cameras and acquisition settings adequate for ^{123}Iodine. The settings and protocols indicated below for the site Würzburg are exemplary only, comparable protocols will be utilized by participating centres. Thirty minutes before injection of the radiotracer, radioiodine uptake into the thyroid gland will be blocked by oral administration of 600 -1200 mg sodium perchlorate. Thyroid blocking will be continued for 1-3 days using 3 x 460 mg sodium perchlorate per day. For Non-German sites the use of potassium iodide or other perchlorate preparations will be allowed instead of sodium perchlorate. After receiving 185 MBq [^{123}I]IMTO i.v., planar scans of the whole body will be acquired 4 h post injection using a standard technique (Würzburg: Siemens Symbia dual headed large field-of-view gamma camera, energy window 159 keV ± 7,5%, bed speed ("scan velocity") 20 cm/min, matrix 256 x 1024, medium energy parallel-hole collimator). All patients will also receive SPECT/CT imaging between 4 and 6 hours post injection (Würzburg: SPECT/CT will be performed using a Siemens Symbia T2 dual-headed large field-of-view gamma camera combined with a dual-detector CT, energy window of 159 keV ± 7,5%, a rotation of 180° with 3° angular steps (= 2 x 60 frames) at 30 s per projection, 128 x 128 matrix size, slice thickness 4.8 mm, medium energy collimator). Transmission will be measured by low-dose CT (Würzburg: 130 kV and 17 mAs, reconstructed slice thickness 5 mm, B08s kernel for attenuation correction, B30S kernel for visual interpretation and image fusion). SPECT data will be corrected for attenuation and reconstructed iteratively using the OSEM algorithm (Würzburg: 6 subsets, 6 iterations, 3D smoothing 8 mm). As an option, planar scans and SPECT/CT may be repeated 24 h post injection (delayed scan).

6.3 Centralized Imaging Review

The central imaging board is located at the University Hospital Würzburg. A multidisciplinary panel consisting of an endocrinologist, a radiologist and a nuclear medicine physician, accompanied by a statistician, will review all whole-body planar scans, PET, SPECT and CT scans blinded to clinical information. The visual interpretation criteria include a scoring system (1-5). A positive IMTO lesion is defined according to standardized criteria as focal or diffuse uptake in projection of the adrenal tumor higher than surrounding background activity based on planar and SPECT imaging (1: strongly positive, adrenocortical origin; 2: moderately positive, indicative for adrenocortical origin; 3: indeterminate; 4: mildly positive, unlikely adrenocortical origin; 5: negative, no adrenocortical origin).

Regarding FDG-PET scans, intensely FDG-avid lesions are scored as malignant (1), moderately FDG-avid lesions as more likely malignant (2), as indeterminate (3), mildly FDG-avid (4, more likely benign) or negative (5, definitely benign). In addition, semiquantitative analysis of tracer retention will be performed using tomographic images and 5 mm regions of interest for PET or 20 mm regions of interest for SPECT. ROIs are drawn in the area of the hottest pixel.

7 TRIAL PROCEDURE

7.1 Screening

All patients with an indeterminate adrenal mass > 3 cm will be screened for inclusion and exclusion criteria by their physicians. Eligible patients will be handed out a patient information sheet. Imaging data documenting an adrenal tumor according to the inclusion criteria should not be older than 3 months.

7.1.1 Informed Consent

The trial subject will have the opportunity to read the patient information sheet and consider his/her decision before dating and signing the document, and will be given a copy of the signed document. No patient will be able to enter the study before his/her informed consent has been obtained. Patients will be informed that they may withdraw their consent to participate at any time of the trial without giving the reason for it. The patient will be informed that choosing not to participate or to withdraw the consent will not affect his/her subsequent medical treatment or relationship to the treating physician.

7.1.2 Enrolment and screening visit

Once written informed consent has been obtained, the patient will be enrolled in the study (**Visit 1**). Baseline evaluations should be performed within 4 weeks after inclusion (i.e. written informed consent). Screening and baseline evaluations include:

- Patient informed consent prior to any study related procedures
- Documentation of height and weight
- Documentation of detailed medical history
- Documentation of current medication
- Routine laboratory tests (serum creatinine, serum urea, glomerular filtration rate (MDRD), blood count, serum aspartate aminotranferase, alanin aminotranferase, alkaline phophatase, gamma glutamyl transferase). If laboratory tests have been performed within two weeks of screening they do not have to be repeated.
- Evaluation of inclusion and exclusion criteria
- Recovery of imaging slides
- Physical examination
- Documentation of historical hormonal assessment (*Table 1*)

- 24 hours urine collection for steroidobolomics (not relevant for inclusion/exclusion of patients): If a sample of a 24h urine collected no earlier than 3 months before inclusion is stored at site (i.e. for Ens@t) then a portion of this can be sent.
- Pregnancy test (in women of child bearing potential): Any pregnancy test is allowed. However, sponsor will provide urinary pregnancy tests for the convenience of the sites.

For the safety of the patients, there will be no exceptions to eligibility requirements at the time of entering the diagnostic phase of the study.

Table 1: Hormonal Assessment (acceptable timeframe of previous blood draws)

Measurement	Mandatory	Optional	Timeframe
Plasma normetanephrines and metanephrines<br>OR<br>urine normetanephrines and metanephrines	Exclusion Criterion 3		If **<** ULN, measurement up to 12 months before screening will be accepted if the adrenal tumor has already been diagnosed at that time and measurement was done for hormonal evaluation of the respective adrenal tumor<br>If **>** ULN, measurement should be no older than 3 months before screening.
Plasma/Serum Aldosterone<br>**AND**<br>Plasma renin concen-tration<br>**OR**<br>Plasma renin activity	Exclusion Criterion 4		If plasma/serum aldosterone concentration (ng/l) to plasma renin concentration (ng/l) ratio (or equivalent) **<** cutoff for the respective laboratory, measurement up to 12 months before screening will be accepted if the adrenal tumor has already been diagnosed at that time and measurement was done for hormonal evaluation of the respective adrenal tumor.<br>If ARR **>** cutoff for the respective laboratory, measurement should be no older than 3 months before screening.

Dexa Suppression Test (morning serum cortisol after 1 mg dexamethasone at 23.00 h)	(Exclusion Criterion 5)	One of the tests is mandatory (which one is at the discretion of the investigator). If the test performed does not fulfill the exclusion criteria no further tests are necessary.	If **<** 2.5 µg/dl, measurement up to 12 months before screening will be accepted if the adrenal tumor has already been diagnosed at that time and measurement was done for hormonal evaluation of the respective adrenal tumor.<br>If **>** 2.5 µg/dl, measurement should be no older than 3 months before screening.
Plasma ACTH	(Exclusion Criterion 5)		Not older than 3 months before screening.
Urinary free cortisol<br>**OR**<br>bed time (23.00 h) salivary cortisol	(Exclusion Criterion 5)		Not older than 3 months if the adrenal tumor has already been diagnosed at that time and measurement was done for hormonal evaluation of the respective adrenal tumor.
DHEAS<br>17-OH-Progesterone		Optional	Up to 12 months before screening if the adrenal tumor has already been diagnosed at that time and measurement was done for hormonal evaluation of the respective adrenal tumor.

The documentation of the hormonal assessment is adapted from the ENS@T criteria: Study sites are highly encouraged to follow these.

7.2 Diagnostic Phase

Upon completion of all screening tests and confirmation of eligibility, patients will be included in the diagnostic phase of the study. Diagnostic evaluation (**Visit 2 and 3**) will start within four weeks after screening.

Patients will be evaluated by both [^{123}I]Iodometomidate SPECT/CT and [^{18}F]Fluorodeoxyglucose PET/CT (see standard operating procedures in the Appendix). Computed tomography will be performed as low dose CT of the adrenal region to reduce radiation exposure. Imaging can be started with either [^{123}I]Iodometomidate SPECT/CT or [^{18}F]Fluorodeoxyglucose and can be performed on two consecutive days. The time interval between [^{123}I]Iodometomidate SPECT/CT and [^{18}F]Fluorodeoxyglucose should not exceed 8 weeks if both imaging is done within the study.

If [^{18}F]Fluorodeoxyglucose PET has already been performed prior to inclusion in the study only [^{123}I]Iodometomidate SPECT/CT will be performed and [^{18}F]Fluorodeoxyglucose PET results will be included in the final analysis if FDG-PET has been performed at maximum three months before study inclusion and if the PET was performed in accordance with usual standard of care.

After the completion of imaging, the patient will enter the follow-up phase.

7.3 Follow-up phase

Patients will have a follow up visit 2-4 weeks after [^{123}I]Iodometomidate SPECT/CT and [^{18}F]Fluorodeoxyglucose PET/CT - or immediately before surgery (within the preoperative routine will suffice), if surgery performed within 2 weeks post imaging. (**Visit 4**). Routine laboratory tests will be repeated:

Serum-creatinine, urea, glomerular filtration rate (MDRD), blood count, aspartate aminotransferase, alanin aminotransferase, alkaline phophatase, and gamma glutamyl transferase.

Patients will further be interviewed regarding possible adverse events due to imaging.

7.4 Study Duration

Study duration for each patient is the time from inclusion to **Visit 4** (Follow up).

In case of refusal of surgery, investigators are highly encouraged to follow the current guidelines on management of incidentalomas that suggest a follow up after 6 -12 months - including follow up imaging by either computed tomography or MRI (19). If imaging is performed – not as study procedure but at the discretion of the investigator – imaging data should be recorded in the CRF (**Visit 5**).

7.5 Subject discontinuation

The following events are considered as reasons for discontinuing a subject from the study:

- Adverse Event(s) with failure to recover
- Withdrawal of consent
- Pregnancy
- Death
- Protocol violation

The reason for discontinuation should be recorded in the CRF and in the source documents.

7.6 Table of Events or Visit Schedule and Assessments

Table 2 lists all of the assessments and indicates with "X" the visits when they are performed. All data obtained from these assessments must be supported in the patient's source documentation.

7.6.1 Screening failure

Patients who complete the informed consent process and do not meet all entry criteria and therefore are not eligible for inclusion will be considered as screening failures. Patient's characteristics such as age, gender and reason of screening failure will be described.

Table 2: Visit Plan/Schedule of Assessments

Events	Screening Period	Diagnostic Period		Follow-up Period	
	**Visit 1**	**Visit 2**	**Visit 3**	**Visit 4**	**(Visit 5[e])**
Timepoint	*Day 0*	*≤ 4 weeks after V1*	*≤ 8 weeks after V2*	*2-4 weeks after V3 or preoperative surgical routine – whichever comes first*	*6-12 months after V3*<br><br>*Only in case that surgery is not performed*
Informed Consent	X				
Inclusion/Exclusion Criteria	X				
Medical History	X				
Current Medication	X				
Recovery of imaging slides	X				
Routine Blood Tests [a]	X			X	
Review of hormonal assessment according to ENSAT criteria [b]	X				
Pregnancy Test in women of childbearing potential [c]	X	(X)	(X)		
Physical Examination, Height, Weight/BSA	X				
Adverse Event / Serious Adverse Event [d]	X	X	X	X	
24 hour urine	X				
Follow up imaging CT or MRI of adrenal region [e]					(X)
[^{18}F]Flurodeoxyglucose PET/CT		X (if not at visit 3)	X (if not at visit 2)		
[123I]Iodometomidate SPECT/CT		X (if not at visit 3)	X (if not at visit 2)		

a Routine blood tests: Serum-creatinine, urea, glomerular filtration rate (MDRD), blood count (haemoglobin, leukocytes, platelets), aspartate aminotranferase, alanin aminotranferase, alkaline phophatase, and gamma glutamyl transferase. If done within 2 weeks before V1, then no need to repeat.

b Hormonal assessment: Morning serum cortisol after 1 mg dexamethasone 23:00, (urinary free cortisol), (midnight salivary cortisol), baseline plasma ACTH, plasma renin concentration or plasma renin activity, serum aldosterone, (DHEAS), (17-OH-progesterone) , plasma metanephrines/plasma normetanephrines or urinary metanephrine/normetanephrine excretion (*Table 1*)

c At the discretion of investigator. This should follow usual care at the respective clinic of nuclear medicine, since pregnancy should be ruled out for almost all routine procedures: If in routine care this is only done by questioning then this should suffice.

d AEs will be collected from inclusion of study to visit 4.

e Only in case of refusal of surgery, follow up imaging (CT or MRI) will be performed 6-12 months after inclusion into the study and at the discretion of the attending physician

7.6.2 Safety Assessements

All subjects will have a physical examination at screening.

Routine blood tests: Serum-creatinine, urea, glomerular filtration rate (MDRD), blood count (haemoglobin, leukocytes, platelets), aspartate aminotranferase, alanin aminotranferase, alkaline phophatase, and gamma glutamyl transferase will be performed at Visit 1 and Visit 4 (2-4 weeks after imaging). Adverse events will be assessed at every study visit. NCI-CTCAE v.4 will be used to report toxicity

(https://evs.nci.nih.gov/ftp1/CTCAE/CTCAE_4.03/CTCAE_4.03_2010-06-14_QuickReference_5x7.pdf)

7.7 Violation of eligibility criteria

If a patient has been included into the study and it turns out that he/she did not fit into the eligibility criteria, the following procedures will be followed: In case the patient has not yet been imaged, his/her participation in the trial is stopped entirely. If the patient has already undergone imaging, immediate contact with the centre for clinical study at the University of Würzburg and the coordinating investigator/the sponsor is indicated. It is mandatory to stop participating in the trial immediately in case the non-eligible patient has undergone only one imaging procedure or in case the patient has been included by error without his/her consent. In all other cases, proceed per protocol and wait for instructions. The steering committee will in each individual case discuss with the investigator whether maintenance within the FAMIAN trial is indicated. Documentation of the patient’s clinical data will be continued throughout the entire trial.

8 BIOMETRICAL ASPECTS

The study aims to evaluate the utility of combined FDG-PET and [^{123}I]Iodometomidate imaging for non-invasive characterization of indeterminate adrenal neoplasias. The primary objective is to demonstrate effectiveness of a test for benign adrenocortical adenomas (AA). A secondary focus is on the performance of the diagnostic AA test given ACC or non-ACC.

8.1 Outcome variables and measures

As this is a diagnostic study, the results of histopathology of surgical specimens as assessed by an expert pathologist will serve as the comparator (gold standard) to the results of imaging. Test performance will be assessed among participants with (sensitivity) and without (specificity) adrenocortical adenoma, as a hormonally silent adenoma does not require surgery.

8.1.1 Primary variables and measures

Gold standard: An expert pathologist will classify the incidentaloma of a patient based on histopathology of surgical specimens. All removed neoplasias will be classified according to histopathological criteria. Primary classification will be all neoplasias AA or at least one neoplasia non-AA.

Diagnostic tests: The tumors will be characterized as either positive or negative concerning FDG/Iodometomidate uptake leading to 4 different categories. The primary diagnostic test variables subsume to the indicators “all neoplasias of a patient FDG-PET negative” and “all neoplasias of a patient Iodometomidate positive”.

The diagnostic measures of primary interest are specificity, sensitivity and likelihood ratio of a positive diagnostic AA test using histopathology of the surgically removed tumor as gold standard for the differential diagnosis. Thereby the AA test uses the combined FDG-PET and Iodometomidate imaging: AA+ is defined for each neoplasia as FDG-PET negative and Iodometomidate positive. The primary clinical outcome is the correct non-invasive diagnosis (using the combined AA test) of histologically verified adrenocortical adenoma and verified neoplasia different from adrenocortical adenoma (for the estimation of the sensitivity and the specificity of the combined AA test).

8.1.2 Secondary variables and measures

Gold standard: An expert pathologist will classify the incidentaloma of a patient based on histopathology of surgical specimens. All removed neoplasias will be classified according to histopathological criteria. Each neoplasia will be classified as AA, ACC or another benign or malignant adrenal incidentaloma.

Usually, the gold standard diagnosis can be subsumed on a patient level. If in rare cases combinations occur, analyses may in addition be performed on a neoplasia level.

Key secondary outcome measures are the likelihood ratio of a negative diagnostic AA test a priori probabilities of AA, ACC and other benign as well as malignant adrenal incidentalomas and positive and negative predictive values.

8.1.3 Exploratory endpoints

In addition, a cost effectiveness analysis will be performed comparing imaging costs with potentially avoidable costs for surgery.

Steroid profiling analysis will be used to compare the urinary steroid profile in malignant adrenal tumors with that in benign adenomas and non-adrenal lesions to assess the role of steroidobolomics for non-invasive diagnosis of adrenocortical cancer.

8.2 Statistical hypothesis and Confirmatory Statistical Analysis

The confirmatory analysis consists primary of testing two hypotheses hierarchically at a two-sided type I error level of alpha=5%.

In a first test, for the diagnostic AA test a specificity (probability of AA- given non-AA, i.e. the / at least one patient's neoplasia is not a benign adrenocortical adenoma) of more than 90% should be demonstrated. In case of significance, in a second test it should be shown that the likelihood ratio (AA relative to non-AA) of a positive AA test (AA+) is greater than 9.

If also the second test is significant, under the accepted assumption that a priori (assessment before combined imaging and without gold standard) the probability of a benign adrenocortical adenoma (AA, all patients' neoplasias) is at least 50%, it can be statistically concluded that the positive predictive value of the diagnostic AA test (probability of a benign adrenocortical carcinoma given AA+) is greater than 90%.

An exact binomial test will be performed for testing for the first alternative hypothesis. Where required to improve efficiency, two stage designs will be specified (based on gold standard, blinded regarding diagnostic test).

Specificity and sensitivity of the AA test will be estimated with 95% confidence intervals. At least the method of Clopper and Pearson will be adopted, if possible the more efficient method of Blyth, Still and Casella will be utilized (usually in the one stage binomial test design).

The second alternative will be tested using an approximately normally distributed test statistic based on the estimators of the sensitivity and the specificity of the diagnostic AA test.

Two further statistical tests are planned in a hierarchical order at a two-sided type I error level of alpha=0.05 (confirmatory in the case of significance of the first two statistical tests, otherwise explorative):

The third test will examine the alternative hypothesis of a probability of a negative diagnostic AA test (AA-) given ACC of more than 80%.

The fourth test will examine the alternative hypothesis of a likelihood ratio (ACC relative to non-ACC) of a positive AA test (AA+) less than 7/19.

In the case of significance, under the accepted assumption that the probability of ACC is at most 12.5%, it can be statistically concluded that the probability of ACC given AA+ is less than 5%. Statistical tests and rate estimation are performed in a similar way as described before.

The analyses are conducted primarily in the spirit of the intention to treat principle (ITT), meaning that all patients with assessable endpoints will be included in the analyses regardless of violation of eligibility criteria.

8.3 Proposed sample size / power calculations

Sample size calculation is based on the two primary hierarchically structured two-sided level 5% tests.

For the first test, assuming an a priori probability of 60% for AA and a specificity (P(AA-|non-AA)) of 98% to be detected with a power of 80%, approximately 195 assessed patients are necessary. For the exact test, at least 70 patients with a confirmed non-AA neoplasia are necessary.

For the second test, assuming an a priori probability of 60% for AA and a specificity of 98% together with a sensitivity (P(AA+|AA)) of 92% to be detected with a power of 80%, approximately 187 assessed patients are necessary.

Thus 195 patients to be assessed are necessary. Adjusting for incomplete assessments in up to 10% of patients and a recruitment rate of 40% within screened patients, 220 patients have to be recruited and 550 to be screened.

For the third test, assuming an a priori probability of 10% for ACC (~20 ACC patients), the power to detect P(AA-|ACC)=98% is ~70%. For the fourth test, assuming a priori probabilities of 60% for AA and 10% for ACC, the power to detect P(AA-|ACC)=98% together with P(AA+|non-ACC)=61.5% is almost 80%.

The assumptions on a priori probabilities and detectable probabilities to be powered were set up in consensus at the ENSAT Cancer Imaging Study Group meeting at the 1st European Conference on Adrenal Imaging 2011.

8.4 Cost effectiveness analysis / Steroidobolomics

Economic analyses will be performed from a payer's perspective. Costs for diagnostic tests ([^{18}F]FDG-PET, [^{123}I]Iodometomidate), costs for non-surgical management and follow-up and costs for surgical procedures will be calculated according to the base rate of the University Hospital Würzburg and to the

national frequencies of relevant dose-related groups (DRGs). The incremental cost effectiveness ratio (ICER) per correctly assigned benign or malignant incidentaloma will be assessed for the individual imaging tests and the combination of both.

In addition to the investigations described above, all participants will be asked to collect a 24-h urine at baseline that will be subjected to steroid profiling by gas chromatography/mass spectrometry followed by machine-learning based computational analysis of results. This urine steroid metabolomics approach for differentiating benign from malignant adrenocortical tumors has been recently described (Arlt W et al., J Clin Endocrinol Metab 2011) and results will be compared accordingly with the results of Iodometomidate and FDG-PET prediction of malignancy.

8.5 Background and demographic characteristics

Background and demographic characteristics as well as clinical characteristics will be analysed using standard descriptive techniques, e.g. tables of absolute and relative frequencies, means and standard deviations, median, quartiles, minimum and maximum.

8.6 Safety analysis

Standard routine parameter changes from before imaging to prior to surgery will be assessed. Adverse events will be assessed from registration until end of follow up which is Visit 4. Surgery or biopsy of the adrenal lesion(s) is expected within the range of 1-6 weeks after the second imaging. Adverse and serious adverse events will be presented using standard descriptive statistical methods.

9 ADVERSE EVENTS AND SERIOUS ADVERSE EVENTS

9.1 Definition

9.1.1 Adverse Event (AE)

An Adverse Event (AE) is any new untoward medical occurrence or worsening of a pre-existing medical condition in a patient or clinical investigation subject administered an investigational (medicinal) product. An AE can therefore be any unfavourable and unintended sign (including abnormal laboratory findings for example), symptom, or disease temporally associated with the use of a medical product, whether or not a causal relationship (i.e. related/not related) with the treatment is suspected.

9.1.2 Serious Adverse Event (SAE)

A Serious Adverse Event (SAE) is any untoward medical occurrence that at any dose:

- is fatal (results in death)
- is life-threatening
- requires or prolongs in-patient hospitalization
- results in persistent or significant disability / incapacity
- is a congenital anomaly / birth defect
- is medically significant (defined as any clinical event or laboratory result that may not be immediately life-threatening or result in death or hospitalization but, based upon appropriate medical and scientific judgment, may jeopardize the subject or may require intervention (e.g. medical, surgical) to prevent one of the other serious outcomes listed in the definition above. Examples of such events include but are not limited to, allergic bronchospasm requiring intensive treatment in an emergency room or at home, blood dyscrasia or convulsions that do not result in inpatient hospitalization, development of drug dependency or drug abuse)

A SAE judged as potentially related to a study drug qualifies as Serious Adverse Drug Reaction (SADR).

Events exclusively related to tumor relapse/progression or treatment of tumor relapse/ progression are not considered SAEs.

NOTE:

The following hospitalizations are **not** considered SAEs:

- A visit to the emergency room or other hospital department for less than 24 hours that does not result in admission (unless considered a "medically significant event" or a "life-threatening event")
- Outpatient or same-day or ambulatory procedures
- Observation on short-stay units
- Hospitalization due to diagnostic procedures or standard supportive care (e.g. implant of central venous catheter, routine treatment or monitoring of the studied indication not associated with any deterioration in condition).
- The administration of blood or platelet transfusion as routine treatment of studied indication. However, hospitalization or prolonged hospitalization for a complication of such transfusion remains a reportable SAE.
- A pre-planned hospitalization for a condition which existed at the start of study drug and which did not worsen during the course of study drug treatment (e. g. scans, endoscopy, sampling for laboratory tests…). However, hospitalization or prolonged hospitalization for a complication of such procedures remains a reportable SAE.
- Social admission (e.g. subject has no place to sleep; hospice facilities)
- Administrative admission (e.g. for yearly physical examinations)
- Protocol-specified admission during a clinical trial (e.g. for a procedure required by the study protocol or for clinical research)
- Optional admission not associated with a precipitating clinical AE (e.g. for elective cosmetic surgery)

9.2 Exposure to imaging during pregnancy/lactation

In principle, pregnancy and lactation period are exclusion criteria. In the event of a pregnancy occurring during the course of the study, the subject must be withdrawn from the study immediately. The Safety Office unit must be notified within 24 hours if a pregnancy occurs during the study or within 30 days following the last administration of study treatment and the subject has to be followed during the entire course of pregnancy and postpartum period. Maternal and neonatal outcomes must be recorded even if they are completely normal and without AEs. The "Pregnancy report form" should be used.

9.3 Recording of adverse events

9.3.1 When to collect AEs

Any AE that occurs from the time consent is given to follow-up (V4) should be recorded.

All subjects should be monitored for AEs during the study. Assessments may include monitoring of any or all of the following parameters: the subject's clinical symptoms, laboratory, pathological, radiological or surgical findings, physical examination findings, or other appropriate tests and procedures.

9.3.2 Adverse Event (AE) and Serious Adverse Event (SAE) Documentation and Reporting

All observed AE regardless of treatment group or suspected causal relationship to study drug will be assessed following NCI-CTCAE version 4.0 and recorded on the AE page of the CRF, and in the case of a serious adverse event will also be recorded on a SAE form.

All SAEs must be reported immediately (i.e., within 24 hours of the Investigator's knowledge of the event) by facsimile of the SAE Report Form to the Würzburg Study Centre (Endocrinological Trial Unit):

Universitätsklinikum Würzburg

Medizinische Klinik und Poliklinik I

Endokrinologische Studienambulanz

Oberdürrbacher Str. 6

97080 Würzburg, Germany

Phone: +49-931-201-39716

Fax: +49-931-201-6039716

E-mail: famian@ukw.de

A diagnosis or syndrome should be recorded on the AE page of the CRF rather than the individual signs or symptoms of the diagnosis or syndrome.

An overdose, accidental or intentional, whether or not it is associated with an AE, or abuse, withdrawal, sensitivity or toxicity to an investigational medicinal product should be reported as an AE. If an overdose is associated with an AE, the overdose and adverse event should be reported as separate terms.

In the event of overdose or exaggerated response, the subject should be monitored as appropriate and should receive supportive measures as necessary. There is no known specific antidote for overdose. Actual treatment should depend on the severity of the clinical situation and the judgment and experience of the treating physician.

Worsening/exacerbation of signs and symptoms (in terms of severity and/or frequency, or the appearance of new manifestations/complications) of the neoplasia under study or of a pre-existing illness should be reported as an AE in the appropriate section of the CRF – but are not considered as SAEs (refer to 9.1.2 Serious Adverse Events).

In addition, clinically significant changes in physical examination findings and abnormal objective test findings (e.g., X-ray, ECG) should also be recorded as AEs. The criteria for determining whether an abnormal objective test finding should be reported as an AE are as follows:

Test result leads to a change in study dosing or discontinuation from the study, significant additional concomitant drug treatment or other therapy, and/or test result leads to any of the outcomes included in the definition of a SAE, and/or test result is considered to be an AE by the investigator or sponsor.

If an abnormal laboratory value is associated with a diagnosis or clinical sign/symptom, the diagnosis or sign/symptom should be reported as an AE and the associated laboratory result should be considered additional information to support the diagnosis.

For all AEs, the investigator must pursue and obtain adequate information both to determine the outcome of the AE and to assess whether it meets the criteria for classification as a SAE requiring immediate notification to the Würzburg Study Center (Endocrinological Trial Unit). Sufficient information should be obtained by the investigator to determine the causality of the AE (i.e., study drug or other illness). The investigator is required to assess causality and indicate that assessment on the CRF. All AEs and especially those that are serious, suspected to be related to study drug or considered significant by the investigator or clinical monitor must be followed after the time of therapy discontinuation until the event or its sequelae resolve or stabilize at a level acceptable to the investigator and the clinical monitor or his/her designated representative. All AEs will be recorded in the CRF.

9.3.3 Severity assessment

For both AEs and SAEs, the Investigator must assess the severity of the event.
The severity of AEs will be graded based upon the subject's symptoms according to National Cancer Institute (NCI) Common Terminology Criteria for Adverse Events (CTCAE, Version 4.0); http://ctep.cancer.gov/reporting/ctc.html.
AEs that are not defined in the NCI CTCAE should be evaluated for severity according to the following scale:

- **Grade 1 = Mild** – transient or mild discomfort; no limitation in activity; no medical intervention/therapy required
- **Grade 2 = Moderate –** mild to moderate limitation in activity, some assistance may be needed; no or minimal medical intervention/therapy required
- **Grade 3 = Severe** – marked limitation in activity, some assistance usually required; medical intervention/therapy required, hospitalization is possible
- **Grade 4 = Life threatening** – extreme limitation in activity, significant assistance required; significant medical intervention/therapy required, hospitalization or hospice care probable
- **Grade 5 = Death** – the event results in death

9.3.4 Causality assessment

The Investigator must determine the relationship between the administration of IMP and the occurrence of an AE/SAE as not related or related as defined below:

**Not related:**	The temporal relationship of the adverse event to IMP administration makes **a causal relationship unlikely or remote**, or other medications, therapeutic interventions, or underlying conditions provide a sufficient explanation for the observed event.
**Related:**	The temporal relationship of the adverse event to IMP administration makes **a causal relationship possible**, and other medications, therapeutic interventions, or underlying conditions do not provide a sufficient explanation for the observed event.

If an event is assessed as suspected of being related to a comparator, ancillary or additional IMP that has not been manufactured or provided by the sponsor, please provide the name of the manufacturer when reporting the event.

9.3.5 Duration

The Investigator will provide a record of the start and stop dates of the AE/SAE. The duration of the AE and the SAE may vary within one event. For example, a non-serious AE may begin on 01-Jan. The event will become serious when it meets one of the criteria for seriousness (e.g., the subject is hospitalized on 05-Jan). The SAE will continue until it no longer meets the seriousness criteria (e.g., the subject is discharged on 07-Jan). However, the AE continues until 10-Jan when the event resolves. The AE dates may extend from before and beyond the SAE dates, but not the reverse.

9.3.6 Action Taken

The Investigator will report the discontinuation or reduction of IMP following an AE and report if concomitant and/or additional treatments were given for the AE.

9.3.7 Outcome

All SAEs that have not resolved upon discontinuation of the subject’s participation in the study must be followed until recovered, recovered with sequelae, not recovered (death due to another cause) or death (due to the SAE).

9.4 Expedited Reporting of Adverse Events

All Serious Adverse Events (SAE), related or not to study drugs, occurring at any time during the study – from date of informed consent to V4 (follow up) - independent of the circumstances or suspected cause, must be reported, within 24 hours of knowledge by fax.

The investigator must fill in the SAE Form in English and assess the relationship to the IMP, and send it signed and dated, within 24 hours of learning of its occurrence, even if it does not appear to be treatment-related, to the Würzburg Study Center (Endocrinological Trial Unit) at the University Hospital of Würzburg.

Any late Serious Adverse Drug Reaction (SADR), occurring after the Follow-up period also must be reported to the Würzburg Study Center (Endocrinological Trial Unit) at the University Hospital of Würzburg.

Information collected in the SAE form is crucial to assess the case. For this reason, diligences in collecting as much verifiable and reliable information are needed: both, quality and timeliness are key factors. If known, the diagnosis of the underlying illness or disorder should be recorded, rather than its individual symptoms. The following information should be captured for all SAEs: onset, duration, severity, seriousness, relationship to study drugs, action taken and treatment required.

The investigator must also attach the following whenever possible:

- a copy of the summary of hospitalization or prolongation of hospitalization
- a copy of the post-mortem report (if applicable)
- a copy of all relevant laboratory examinations and the dates on which these examinations were carried out, including relevant negative results as well as normal laboratory ranges
- all other documents that are judged useful and relevant

All these documents will remain pseudonymized.

Follow-up information

The investigator is responsible for the appropriate medical follow-up of patients until resolution or stabilization of the adverse event or until the patient's death. This may mean that follow-up should continue once the patient has left the trial. Follow up information about a previously reported SAE must be reported by the investigator to the Endocrinological Trial Unit **within 24 hours** of receiving it (at latest on the next working day), on the serious adverse event report form, by ticking the box marked “Follow-up N°…”. The investigator also transmits the final report at the time of resolution or stabilization of the SAE. The investigator retains the documents concerning the supposed serious adverse event so that previously transmitted information can be completed if necessary.

9.5 Responsibilities of the Coordinating Investigator

Suspected Unexpected Serious Adverse Reactions (SUSARs)

9.5.1 Definition of SUSARs

Suspected Unexpected Serious Adverse Drug Reactions are side effects (probably or definitely connected with the administration of the investigational product), the nature or severity of which are inconsistent with the information available about the product. Information about the investigational product is contained in the Investigational Medicinal Product Dossier/Summary of Product Characteristics.

9.5.2 Documentation and Reporting

The Würzburg Study Center (Endocrinological Trial Unit) on behalf of the sponsor submits all information available about SUSAR immediately, latest within 15 days after the event becomes known to the leading ethics committee, the competent regulatory authority (BfArM), national coordinating investigator and to all participating investigators.

The Würzburg Study Center (Endocrinological Trial Unit) will also send to each national coordinating investigator a quarterly summary table of the SAE not considered related to IMP.

9.5.3 Annual safety report

The Würzburg Study Center (Endocrinological Trial Unit) will issue an annual safety report (ASR) which will be made available to the investigators throughout the entire duration of the clinical trial.

Each national coordinator will have the responsibility to submit the ASR within 60 days following the data lock (date of the first authorisation of the concerned clinical trial by a competent authority in a member state) to the national competent authority and the national Ethic Committee of the concerned Member States. This procedure should be conducted in accordance with national legislation.

10 END OF STUDY

Stopping Rules

For individual patients: Participation of patients is fully voluntarily. Patients are able to stop participation in the study at any point in time. In addition, patients can claim the elimination of all data and material at any point in time before anonymization.

For the whole study: If relevant risks to participants related to the diagnostic procedures unexpectedly emerge during this trial, the trial will be stopped in accordance with the data safety monitoring board.

11 COMMITTEES AND PANEL REVIEW

11.1 Steering Committee

The steering committee (A. Buck, F. Beuschlein, M. Gurnell, S. Hahner, F. Tenenbaum, H. Timmers, M. Mannelli) will meet regularly as appropriate to confirm the progression of the study. Once a year, the statistician will give an overview of the study including the accrual rate and toxicity data.

11.2 Independent Data Monitoring Committee

An Independent Data Monitoring Committee (IDMC) composed of 3 international experts: Prof. U. Mansmann (Institut für Medizinische Informationsverarbeitung, Biometrie und Epidemiologie (IBE), Ludwig-Maximilians-Universität München), Prof. M. Schwaiger (Department of Nuclear Medicine, Klinikum rechts der Isar, Technische Universität München), and Prof. B. Böhm (LKC Medicine, NTU and Imperial College London, Singapore) - including one statistician - will monitor the progress of the study on ethical and scientific grounds. The Committee will meet approximately every 12 months (by meeting or conference call).

The role of the IDMC will be:

a) To review accrual rate

b) To monitor toxicity

Every 12 months the sponsor will circulate a report to the members of the IDMC about toxicity (Listing of Adverse Events, SAE reports). The IDMC will review these interim toxicity data although this is primarily the responsibility of the steering committee. This annual procedure prevents problems of major toxicity.

c) To examine first stage analyses

These interim analyses will remain confidential.

On the basis of these analyses, the IDMC may recommend to the sponsor whether the study should continue or whether it should be changed or terminated prematurely. The sponsor will take the final decision.

d) To examine other trials

The IDMC will review reports of related studies performed by other groups or organisations to determine whether such information materially affects the aims or preliminary findings of the trial.

e) Other

The IDMC may be asked to review a major modification to the study prior to its implementation as a study amendment.

12 ETHICAL AND REGULATORY ASPECTS

Our clinical trial is performed according to the legal regulations of the drug act and is carried out in accordance with the guidelines of ICH-GCP (International Conference on Harmonisation of Technical Requirements for the Registration of Pharmaceuticals for Human use - Guideline for Good Clinical Practice of January 17, 1997), as well as national, European and international legal regulations like guidelines of the European Union (EU), guidelines of the Food and Drug Administration (FDA), guidelines of Good Clinical Practice of clinical studies of drugs in human beings (GCP-Verordnung, GCP-V) of April 9, 2004 and the amendment of November 3, 2006 as well as according to the recommendations of World Medical Association (WMA) in the Declaration of Helsinki. The GCP-regulations define all tasks, areas of responsibility, and procedures for the planning, authorisation, application, and surveillance of trials, as well as their documentation and corresponding reporting.

Prior to submission of the trial related documents to the leading ethics committee and to the competent federal authority, the sponsor is responsible for entering the trial into the European database of clinical trials (EudraCT). After the web based entry the sponsor will be issued an EudraCT number which must be submitted with all future documents.

Afterwards, the protocol and all other associated documents according to the German GCP-V § 7 will be submitted to the leading ethics committee responsible for the coordinating investigator for approval. In addition to the required documents from each investigator the coordinating investigator must provide evidence of at least two years of experience in clinical trials.

Parallel to the submission to the leading ethics committee each participating ethics committee also receives a copy of all submitted documents including information about trial sites and investigators (see

above) in their field of responsibility. This documentation should be used by the ethics committee for evaluation of the appropriateness of the trial site. At the same time the study documents will be submitted to the competent federal authority (BfArM) according to the requirements of legal regulations.

Only following a positive review by the leading ethics committee and approval from the competent federal authority the trial can start.

The written approval of the ethics committee must be filed in the trial master file (TMF). Additionally, every participating centre must receive a copy of these documents to be filed in the investigator site file (ISF).

12.1 Sponsor obligations

The Sponsor explicitly delegated the supervision for the European countries to the Coordinating Investigator. In each participating European country a National Coordinator (NC) will be identified. Each NC will take care of its own regulatory submissions and its own safety surveillance. The NCs will be in charge of the patient information notice translation, insurance contracting or indemnity of the included patients in accordance with the applicable regulatory requirements. All documents and protocol amendments must be agreed upon by the Sponsor or the delegated Coordinating Investigator and must be written with input from the investigators.

The Sponsor and each investigational site must archive the entire documents for 15 years.

An agreement will be signed between the Sponsor and each participating site with the list of the responsibilities for each party.

The sponsor is responsible for securing agreement from all involved parties to ensure direct access to all trial related sites, source data/documents, and reports for the purpose of monitoring and auditing by the sponsor, and inspection by domestic and foreign regulatory authorities.

12.2 Information and Consent of Participants

Prior to the conduct of any procedure linked to biomedical research, any person wishing to participate in a research study gives his/her free written informed consent. This is only obtained after the participant has been informed thoroughly by the investigator during a consultation and has been given sufficient time to think his/her decision over.

Having read the patient information, the patient must date and sign the **consent form** if he/she accepts to participate. This consent form must also be signed by the investigator. The original consent form must be kept in the study file by the investigator and the study participant should receive a copy.

12.3 Investigator Responsibilities

Investigator responsibilities are set out in the ICH Guideline for Good Clinical Practice and in the local regulations. The PI/Investigator should ensure that all persons assisting with the study are adequately informed about the protocol, amendments, study treatments, as well as study-related duties and functions. The Investigator should maintain a list of Investigators/Sub-Investigators and other appropriately qualified persons to whom he or she has delegated significant study-related duties.

The PI/Investigator is responsible for keeping a record of all subjects who sign an informed consent document and are screened for entry into the study. Subjects who fail screening must have the reason(s) recorded in the subject's source documents.

The Investigator, or a designated member of the Investigator's staff, must be available during monitoring visits to review data, resolve queries and allow direct access to subject records (e.g., medical records, office charts, hospital charts, and study-related charts) for source data verification. The Investigator must ensure timely and accurate completion of CRFs and queries.

The participating sites have to agree to regulatory inspection and Sponsor audits.

13 DATA COLLECTION

Since the planned eCRF could not be realized, data will be captured on paper CRF.

14 QUALITY ASSURANCE - MONITORING

Quality control and assurance checks will be performed by the sponsor. The trial will be conducted according to national regulations and according to quality and conduct standard procedures of the Clinical Trial Centre at the University Hospital of Würzburg (ZKSW, Zentrale für Klinische Studien Würzburg). The investigator is required to permit direct access to the facilities where the study took place, source documents, CRFs and applicable supporting records of study subject participation for audits and inspections by IRB/IECs, regulatory authorities and sponsor authorized representatives.

In order to guarantee the authenticity and the credibility of the data in conformity with good clinical practices, the Sponsor has installed a quality assurance system in accordance with national and international regulations which includes Trial Management, Quality Control of data at the investigating site by Clinical Research Associate (CRA) and Quality Assurance through voluntary audits of the investigating centres.

Quality control on the site will be ensured by the Sponsor/Co-Sponsor's CRA. The CRA must check that the investigator's file exists and that it is updated, must verify the consent forms, that subjects fulfil

eligibility criteria, the validity of evaluation criteria and treatment toxicity with the help of source documents. The CRA will check drug accountability and ensure that the drug accountability forms are validated and signed by the in-house radiochemist before any request for destruction.

The responsibilities of the CRA, the frequency of visits and the depth of source data validation are documented in the monitor manual.

15 DATA OWNERSHIP / PUBLICATION POLICY

The investigator promises, on his/her behalf as well as that of all the persons involved in the conduct of the trial, to guarantee the confidentiality of all the information which is provided in the context of the trial by the respective investigations until the publication of the results of the trial.

All publications, abstracts or presentations including the results of the trial require prior approval of the Sponsor.

All oral presentations, manuscripts must include a rubric mentioning the Sponsor, the investigators / institutions that participated in the trial, the cooperative groups, learned societies which contributed to the conduct of the trial and the bodies which funded the research.

The Coordinating Investigator will write an article reporting on the results as soon as possible after the final analysis and will be the first author of the publication.

The results of this study should be published in a medical publication journal, and may be used for teaching purposes. Additionally, this study and its results may be submitted for inclusion in all appropriate health authority study registries, as well as publication on health authority study registry websites, as required by local health authority regulations.

16 REFERENCES

1. Mansmann G, Lau J, Balk E, Rothberg M, Miyachi Y, Bornstein SR. The clinically inapparent adrenal mass: update in diagnosis and management. Endocr Rev 2004; 25, 309-340
2. Boland GW, Dwamena BA, Jagtiani Sangwaiya M, Goehler AG, Blake MA, Hahn PF, Scott JA, Kalra M K. Characterization of adrenal masses by using FDG PET: a systematic review and meta-analysis of diagnostic test performance. Radiology 2011; 259: 117-126
3. Hahner S, Sundin A. Metomidate-based imaging of adrenal masses. Horm Cancer 2011; 2: 348-253
4. Hahner S, Kreissl MC, Fassnacht M, Haenscheid H, Bock S, Verburg FA, Knoedler P, Lang K, Reinser C, Buck AK, Allolio B, Schirbel A. Functional characterization of adrenal lesions using 123I-IMTO-SPECT/CT. J Clin Endocrinol Metab 2013; 98 (4): 1508-1518
5. Hahner S, Kreissl MC, Fassnacht M, Haenscheid H, Knoedler P, Lang K, Buck AK, Reiners C, Allolio B, Schirbel A. [131I]Iodometomidate for Targeted Radionuclide Therapy of Advanced Adrenocortical Carcinoma. J Clin Endocrinol Metab 2012; 97(3):914-922.
6. Hahner S, Stuermer A, Kreissl M, Reiners C, Fassnacht M, Haenscheid H, Beuschlein F, Zink M, Lang K, Allolio B, Schirbel A. [123 I]Iodometomidate for molecular imaging of adrenocortical cytochrome P450 family 11B enzymes. J Clin Endocrinol Metab 2008; 93, 2358-2365
7. Kreissl MC, Schirbel A, Fassnacht M, Haenscheid H, Verburg FA, Bock S, Saeger W, Knoedler P, Reiners C, Buck AK, Allolio B, Hahner S. 123I-Iodometomidate Imaging in Adrenocortical Carcinoma. J Clin Endocrinol Metab 2013; Apr 22
8. Boland GW, Lee MJ, Gazelle GS, Halpern EF, McNicholas MM, Mueller PR. Characterization of adrenal masses using unenhanced CT: an analysis of the CT literature. AJR Am J Roentgenol 1998; 171: 201-204
9. Shulkin B L; Thompson N W; Shapiro B; Francis I R; Sisson J C 1999 Pheochromocytomas: imaging with 2-[fluorine-18]fluoro-2-deoxy-D-glucose PET. Radiology 212, 35.
10. Han SJ, Kim TS, Jeon SW, Jeong SJ, Yun M, Rhee Y, Kang ES, Cha BS, Lee EJ, Lee HC, Lim SK. Analysis of adrenal masses by 18F-FDG positron emission tomography scanning. Int J Clin Pract 2007; 61: 802-809
11. Juhlin C, Tornblom S, Rastad J, Bergstrom M, Bonasera T, Sundin A, Langstrom B. Differential diagnosis in adrenal gland tumors using PET and 11C-metomidate]. Nord Med 1998; 113, 306-307
12. Minn H, Salonen A, Friberg J, Roivainen A, Viljanen T, Langsjo J, Salmi J, Valimaki M, Nagren K, Nuutila P. Imaging of adrenal incidentalomas with PET using (11)C-metomidate and (18)F-FDG. J Nucl Med 2004; 45: 972-979
13. Hennings J, Lindhe O, Bergstrom M, Langstrom B, Sundin A, Hellman P. [11C]metomidate positron emission tomography of adrenocortical tumors in correlation with histopathological findings. J Clin Endocrinol Metab 2006; 91, 1410-1414
14. Zettinig G, Mitterhauser M, Wadsak W, Becherer A, Pirich C, Vierhapper H, Niederle B, Dudczak R, Kletter K. Positron emission tomography imaging of adrenal masses: (18)F-fluorodeoxyglucose and the 11beta-hydroxylase tracer (11)C-metomidate. Eur J Nucl Med Mol Imaging 2004 ; 31 : 1224-1230

15. Brix G, Lechel U, Glatting G, Ziegler SI, Münzing W, Müller SP, Beyer T. Radiation exposure of patients undergoing whole-body dual-modality 18F-FDG PET/CT examinations. J Nucl Med. 2005; 46(4):608-613.
16. Kreissl MC, Schirbel A, Fassnacht M, Haenscheid H, Verburg FA, Bock S, Saeger W, Knoedler P, Reiners C, Buck AK, Allolio B, Hahner S. 123I-Iodometomidate Imaging in Adrenocortical Carcinoma. J Clin Endocrinol Metab 2013; Apr 22
17. Funder JW, Carey RM, Mantero F, Murad MH, Reincke M, Shibata H, Stowasser M, Young WF Jr. The Management of Primary Aldosteronism: Case Detection, Diagnosis, and Treatment: An Endocrine Society Clinical Practice Guideline. J Clin Endocrinol Metab. 2016 May;101(5):1889-916
18. Hillner BE; Siegel BA, Liu D, Shields AF, Gareen IF, Hanna L, Stine SH, Coleman RE. Impact of positron emission tomography/computed tomography and positron emission tomography (PET) alone on expected management of patients with cancer: initial results from the National Oncologic PET Registry. J Clin Oncol 2008; 26 (13): 2155-2161
19. Fassnacht M, Arlt W, Bancos I, Dralle H, Newell-Price J, Sahdev A, Tabarin A, Terzolo M, Tsagarakis S, Dekkers OM. Management of adrenal incidentalomas: European Society of Endocrinology Clinical Practice Guideline in collaboration with the European Network for the Study of Adrenal Tumors. Eur J Endocrinol. 2016 Aug;175(2):G1-G34
20. Weiss, LM, Medeiros, LJ, Vickery, AL. Pathologic features of prognostic significance in adrenal cortical carcinoma. Amer J Surg Pathol 1989; 13. 202-206
21. van Slooten H, Schaberg A, Smeenk D, Moolenaar AJ. Morphologic characteristics of benign and malignant adrenocortical tumors. Cancer 1985; 55: 766-773
22. Hough AJ, Hollifield JW, Page DL, Hartmann WH. Prognostic factors in adrenal cortical tumours. Am J Clin Pathol 1979; 72: 390-399
23. Kimura N, Watanabe T, Noshiro T, Shizawa S, Miura Y. Histological grading of adrenal and extra-adrenal pheochromocytomas and relationship to prognosis: a clinicopathological analysis of 116 adrenal pheochromocytomas and 30 extra-adrenal sympathetic paragangliomas including 38 malignant tumors. Endocr Pathol 2005; 16: 23-32
24. Thompson LDR. Pheochromocytoma of the adrenal gland scaled score (PASS) to separate benign from malignant neoplasms - A clinicopathologic and immunophenotypic study of 100 cases. Amer J Surg Pathol 2002; 26: 551-566

17 APPENDIX

17.1 Grade ECOG (Eastern Cooperative Oncology Group)

0	Fully active, able to carry on all pre-disease activities without restriction
1	Restricted in physically strenuous activity but ambulatory and able to carry out work of a light or sedentary nature, e.g., light house work or office work
2	Ambulatory and capable of all selfcare but unable to carry out any work activities. Up and about more than 50% of waking hours
3	Capable of only limited selfcare, confined to bed or chair more than 50% of waking hours
4	Completely disabled. Cannot carry on any selfcare. Totally confined to bed or chair
5	Dead

17.2 Standard Operating Procedures for Collection and Analysis of Pathology Data

The adrenal lesion removed during surgery will be handled by the local pathologist in a standardized fashion: fixation in formalin and embedding of tumor tissue in paraffin. The local pathologist will then provide a report on the diagnosis to the respective unit which has sent the material.

After completion of the report by the local pathologist the paraffin block of the resected tumor tissue will be forwarded by the local pathologist to **one** of the reference pathologists of the FAMIAN study:

- ***Prof. Dr. Andreas Rosenwald, Pathologisches Institut der Universität Würzburg, Josef-Schneider-Str. 2. D-97080 Würzburg, Germany (Phone: +49 931 31-81247 or +49 931 31 -811199; Fax: +49 931 31-81224)***
- ***Prof. Dr. Wolfgang Saeger, Neuropathologie der Universität Hamburg, Martinistr. 52, D-20246 Hamburg, Germany (Phone +49 40 741052218)***.

Analysis by the reference pathologist follows a strict algorithm: haematoxylin-eosin staining will be performed for all provided blocks. The best suited tissue block will be used for further specialized stainings: PAS, MG and immunohistologial analysis.

Pancreatin KL1, synaptophysin, chromogranin A, steroidogenic factor 1, melan A, and Ki67 immunohistochemistry will be performed. In case of an assumed pheochromocytoma in addition S100-protein and p53 immunohistochemistry will be performed.

In case of suspected metastasis the following cytokeratin markers will be used: CK 5, CK 7, CK 20 and further tissue markers as needed (e. g. TTF-1, CDX-2 or RCC, in case of suspected sarcoma smooth muscle aktin, desmin and vimentin, in case of suspected melanoma metastasis Hmb-45).

After immunohistological differentiation in cortical, medullary and metastatic lesions the adrenal tumor will be classified.

Differentiation of benign and malignant lesions: in case of an adrenocortical tumor the Weiss score (20) will be used for diagnosis of malignancy. In cases of ongoing uncertainty, the additional scoring systems of *van Slooten et al.* (21) and *Hough et al.* (22) will be used. In case of a non-secretory pheochromocytoma assessment of malignancy will be based on the scoring systems of *Kimura et al.* (23) and *Thomson et al.* (24).

The results of the analysis by the reference pathologist are documented in a standardized assessment form. A copy of this form with the pertinent data will be sent to both the coordinating investigator (Prof. Dr. S. Hahner, Würzburg) and to the respective local pathologist. A representative tissue block will remain in the archive of the reference pathologist for further analysis, if needed. The remaining paraffin blocks will be sent back to the respective local pathologist.

17.3 Standard Operating Procedures for [123I]-Iodometomidate Imaging and FDG-PET/CT within the FAMIAN trial

17.3.1 [^{123}I]Iodometomidate scintigraphy (planar imaging and SPECT/CT)

1) **Indication**
Differentiation of adrenal masses (>3cm) in adrenocortical and non-adrenocortical neoplasias

2) **Pathophysiological principle**
Radioiodinated iodometomidate (IMTO) binds highly selective to the target enzymes 11ß-hydroxylase and aldosterone synthase, which play a crucial role in the biosynthesis of cortisol and aldosterone. Both enzymes are exclusively expressed in adrenocortical tissue. IMTO thereby shows a very high and specific uptake but is metabolized rapidly. The tracer is mainly excreted by the kidneys and only to a small amount by the hepatobiliary system.

3) **Application of the radiotracer**
   - Injection of 185 MBq [^{123}I]Iodometomidate via venous catheter
   - Flushing of the venous catheter with 8-10 ml 0.9% NaCl after injection of the radiotracer
   - Measurement of residual radioactivity in the syringe
   - Documentation of the injected activity (activity in the syringe prior to injection – residual radioactivity).

4) **Preparation of the radiotracer**
   - [^{123}I]Iodometomidate
   - GMP-compliant preparation according to the published protocol (Schirbel et al, Radiochimica Acta **92** (2004) 297-303)
   - [^{123}I]Iodometomidate can be stored at room temperature from receipt to injection (on the same day)

5) **Quality control**
   - The quality control is performed by the radiochemistry unit according to current GMP standards.

6) **Gamma-camera / SPECT/CT camera**
   - Dual-head gamma camera (e.g., Siemens e.cam or similar device)
   - SPECT/CT camera (e.g., Siemens Symbia T2 or similar device)
     SPECT/CT is a combination of a CT scanner and a dual-head gamma-camera and enables the fusion of high resolution metabolic, functional and morphological data. In addition, the CT data serve as morphological reference in fused images, further ensuring the correct allocation of focal tracer retention to a corresponding organ or tissue.

7) **Procedure**
   - Patient briefing, documentation
   - Insertion of a venous catheter
   - Thyroid blocking by oral administration of 1.150 mg sodium perchlorate 15-20 min (e.g., Irenat) prior to the injection of [^{123}I]Iodometomidate

- Continuation of thyroid blocking for 1-3 days using 3 daily doses of 460 mg sodium perchlorate. (Use of potassium iodid or other perchlorate preparation is allowed where sodium perchlorate is not available)

8) Data acquisition and imaging

- 0 – 5 min p.i. (optional) — dynamic measurement of upper abdomen/adrenal region; scan duration: 10 sec/image; planar, dorsal and ventral view
- 4 – 6 h p.i. — whole body imaging; planar; dorsal and ventral view; scan velocity: 20 cm/min; with activity standard (ca. 1 MBq ^{123}I at time of injection)
- 4 – 6 h p.i. — SPECT/CT upper abdomen/adrenal region before or after planar whole-body imaging
- 22 – 26 h p.i. — whole body imaging; planar; dorsal and ventral view recommended; scan velocity: 20 cm/min; with activity standard (ca. 1 MBq ^{123}I at time of injection)
- 22 – 26 h p.i. — SPECT/CT upper abdomen/adrenal region before or after planar whole-body imaging

Note: Data acquisition is mandatory at least within one of the timeframes 4-6 hours or 22-26 hours.

9) Patient positioning

- Supine position, ´feet in`
- Arms in a resting position next to the body.

10) Scanner parameters

Planar scintigraphy (total body)

Parameter	required	recommended
Collimator	collimator with low septal penetration	medium-energy collimator
window setting: 159 keV	+/- 7,5% - +/- 10%	+/- 7,5%
scan velocity 4–6 h p.i.	≤ 20 cm/min	20 cm/min
22–26 h p.i. (optional)		10 cm/min
pixel size	max. (3 mm)²	(2 mm)²

SPECT/CT (vertebra T11 ± 20 cm)

Parameter	required	recommended
Collimator	collimator with low septal penetration	medium-energy collimator
window setting: 159 keV	+/- 7,5% - 10%	+/- 7,5%
Orbit		auto-contour
scan arc	360°	2x 180°
angular step	≤ 3°	3° or 2.8°
no. of views	120-128	2x 60 or 2x 64

total imaging time	20 – 30 min	30 min
matrix size	128 x 128	
Reconstruction	expectation maximization (EM)	ordered subset (OS)-EM
no. of subsets		4 – 16
subsets x iterations	24	≥ 32
filtering		3 – 8 mm kernel
Corrections	attenuation (CT-based)	scatter<br>collimator and detector response
$CTDI_{vol}$	≤ 3 mGy	2 mGy
dose length product (DLP)	≤ 120 mGy·cm	80 mGy·cm

11) Waste management

- ^{123}I has a physical half-life of 13.21 h; the radioactive waste will be collected in an appropriate container, stored and disposed according to radiation protection law

12) Effective dose

- The effective dose results from the radiotracer [^{123}I]Iodometomidate and, to a lesser extent, from low-dose CT
- Application of 185 MBq [^{123}I]Iodometomidate (2.4 mSv) and two low-dose CT (1.8 mSv each) results in a maximum effective dose of 6.0 mSv.

13) Documentation

- The nuclear physician documents patient history, type of radiotracer and written informed consent.
- The technician documents radionuclide, radiopharmaceutical, manufacturer, radioactivity of the syringe before and after tracer injection, time point and site of injection.
- Subsequently, the documentation will be completed and saved electronically (e.g., in the central information system of the hospital).
- CT-Scan: The dose-length-product DLP (product of $CTDI_{vol}$ and total scan length) of every CT scan and, if applicable, administration of contrast agent must be documented.
- After investigation of the patient, all images have to be archived electronically (e.g., RIS-PACS). In addition, a hard-copy (CD-R, DVD) has to be filed containing all data acquisitions in DICOM format.

17.3.2 18F-FDG-PET/CT

1) Clinical Indication

Determination of malignancy of adrenal tumors (>3cm)

2) Pathophysiological Principle

18F-Fluorodeoxyglucose (FDG) is a radiolabelled glucose analogue which is taken up by cells using glucose as primary energy source. Glucose use is increased in many malignant tumors and also in inflammatory processes resulting in an increased FDG uptake. After intravenous injection, FDG is quickly cleared from the blood pool via glucose transporters located in the cellular membrane. Within cells, FDG undergoes rapid phosphorylation to fluordeoxyglucose-6-phosphate by the enzyme hexokinase II and is retained in the cell (trapping). Due to the various uptake patterns of FDG in adrenal tumors observed in past series, we hypothesize that high FDG uptake is a prerequisite of malignant adrenal tumors (malignant primaries, adrenal metastasis), whereas benign tumors (i.e., adenoma, incidentaloma) are associated with missing or mild retention of FDG.

IMPORTANT: Cellular uptake of FDG is insulin-dependent and therefore influenced by food intake, the nutritional condition of the patient and presence of diabetes mellitus.

3) Injected Activity

Injected activity in a person with normal weight (70 kg) is recommended to be < 350 MBq FDG, according to recently published German diagnostic reference standards. The effective dose for an injected activity of 350 MBq FDG has been calculated to be as high as 6.7 mSv per examination (according to Medical Internal Radiation Dose, MIRD). A permitted protocol alteration regarding larger FDG activities includes patient obesity. Of note, ALARA principles (as low as reasonly achievable) need to be considered.

4) Availability of FDG

FDG is delivered daily as sterile, clear and ready-to-use liquid. It can be stored at room temperature before injection.

5) Quality Control

Quality control is performed by the provider or the in-house manufacturer (local radiochemistry / cyclotron unit) according to current GMP standards.

6) PET/CT-Scan

New generation hybrid PET/CT scanners with a multi-slice CT component have to be utilized for imaging. An integrated PET/CT system is a combination of a PET and a CT scanner with a single patient table. PET/CT allows a sequential acquisition of corresponding PET and CT portions of the examination without changing the positioning of the patient. Both data sets are intrinsically co-registered, given that the patient does not move during or in between the acquisitions. The PET+CT fusion is the mechanical and data related fusion of PET and CT volume data sets in a combined data set. A fused PET+CT data set allows the combined visualization of the fused PET and CT datasets.

7) Patient Preparation

Patients are asked to fast at least 6 hours prior to the injection of FDG, refraining from food intake or liquids including sugar. Non-sweetened tea or water is allowed. Adequate hydration is requested. Parenteral nutrition and/or glucose infusion have to be stopped also 6 hours prior to tracer injection.

Within the FAMIAN study only a low dose CT scan will be performed. However, full dose CT can be performed for diagnostic purposes at the discretion of the investigator to avoid additional radiation exposure by a separate imaging procedure.

Use of CT contrast media is only legitimate if up-to-date creatinine- and TSH-levels are available. Moreover, allergies to iodine based contrast media have to be excluded. In case of increased creatinine level, either no CT contrast media can be given, or patients have to undergo kidney-protective treatment.

<u>Prior to injection of FDG</u>

Patient's blood glucose has to be determined and an indwelling intravenous device has to be installed. A nuclear medicine physician will then collect the history of the patient, explain the scan procedure (including use and side effects of CT contrast media if necessary), approve the indication of the scan and finally document informed consent of the patient. Use of diuretics (i.e., furosemide: 10 mg Lasix i.v.) is allowed to reduce bladder activity and radiation exposure to the bladder. There is no reason for routine administration of sedatives, however, if necessary its use is allowed.

During the injection of FDG and subsequent uptake phase, the patient should remain seated or recumbent and silent to minimize FDG uptake in muscles. The patient should be kept warm starting at 30–60 min before the injection of FDG and throughout the following uptake period and PET examination to minimize FDG accumulation in brown fat tissue (especially relevant if the room is air conditioned).

Blood glucose level must be measured prior to administering FDG. A glucometer or a similar bedside device (capable of performing overall euglycemia measurements) can be used for this purpose, whereas a blood glucose test must be performed using a calibrated and validated method, if plasma glucose levels are used as correction of SUV measurements.

If plasma glucose level is ≥150 mg/dl, the FDG-PET study must be postponed and performed at a later time point after repetitive measurement of blood glucose levels. If appropriate glucose levels cannot be achieved, the examination has to be rescheduled. Reduction of blood glucose levels by administration of insulin can be considered, but the PET/CT examination should be postponed (no earlier than 90 min after insulin injection with stable blood glucose levels), depending on the type and route of the administration of insulin.

8) Image Acquisition

Image acquisition will be started 60 min (acceptable window: 55 to 75 min) after injection of FDG.

9) Patient positioning

Prior to positioning in the gantry of the scanner, the patient is asked to void the bladder to reduce radiation exposure to the bladder and the urinary tract, and to improve image quality. Patients are asked to take off all metal containing belongings (e. g. watches, belts) to reduce image artifacts.

Patient position is - head first supine -. The patient should be positioned with the arm elevated over the head to avoid beam hardening artifacts as well as artifacts caused by truncation of the field of view. If this position is not tolerated by the patient, the arms can be positioned along the side. Instruct the patient to not move or speak and to try to avoid excessive swallowing movements.

10) Scan duration

The scan duration ranges from 15 to 20 minutes (PET: 12 – 15 min; CT 3 – 5 min).

11) Low-dose PET/CT (´whole body´)

A low-dose PET/CT scan covering the neck, thorax, abdomen and pelvis will be performed. The standard acquisition protocol (e.g. PET_AC_Wholebody_HD) routinely used for whole-body FDG-PET/CT studies can be used.

They should be conform with the EANM procedure guidelines for tumor imaging (Version 2.0: http://www.eanm.org/publications/guidelines/2015_GL_PET_CT_TumorImaging_V2.pdf)

Scan protocol:

CT: vertex – mid thigh, DLP ≤ 300 mGy·cm (recommended parameters: 120 kV, 30 mAs),

PET: vertex – mid thigh

NOTE: Full dose CT can be performed for diagnostic purposes at the discretion of the investigator to avoid additional radiation exposure by a separate imaging procedure. Scan may follow the standard acquisition protocol for full dose CT generally used in the participating center. In case of administration of contrast agent, the acquisition of contrast-enhanced abdominal CT will be performed before the low-dose PET/CT scan without repositioning of the patient or additional injection of i.v.-contrast material.

12) Documentation

Prior to performing PET/CT scans, the physician has to collect the patient history and to determine the injected activity. All data have to be documented in written format. Moreover, the following data have to be recorded:

- applied activity, purity, injection type and site (localization of injection)
- time of injection, uptake time
- body weight (for each longitudinal study) and height, gender
- Information concerning medication administered as preparation of the PET scan
- Field of view and patient positioning: whole-body PET/CT, skull base to mid-thigh, limited area and position of the arms
- Blood glucose level before the examination and used methodology to obtain blood glucose
- CT-protocol: low-dose (including DLP) or/and diagnostic CT, contrast agent application (oral, intravenous, information on concentrations and volumes, native, arterial, portal-venous phase), scanned portion of the body

At the end of the scan the PET/CT data have to be digitally archived in DICOM format (both clinical PACS and DVD).

17.4 Standard Operating Procedures for Collection, Storage, and Shipping of Biological Specimens

Urine steroidobolomics will be investigated as a tool to characterize indeterminate adrenal lesions with a diameter > 3 cm. To this end 24-hour urine will be collected and a representative aliquot will be sent to Birmingham for analysis by LC/MS.

Biomaterial Collection

- Collections should be performed using plain collection bottles without addition of preservatives.
- In women, avoid collections during menstrual bleeding
- The collection should be returned to hospital on the day of completion.If this is not possible, it is acceptable to store the bottle for up to 3 days at 4°C (in the fridge).
- Date of collection and volume of 24-hour urine should be noted.

Biomaterial Processing

- Gently invert the collection bottle several times.
- If a subject provides a sample of more than 3000mL, i.e. more than one bottle, it is important to mix the two volumes well before taking an aliquot.
- Transfer two aliquots of at least 5 ml into urine collection tubes.
- Freeze the samples immediately at -20°C
- One sample should be stored locally on site and one sample should be sent to Würzburg (German centres) or directly to Birmingham (Non-German Ens@t centres). Further shipping information is given below.

Biomaterial Labeling & Storage

- Each sample should be labelled with the unique FAMIAN patient identifier, the date the sample was taken and the volume of the 24-hour urine. Without this information samples will NOT be processed.
- Ensure that all labelling is waterproof and resistant to cold storage conditions.
- Urine samples need to be frozen immediately at -20°C and stored ideally at -80°C (-20°C is also acceptable for up to 12 months).
- Once frozen, avoid thawing of samples.
- Handle and transport on dry ice (see below).

Biomaterial Shipment

- Once you have accumulated the samples of several FAMIAN patients, you can prepare the shipment.
- Samples should not thaw during transportation – please use dry ice in insulated boxes for shipment to Birmingham. Only for the shipping of **frozen** material within Germany to Würzburg, a padded envelope will suffice if it is ensured that there will be somebody on site to take care of the shipment the next day.

- **Non-German ENS@T centres:** Please contact the Birmingham centre to ensure there is somebody in the lab that week to receive the parcel and send samples preferably on Mondays: Angela Taylor (a.taylor.5@bham.ac.uk); cc Wiebke Arlt (w.arlt@bham.ac.uk)
  - Please use dry ice in insulated boxes
  - Ship the samples early in the week, to avoid long periods without replacement of dry ice.
  - Please provide the tracking number and courier information.
  - Birmingham will confirm safe arrival upon receipt of the parcel.

  Samples from Non-German ENS@T centres should be shipped to:

  Steroid Metabolome Analysis Core

  To the attention of Dr. Angela Taylor

  Institute of Metabolism and Systems Research

  Medical School IBR Tower Level 2, Rm 238

  University of Birmingham

  Edgbaston, Birmingham, B15 2TT

  United Kingdom

  Fax +44 121 415-8712

  Phone +44 121 414-3768

- **German centres:** Samples will be collected in Wuerzburg and later sent to the University of Birmingham. Please contact the Wuerzburg centre to ensure that the specimen can be received and processed the next day: Martina Zink (zink_m@ukw.de) and famian@ukw.de
  If this is guaranteed and if you send frozen samples a padded envelope will suffice and there is no need for dry ice.

  Samples from German centres should be shipped to:

  Universitätsklinikum Würzburg

  Medizinische Klinik und Poliklinik I

  Endokrinologie und Diabetologie

  Martina Zink - FAMIAN

  A4.-2.969

  Oberdürrbacher Str. 6

  97080 Würzburg

  Fax +49 931 201-639716

  Phone +49 931 201-39716

17.5 National Cancer Institute - Common Terminology Criteria for Adverse Events

The NCI CTCAE (version 4.0) has been placed in the Study Reference Binder for this protocol. Alternatively, the NCI CTCAE may be reviewed online at the following NCI website:

NATIONAL CANCER INSTITUTE

Cancer Therapy Evaluation Program

http://ctep.cancer.gov/

(https://evs.nci.nih.gov/ftp1/CTCAE/CTCAE_4.03/CTCAE_4.03_2010-06-14_QuickReference_5x7.pdf)

17.6 SAE (Serious Adverse Event) Form

ZKS Würzburg	Clinical Trial<br>Serious Adverse Event Report	☐ New<br>☐ Follow-up

EUDRACT No. :

**PROTOCOL IDENTIFICATION:**	Combined 18F-Fluorodeoxyglucose (FDG) Positron Emission Tomography (PET) and Metomidate Imaging for Adrenal Neoplasia (**FAMIAN-Study**)
**INDICATION:**	Adrenal Neoplasia
**SPONSOR:**	

SELECT OR TYPE IN A FAX#

1. SITE INFORMATION

Site Number	Investigator	Country
Reporter	Phone Number	Fax Number

2. SUBJECT INFORMATION

Subject ID Number	Sex	Age (years)	Height (cm)	Weight (cm)	SAE-No.
	☐F ☐M				

3. SERIOUS ADVERSE EVENT – *Information in this section must also be entered on the Serious Adverse Event Summary CRF*

**Serious Adverse Event Diagnosis or Syndrome If diagnosis is unknown, enter Signs/Symptoms When Final Diagnosis is known, enter ad Adverse Event**<br>List one event per line. If event is fatal, enter the Cause of Death. Entry of Death is not acceptable, as this is an outcome.	**Date Started**<br>Day Month Year	**Date Ended**<br>Day Month Year	**Time of SAE**	**Check only if event oc-curred before first dose of IP**	**Enter Serious Criteria code (see codes below)**	**Relationship Is there a reasonable possibility that the event may have been caused by IP If yes, see section 10**<br>No√	 Yes√	**Outcome of Event**<br>01 Resolved<br>02 Resolving<br>03 Not resolved<br>04 Fatal	**Check only if event is related to study procedure**<br>e.g. biopsy

**Serious Criteria:**	01 Fatal<br>02 Immediately life-threatening	03 Required hospitalization<br>04 Prolonged hospitalization	05 Persistent or significant disability/incapacity<br>06 Congenital anomaly / birth defect	07 Other significant medical hazard

4. HOSPITALIZATION

	Date Admitted<br>Day Month Year	Date Discharged<br>Day Month Year
Was subject hospitalized? ☐ No ☐ Yes, If yes, please complete date(s)		

5. INVESTIGATIONAL PRODUCT (IP)

	Time of IPA	Initial Start Date<br>Day Month Year	Prior to, or at time of Event<br>Date of Dose<br>Day Month Year	Dose	Route	Frequency	Action Taken with Product<br>01 Still being administered<br>02 Permanently discontinued<br>03 Withheld
< IP ><br>☐ Blinded							

6. RELEVANT CONCOMITANT MEDICATIONS (e.g. chemotherapy) Any Relevant Medications? ☐ No ☐ Yes, If yes, please complete:

Medication Name(s)	Start Date	Stop Date	Co-suspect		Continuing		Dose	Route	Freq.	Treatment Med.	
	Day Month Year	Day Month Year	No√	Yes√	No√	Yes√				No√	Yes√

ZKS Würzburg	Clinical Trial<br>Serious Adverse Event Report	☐ New<br>☐ Follow-up

EUDRACT No. :

PROTOCOL IDENTIFICATION: Combined 18F-Fluorodeoxyglucose (FDG) Positron Emission Tomography (PET) and Metomidate Imaging for Adrenal Neoplasia (**FAMIAN-Study**)

INDICATION: Adrenal Neoplasia

SPONSOR:

	Site Number	Subject ID Number	

7. RELEVANT MEDICAL HISTORY (include dates, allergies and any relevant prior therapy)

8. RELEVANT LABORATORY VALUES (include baseline values) Any Relevant Laboratory values? ☐ No ☐ Yes, If yes, please complete

Date	Test											
	Unit											
Day Month Year												

9. OTHER RELEVANT TESTS (diagnostics and procedures) Any other relevant tests? ☐ No ☐ Yes, If yes, please complete:

Date<br>Day Month Year	Additional Tests	Results	Units

10. CASE DESCRIPTION (Provide narrative details of events listed in section 3) For each event in section 3, where Relationship = Yes, please provide rationale.

Signature of Investigator or Designee	Title	Date

Page 2 of 2

17.7 Signature Page

« Combined ^{18}F-Fluorodeoxyglucose (*F*DG) Positron Emission Tomography (PET) *a*nd *M*etomidate Imaging for *A*drenal *N*eoplasia (FAMIAN-Study) » - a Diagnostic Study

Sponsor Protocol N°: FAMIAN-1

EudraCT N°: 2012-003604-13

Version 1.7 (26 MAY 2020)

I, Dr/Prof__________________________________, certify that I have read the entire protocol entitled **"Combined 18F-Fluorodeoxyglucose (*F*DG) Positron Emission Tomography (PET) *a*nd *M*etomidate *I*maging for *A*drenal *N*eoplasia** (FAMIAN-**Study)"** and I agree to conduct the study according to this protocol and to comply with requirements subject to ethical and safety considerations.

Date: ____________________ **Signature:** ________________________________

Name: ________________________________

Name and address of centre: ____

____

____

____
