# Supplementary material for: Combined [18F]Fluorodeoxyglucose PET and [123I]Iodometomidate-SPECT for diagnostic evaluation of indeterminate adrenal neoplasias—the cross-sectional diagnostic test accuracy study FAMIAN
Source: eBioMedicine. 2025 May 20;116:105735. doi: 10.1016/j.ebiom.2025.105735 (PMC12148602; doi:10.1016/j.ebiom.2025.105735)
Supplement: Study group collaborators [file mmc4.docx]

FAMIAN Study Group, collaborators not listed among authors

| **Further contributors/collaborators** |  |  |
| --- | --- | --- |
| **Name** | **Surname** | **affiliation** |
| **Christian** | **Furth** | Charité - Universitätsmedizin Berlin, Freie Universität Berlin, Humboldt-Universität zu Berlin, and Berlin Institute of Health, Department of Nuclear Medicine. Germany |
| **Knut** | **Mai** | Charité-Universitätsmedizin Berlin, Corporate member of Freie Universität Berlin, Humboldt-Universität zu Berlin, and Berlin Institute of Health, Department of Endocrinology and Metabolism. Germany |
| **Marcus** | **Quinkler** | Endocrinology in Charlottenburg, Berlin. Germany |
| **Frank** | **Weber** | Department of General, Visceral and Transplantation Surgery, Division of Endocrine Surgery, University of Duisburg-Essen. Germany |
| **Henning** | **Dralle** | Department of General, Visceral and Transplantation Surgery, Division of Endocrine Surgery, University of Duisburg-Essen. Germany |
| **Benjamin** | **Sandner** | Medizinische Klinik und Poliklinik III – Endokrinologie, Nephrologie, Rheumatologie, Universitätsklinikum Leipzig. Germany |
| **Thomas** | **Lincke** | Klinik und Poliklinik für Nuklearmedizin, Universitätsklinikum Leipzig Germany |
| **Regine** | **Kluge** | Klinik und Poliklinik für Nuklearmedizin, Universitätsklinikum Leipzig.Germany |
| **Nada** | **Rayes** | Klinik und Poliklinik für Viszeral‑, Transplantations‑, Thorax- und Gefäßchirurgie, Universitätsklinikum Leipzig. Germany |
| **Matthias** | **Weber** | Department of Endocrinology and Metabolism, I Medical Clinic, University Medical Center of the Johannes Gutenberg University Mainz |
| **Martina** | **Gräsl** | Department of Internal Medicine I, Division of Endocrinology and Diabetes, University Hospital, University of Würzburg. Germany |
| **Wolfgang** | **Saeger** | Institute of Pathology, University Medical Center Hamburg-Eppendorf, Hamburg. Germany |
| **Joachim** | **Reibetanz** | Department of General-, Visceral-, Transplant-, Vascular- and Pediatric Surgery, University Hospital of Wuerzburg, Wuerzburg, Germany |
